# Metabarcoding of Soil Fungi from Different Urban Greenspaces Around Bournemouth in the UK

**DOI:** 10.1007/s10393-021-01523-1

**Published:** 2021-06-05

**Authors:** Emma L. Marczylo, Sameirah Macchiarulo, Timothy W. Gant

**Affiliations:** Toxicology Department, Centre for Radiation, Chemical and Environmental Hazards, Public Health England, Harwell Campus, Chilton, Oxfordshire OX11 0RQ UK

**Keywords:** Metabarcoding, Fungi, Soil, Urban greenspaces, Public health, High throughput sequencing

## Abstract

Soil microbes are important for public health. Increasing urbanisation is adversely affecting soil microbiota, which may be contributing to the global rise of immune-related diseases. Fungi are key components of urban environments that can be negatively impacted by altered land-use, land-management and climate change, and are implicated in the development and exacerbation of non-communicable diseases such as allergy, asthma and chronic inflammatory conditions. Fungal metagenomics is building knowledge on fungi within different environments (the environmental mycobiome), fungi on and within the human body (the human mycobiome), and their association with disease. Here, we demonstrate the added value of a multi-region metabarcoding approach to analyse soil mycobiomes from five urban greenspaces (lawns, parklands, bareground, young forest and old forest). While results were comparable across the three regions (ITS1, ITS2 and LSU), each identified additional fungal taxa that were unique to the region. Combining the results therefore provided a more comprehensive analysis across all fungal taxonomic ranks, identifying statistically significant differences in the fungal composition of the five soil types. Assignment of fungal taxa into ecological guilds revealed those differences of biological relevance to public health. The greatest differences were between the soil mycobiome of lawns and forests. Of most concern was the significant increase in the known human allergens *Alternaria*, *Bipolaris*, *Cladosporium* and *Fusarium* within urban lawn and parkland *vs* forest soils. By improving our understanding of local variations in fungal taxa across urban greenspaces, we have the potential to boost the health of local residents through improved urban planning.

**Supplementary Information:**

The online version contains supplementary material available at 10.1007/s10393-021-01523-1.

## Introduction

Soil microbiota are increasingly recognised as an important consideration for public health, as reviewed in (Wall et al. 2015; Hirt 2020). Much of the soil ecosystem is microbial, either as a natural (permanent) inhabitant of the soil or as a transient (temporary) inhabitant awaiting transmission to a host through direct contact. Natural soil microbes, particularly bacteria and fungi, fulfil a vital ecological role as decomposers and nutrient recyclers. Both bacteria and fungi are widespread in soil across the globe, with different species being suited to different local environments and climates (Tedersoo et al. 2014; Delgado-Baquerizo et al. 2018).

As detailed in (Wall et al. 2015; Wall and Six 2015; Hirt 2020), some of these soil microbes promote food production, forming complex symbiotic relationships within a crop’s root system to enhance nutrient uptake and/or to help control pests or pathogens. Others act as natural or transient soil-borne crop pathogens. The balance of growth promoting *vs* growth inhibiting microbes can therefore have a significant effect on both the quantity and nutritional quality of these crops. Soil microbes can also degrade pollutants and maintain a soil structure for enhanced water infiltration and percolation, with potential impacts on the quantity and quality of local surface water and groundwater. Through both natural (rain, wind, convection) and human activities (farming, composting, burning of plant biomass), soil microbes can also be aerosolised into bioaerosols. Such bioaerosols can be carried hundreds to thousands of miles away from the original source, dispersing microbes across wider environments as well as increasing levels of inhalable, biologically active particulate matter (PM) < 10 μm. Soil microbes, particularly fungi, play a key role in stabilising soil structure and help to limit potential aerosolisation. Thus, in the context of public health, soil microbes influence the quantity and/or quality of the food we eat, the water we drink and the air we breathe.

Soil microbes can also have a more direct effect on human health. Pathogenic microorganisms within our environment can cause infectious disease. Most soil microbes, however, are not pathogenic. Yet they can still influence our health. Humans have co-evolved alongside the microbes in their environment, with soil microbes comprising a significant component of this so-called environmental microbiome. We are therefore continually exposed to soil-derived bacteria, fungi and their associated components throughout our indoor and outdoor environments. Such exposures, particularly in early life, are vital for the normal development of our immune systems. Indeed, children who grow up in environments with reduced microbial exposures are more likely to suffer from allergies and asthma; imbalances in the microbes within the human gut have been associated with allergies, asthma and other non-communicable diseases (NCDs) such as diabetes, cardiovascular disease, obesity, neurodevelopmental disorders and mental health; and greater contact with environmental microbes has been linked to enhanced protection against infectious disease (Ege et al. 2011; Dietert and Dietert 2015; Rieder et al. 2017; Stiemsma and Michels 2018; Mills et al. 2019; Yamashiro et al. 2019). In addition to colonising the human gut, environmental microbes can also colonise the human skin, reproductive system and respiratory tract. The collection of microbes living on or within the human body is termed the human microbiome. While much of the research to-date has focussed on bacteria, there is increasing evidence that common soil fungi (such as *Alternaria*, *Aspergillus*, *Candida*, *Cladosporium Cryptococcus*, *Trichosporon* and *Penicillium*) can also colonise the human body and/or elicit immune responses, impacting on NCDs such asthma, chronic obstructive pulmonary disease, diabetes and bowel disease (Gu et al. 2019; Jayasudha et al. 2020; Tiew et al. 2020; van Tilburg Bernardes et al. 2020; Qin et al. 2021). Thus, humans can be viewed as a holobiont, an ecological unit comprised of a host and their associated microbiota in which the resilience of all organisms is interdependent (van de Guchte et al. 2018; Mills et al. 2019). Interactions between the environmental and human microbiome can therefore have a significant impact on public health (Hirt 2020).

Anthropogenic processes, driving changes in land-use (e.g. urbanisation, intensive farming, deforestation and desertification), land-management (e.g. frequent disturbance, increased nutrient input, plant selection and landscape fragmentation) and local environments (e.g. pollution, climatic conditions and nutrient/water availability), can alter the diversity and/or composition of soil microbiota (Wall et al. 2015; Wall and Six 2015; Frąc et al. 2018; Mills et al. 2019). Consequently, soils can become less able to support plant growth, filter and protect water supplies and limit bioaerosol formation (Wall et al. 2015; Frąc et al. 2018; Mills et al. 2019), and humans are exposed to different types and/or levels of soil microbes (Mills et al. 2019). This can reduce food, water and air quality and perturb immune system modulation, respectively, with potential widespread negative impacts on public health (Wall et al. 2015; Wall and Six 2015; Mills et al. 2019). Indeed, modern lifestyles and altered exposure to environmental microbes have been linked to the increased risk of NCDs in residents of industrialised cities across the globe (Ehlers and Kaufmann 2010; Hanski et al. 2012; Lowry et al. 2016; Haahtela 2019; Mills et al. 2019), and populations living in more biodiverse environments with better access to greenspaces have improved health over those in less biodiverse areas (Ruokolainen et al. 2015; Campbell et al. 2017; Tischer et al. 2017; Brindley et al. 2018; Liddicoat et al. 2018; Mavoa et al. 2019; Mills et al. 2019). Consequently, the increased resilience of ecosystems and improved public health provided by greater environmental biodiversity has been recognised by the World Health Organization as an important consideration during rapid global change (WHO 2015).

In an increasingly urbanised world, urban greenspaces offer an opportunity to improve public health, through positive effects on mental, cardiovascular, metabolic, neurophysiological and respiratory health (Barton and Rogerson 2017; Twohig-Bennett and Jones 2018). Many of these benefits are likely, at least in part, to involve microbial-mediated modulation of the immune system (Hanski et al. 2012; Ruokolainen et al. 2015; Lowry et al. 2016; Campbell et al. 2017; Tischer et al. 2017; Liddicoat et al. 2018; Haahtela 2019). As such, there is potential to use microbial inoculants and/or introduce urban planning and land-management strategies to remediate, re-wild and/or provide better health-promoting greenspaces for the improved health of local residents, so-called microbiome-inspired green infrastructure (MIGI) (Robinson et al. 2018; Breed et al. 2019; Watkins et al. 2020).

The first step in the MIGI process is to better understand the microbial components of different types of local urban greenspaces. This is particularly important for the fungal microbiome (mycobiome), which has been much less studied compared to the bacterial microbiome (bacteriome). Moreover, the fungal community appears to be more sensitive to urbanisation and land-use changes than the bacterial community (Epp Schmidt et al. 2017; Delelegn et al. 2018; Wang et al. 2019). Hence, here we analyse the mycobiome of soil samples from five types of urban greenspace in and around Bournemouth by sequencing the variable regions of the internal transcribed spacer (ITS1 and ITS2) and large subunit (LSU) of the fungal rRNA genes using a metabarcoding approach we developed previously (Tonge et al. 2014). Following analysis of two mock communities, we demonstrated that no single region could identify all community species, and so we recommended a multi-region metabarcoding approach should be used where possible. Yet most soil metabarcoding studies focus on a single region, commonly ITS2, with only one advocating the use of multiple regions (ITS1 and SSU) for analysing complex environmental soil samples (George et al. 2019). Thus, our aims were to: (1) assess the added benefits of metabarcoding three regions separately and in combination across multiple taxonomic ranks; (2) examine differences in fungal taxa within the soil from five different types of urban greenspace; and (3) identify those differences of biological relevance to public health.

## Methods

Detailed methods are provided in Supplementary Information 1.

### Sample Collection

Fifteen locally representative public greenspace soil samples were collected on the 4–5 October 2016 based on their current land-use: (1) manicured lawns, (samples 7, 13 and 14); (2) bareground, (samples 8, 10 and 12); (3) parklands with a low-density woody overstory and manicured grassy understory, (samples 4, 11 and 15); (4) young growth forest with weeds and disturbance specialist plants, (samples 2, 6 and 9); and (5) old growth forest, (samples 1, 3 and 5) (Supplementary Information 2). Soil aliquots of 100–200 g were randomly sampled from 9 points within a 25 × 25 m quadrat, pooled and homogenised in a sterilised container, and roots were removed by hand using sterile gloves. Of these pooled samples, 50 g subsamples were frozen until DNA extraction.

### High Throughput Sequencing

#### Mock Community

The eight species mock community developed for our previous work (Tonge et al. 2014) was used as a quality control. Full details of the eight species are shown in Supplementary Information 3.

#### DNA Extraction

DNA was extracted using the PowerSoil™ DNA isolation kit (Mo Bio Laboratories, now a Qiagen company), quantified with a mySPEC microvolume spectrophotometer (VWR) and quality checked using a 2200 TapeStation System (Agilent Technologies). Empty tubes were included as DNA extraction controls.

#### PCR Amplification

Three regions of the fungal rDNA genes (ITS1, ITS2 (White et al. 1990; Op De Beeck et al. 2014)) and the D1/D2 region of LSU (Issakainen et al. 1999)) were amplified for sequencing with Phusion High-Fidelity PCR Master Mix (ThermoFisher Scientific) and the fusion primers detailed in (Tonge et al. 2014) and Supplementary Information 4. The resulting PCR amplicons were purified using AMPure XP beads (Beckman Coulter Life Sciences) and analysed using a DNA 1000 chip on a 2100 Bioanalyzer (Agilent Technologies) to determine concentration and size. Mock community, DNA extraction and non-template controls were included, the latter two demonstrating no amplification.

#### Sequencing

PCR amplicons were diluted to 100 pM, and equal volumes of the amplicons from up to 40 samples were pooled per sequencing run. This resulted in three sequencing runs, each with its own mock community control. Emulsion PCR of the pooled PCR amplicons was performed on the Ion OneTouch™ 2 using the Ion 520™ Kit-OT2 (Ion Torrent™, ThermoFisher Scientific) to produce libraries of up to 400 base-reads. Libraries were sequenced on an Ion S5™ instrument using an Ion 520™ Chip Kit (Ion Torrent™, ThermoFisher Scientific).

#### Bioinformatic Analysis

Fastq files for each sample/region generated by the Torrent Suite™ software (v 5.8.0, ThermoFisher Scientific) under default settings were imported into CLC Genomics Workbench 12 (v 12.0.2, Qiagen). Forward and reverse primers were removed, and full length reads were exported as fastq files and uploaded into Galaxy (version 19.09, (Afgan et al. 2018)). The quality of each file was read using FastQC, and poor quality bases were removed using Trim sequences. All quality checked and trimmed sequences were exported as fastq files and processed in the Cygwin64 terminal using the UPARSE pipeline within USEARCH (v 11.0.667_win32, Edgar 2013, 2016, 2018).

Briefly, reads for each region were truncated (fastx_truncate) so that all sample sequences within each region were the same size (140 bp for ITS1, 210 bp for ITS2 and 320 bp for LSU). Truncated sample files were pooled according to region to produce three files containing mock communities plus fifteen samples. The three region files were dereplicated (fastx_uniques) with a minuniquesize > 10, as recommended for Ion Torrent data (Edgar 2017). Sequences were clustered into operational taxonomic units (OTUs) (cluster_otus), and the original pooled sample reads for each region were mapped to the corresponding OTU file (otutab), again as recommended for Ion Torrent data (Edgar 2019). Octave plots were generated (otutab_octave) to visualise the OTU abundance distribution (diversity) of each sample (Edgar and Flyvbjerg 2018). OTUs with a frequency < 0.5% across all samples were removed from the resulting tables (otu_trim), and OTU tables were rarefied (otutab_rare) to the same number of total reads (86 K for ITS1, 116 K for ITS2 and 58 K for LSU). Taxonomy was assigned (sintax) using the UNITE fungal database (v 8.0) and BLAST+ version 2.9.0+ (with taxdb database downloaded 02/12/19).

Statistical analysis of differences between the greenspaces was performed using the *vegan* (v 2.5.6), *lmPerm* (v 2.1.0) and *indicspecies* (v 1.7.9) packages in R (v 4.0.2). The richness (*specnumber*) and diversity (*diversity*) of each greenspace at all taxa levels were measured in *vegan* (Oksanen et al. 2013). Diversity was estimated as the effective numbers of taxa using both the Shannon and Simpson indices and expressed as e^Shannon^ and 1/(1-Simpson) to reflect the true diversity of the fungal community (Jost 2006; Oksanen et al. 2013; Yan et al. 2018). Differences in richness and diversity across the greenspaces at each taxa level were tested with a multifactor permuted analysis of variance (PERMANOVA) (*aovp* with 5000 permutations) in *lmPerm* (Wheeler et al. 2016; Yan et al. 2018). Differences in community composition (rarefied abundances of fungal taxa) across the greenspaces at each taxa level were tested (*anosim* with 999 permutations and Bray–Curtis dissimilarity matrices, and *adonis* with 999 permutations and Bray–Curtis dissimilarity matrices followed by *anova* for homogeneity of variance) in *vegan* (Oksanen et al. 2013; Yan et al. 2018). The effect of greenspace type on fungal community composition was visualised at each taxa level with non-metric multidimensional scaling (NMDS) (*metaMDS* with Bray–Curtis dissimilarity matrices, *stressplot* and *ordihull*) in *vegan* (Oksanen et al. 2013). The abundance of the different phyla and classes was plotted as percentage of total rarefied reads in GraphPad Prism (v 8.2.1). Since the number of taxa increases with taxonomic rank, those orders, families and genera best separating the different greenspace soils were identified and their abundances plotted on heatmaps as Log_2_[Rarefied reads] in Qlucore Omics Explorer (v 3.5). Differences in specific taxa across the greenspaces were tested with a PERMANOVA (*aovp* with 5000 permutations) in *lmPerm* (Wheeler et al. 2016; Yan et al. 2018), and specific taxa associated with particular greenspaces were determined (*multipatt* function with 9999 permutations) in *indicspecies* (De Caceres et al. 2016)*.* Finally, ecological guilds were assigned to fungal taxa using FUNGuild (v 1.1) (Nguyen et al. 2016), and differences between greenspaces were tested with a PERMANOVA (*aovp* with 5000 permutations) in *lmPerm* (Wheeler et al. 2016; Yan et al. 2018), and specific guilds associated with particular greenspaces were determined (*multipatt* with 9999 permutations) in *indicspecies* (De Caceres et al. 2016).

## Results

A total of 18,232,409 raw reads were generated across three sequencing runs (ITS1: 5,147,519; ITS2: 8,596,064; LSU: 4,888,826), producing 13,371,144 truncated reads for downstream processing (ITS1: 3,827,388; ITS2: 6,916,959; LSU: 2,626,797) (Supplementary Information 5). These reads were assigned to 6,900 OTUs (ITS1: 2,971; ITS2: 2,883; LSU: 1,046), covering an average of 93.5% of the pooled truncated reads (ITS1: 95.9%; ITS2: 95.1%; LSU: 89.6%). Octave plots validated the discarding of OTUs with a size < 10 sequences and demonstrated that more than half or complete OTU diversity was captured in 61–89% of the samples (ITS1: 61%; ITS2: 89%; LSU: 67%) (Supplementary Information 6). After removal of OTUs with a frequency < 0.5% across all samples, a total of 869 OTUs remained (ITS1: 283; ITS2: 314; LSU: 272), covering an average of 80.3% of the pooled truncated reads (ITS1: 81.8%; ITS2: 80.5%; LSU: 78.7%). Between 66.9% and 97.8% of these 869 OTUs were assigned to fungal taxa (Table 1). These OTUs represented up to 3 phyla, 11 classes, 36 orders, 89 families and 124 genera of fungi across the three regions (Table 2). ITS2 and LSU identified a greater number of fungal taxa than ITS1, despite the LSU region generating the least number of OTUs. Combining the results of all three regions improved fungal identification, increasing the number of fungal phyla, classes, orders, families and genera to 4, 13, 43, 121 and 197, respectively.

**Table 1:** Percentage of OTUs with a Frequency > 0.5% Assigned to Fungal Taxa Across the Different Regions.

Region	Phylum	Class	Order	Family	Genus
ITS1	89.8	84.5	80.6	73.9	71.0
ITS2	97.8	93.6	89.2	72.9	68.2
LSU	97.8	89.0	82.0	70.6	66.9
Combined	95.2	89.2	84.1	72.5	68.7

The three regions are shown separately and combined. Regions were analysed using USEARCH and Blastn as described in the methods.

**Table 2:** Number of Fungal Taxa Assigned to OTUs with a Frequency > 0.5% Across the Different regions.

Region	Phylum	Class	Order	Family	Genus
ITS1	3	10	27	61	94
ITS2	3	10	36	82	115
LSU	3	11	34	89	124
Combined	4	13	43	121	197

The three regions are shown separately and combined. Regions were analysed using USEARCH and Blastn as described in the methods.

### Mock Community

Analysis of the mock community (1) validated the bioinformatic pipeline, (2) highlighted the advantage of pooling sample reads prior to dereplication to enhance the signal of low abundant sequences and increase the detection of real biological OTUs over background noise, (3) demonstrated the use of a mock community as a quality control for each sequencing run, (4) established the added value of a multi-region approach and (5) emphasised that relative OTU abundance does not reliably reflect true species abundance and so should only be compared between and not within samples. A detailed description of this analysis is provided in Supplementary Information 7.

### Soil Samples

**Figure 1:**
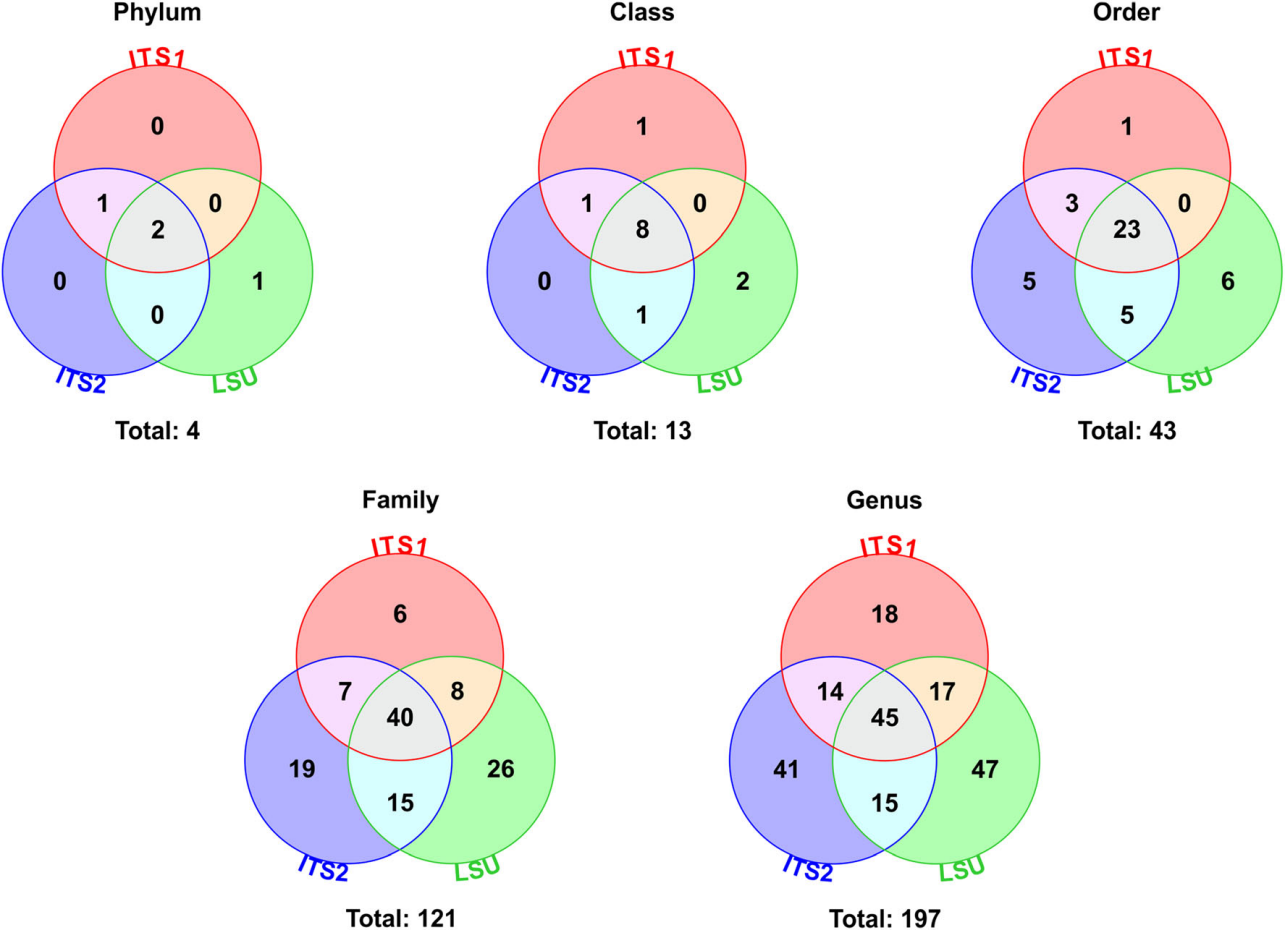
Number of fungal taxa identified by the ITS1, ITS2 and LSU regions across taxonomic ranks. Regions were analysed using USEARCH and Blastn as detailed in the methods. Briefly, raw sequencing reads were truncated, pooled (along with mock community samples), dereplicated and clustered into operational taxonomic units (OTUs) with a size  ≥ 10 reads. All of the original truncated sequences were mapped to these OTUs. OTUs with a frequency < 0.5% across all samples were removed, and OTU tables were normalised (each sample rarefied to same number of reads as the sample with the minimum number). OTUs were assigned to fungal taxa, and numbers identified by single and multiple regions were counted.

Results were largely comparable across the three regions and taxonomic ranks, with many fungal orders, families and genera being identified by multiple regions (Fig. 1). However, each region also identified some additional fungal taxa that were unique to the specific region. The LSU and ITS2 regions identified a greater number of additional fungal taxa compared to ITS1, with LSU identifying the most additional taxa despite generating the least number of OTUs. The combined data therefore provided the most comprehensive analysis and were used for overall data analysis and interpretation. Data for the individual regions are shown in Supplementary Information 8, and the full normalised reads for each taxonomic rank across all samples for the individual and combined regions can be found in Supplementary Information 9.

**Figure 2:**
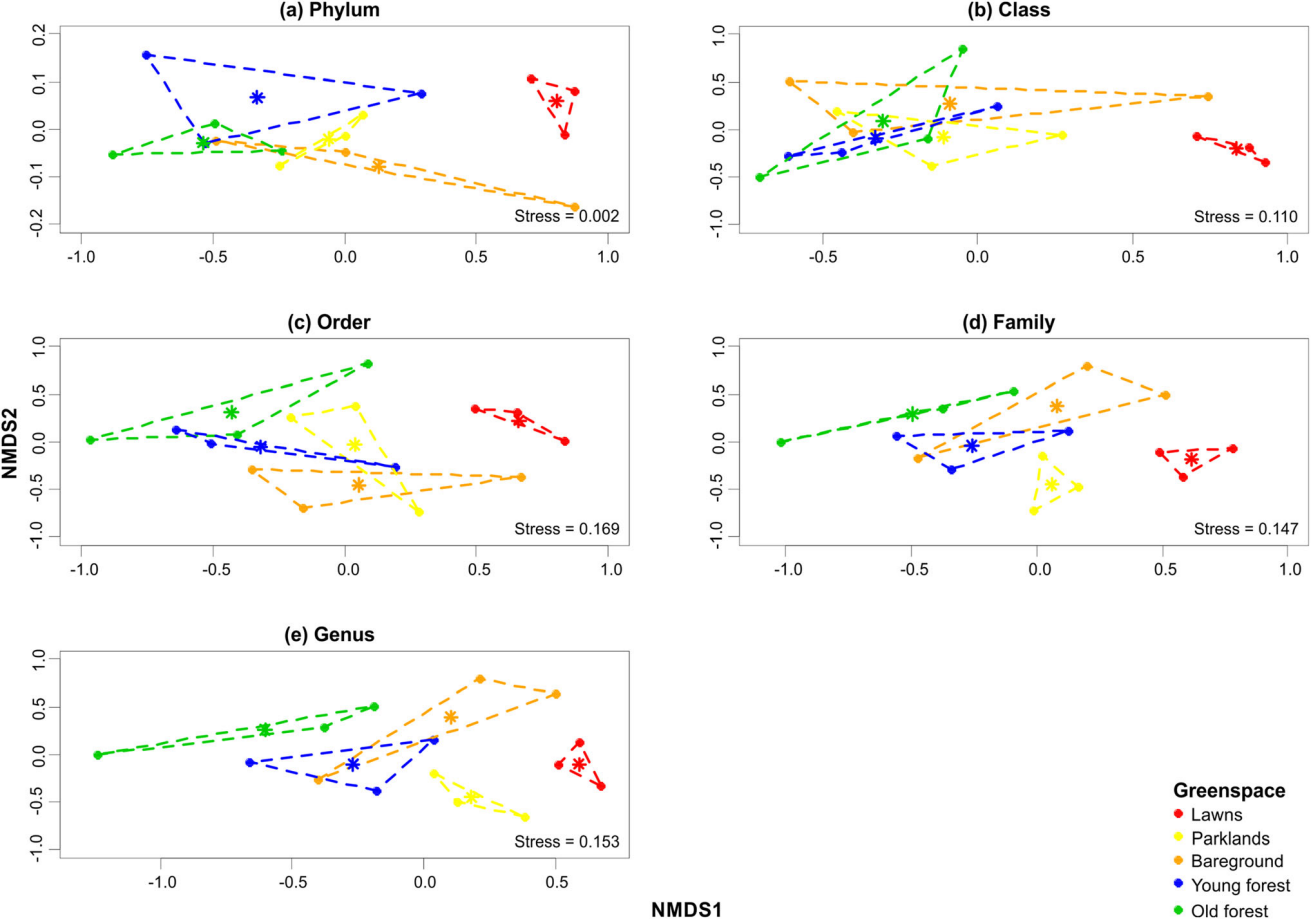
Non-metric multidimensional scaling (NMDS) plots of the fungal community composition at the **a** phylum, **b** class, **c** order, **d** family and **e** genus taxonomic ranks within soils from five urban greenspaces identified by a combination of the ITS1, ITS2 and LSU regions. Regions were analysed individually using USEARCH and Blastn, results were combined, and statistical analysis were performed in R as detailed in the methods. Briefly, raw sequencing reads were truncated, pooled (along with mock community samples), dereplicated and clustered into operational taxonomic units (OTUs) with a size ≥ 10 reads. All of the original truncated sequences were mapped to these OTUs. OTUs with a frequency < 0.5% across all samples were removed, and OTU tables were normalised (each sample rarefied to same number of reads as the sample with the minimum number). OTUs were assigned to fungal taxa, and the effect of greenspace type on fungal community composition was visualised with NMDS.

**Figure 3:**
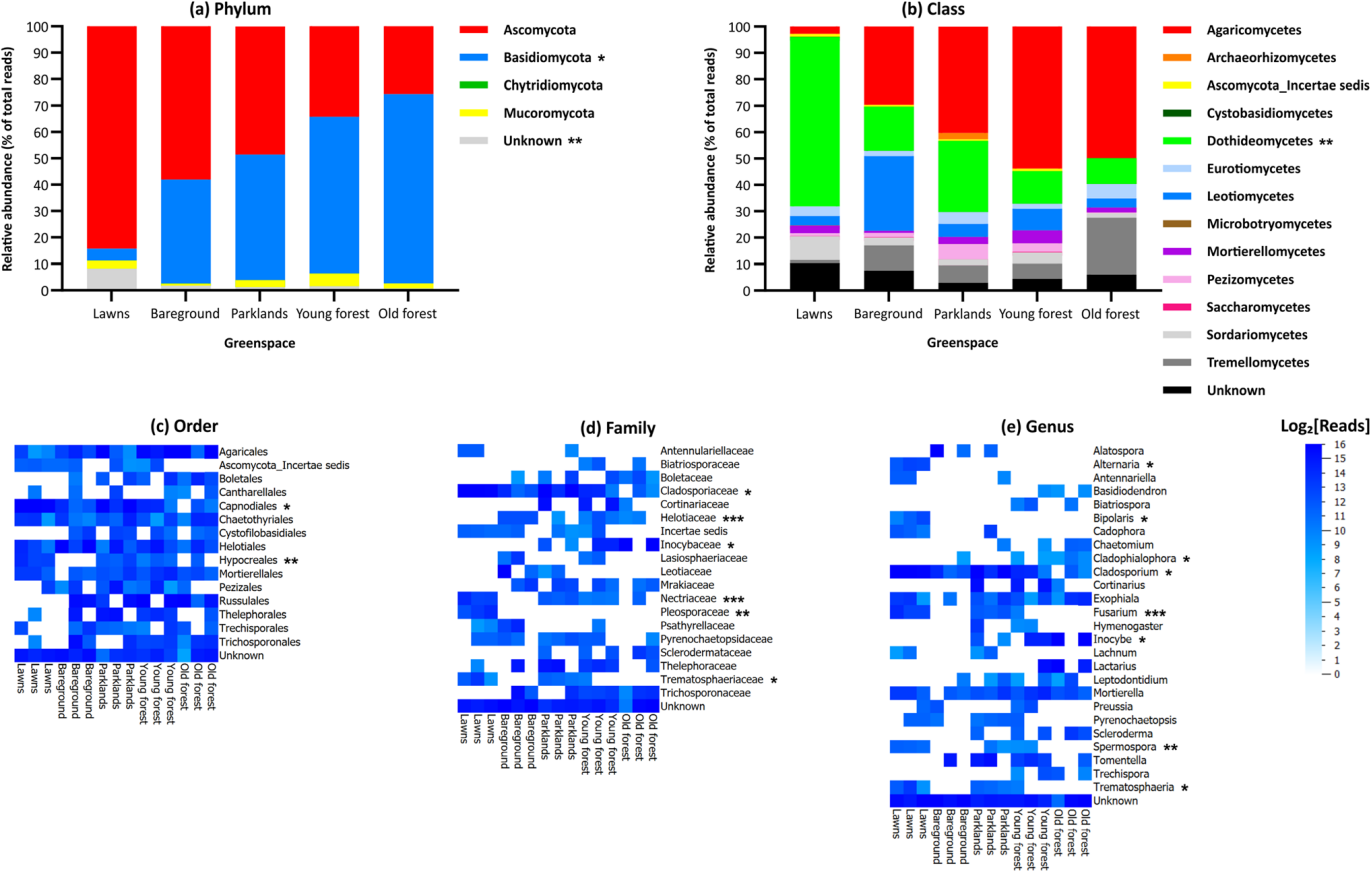
Fungal community composition at the **a** phylum, **b** class, **c** order, **d** family and **e** genus taxonomic ranks within soils from five urban greenspaces identified by a combination of the ITS1, ITS2 and LSU regions. Regions were analysed individually using USEARCH and Blastn, results were combined, and statistical analysis were performed in R and Qlucore Omics Explorer as detailed in the methods. Briefly, raw sequencing reads were truncated, pooled (along with mock community samples), dereplicated and clustered into operational taxonomic units (OTUs) with a size ≥ 10 reads. All of the original truncated sequences were mapped to these OTUs. OTUs with a frequency < 0.5% across all samples were removed, and OTU tables were normalised (each sample rarefied to same number of reads as the sample with the minimum number). OTUs were assigned to fungal taxa, and the abundance of the different phyla and classes was plotted as percentage of total rarefied reads. Since the number of taxa increases with taxonomic rank, those orders, families and genera best separating the different greenspace soils were identified and plotted as Log_2_[Rarefied reads]. Differences in specific taxa across the greenspaces were identified with a PERMANOVA test (*p < 0.05, **p < 0.01 and ***p < 0.001).

There were no statistically significant differences in fungal richness or diversity between the soils from the five urban greenspaces at any taxonomic rank (Table 3). However, the community composition (abundances of fungal taxa) within the five soil types varied significantly across all taxonomic ranks (Table 4), with the biggest differences between the soil fungi from lawns *vs* old forest (Fig. 2). Soil fungi from young forest growth tended to be more similar to those from old forest, while soil fungi from parklands and bareground tended to cluster between those from forest and lawns. It was possible to identify the likely taxa driving these differences (Fig. 3). Although the predominant phyla within all the soil samples were Ascomycota and Basidiomycota, there was a decrease in Ascomycota and an increase in Basidiomycota in the soils from lawns, through bareground, parklands and young forest to old forest (Fig. 3a). The differences in Ascomycota and Basidiomycota across the greenspace soils were bordering on statistical significance (*p* = 0.056) and statistically significant (*p* = 0.024), respectively, and there was a statistically significant difference in unknown phyla between the five soil types (*p* = 0.006). The increased association of Basidiomycota with bareground, parkland, young forest and old forest soils compared to lawn soils boarded on statistical significance (*p* = 0.063), and there was a significant increased association of unknown phyla with the lawn soils *vs* the other four soil types (*p* = 0.013). The predominant classes across the greenspace soils were Agaricomycetes (parklands, young forest and old forest), Dothideomycetes (lawns) and Leotiomycetes (bareground) (Fig. 3b). There was a statistically significant difference in Dothideomycetes (*p* = 0.008), and a difference in Leotiomycetes that boarded on significance (*p* = 0.063), between the five soil types. The increased associations of Dothideomycetes with the soils from lawns and Leotiomycetes with the soils from bareground compared to those from the other greenspaces were statistically significant (*p* = 0.012) and bordering on statistical significance (*p* = 0.069), respectively. Since the number of taxa increases with taxonomic rank, only those orders, families and genera best separating the different greenspace soils are shown (Fig. 3c–e). Of these, there were statistically significant differences in 2 orders (Capnodiales (*p* = 0.013) and Hypocreales (*p* = 0.004)); 6 families (Cladosporiaceae (*p* = 0.019), Helotiaceae (*p* = 0.001), Inocybaceae (*p* = 0.034), Nectriaceae (*p* < 0.001), Pleosporaceae (*p* = 0.010) and Trematosphaeriaceae (*p* = 0.046)); and 8 genera (*Alternaria* (*p* = 0.012), *Bipolaris* (*p* = 0.011), *Cladophialophora* (*p* = 0.019), *Cladosporium* (*p* = 0.013), *Fusarium* (*p* < 0.001), *Inocybe* (*p* = 0.040), *Spermospora* (*p* = 0.004) and *Trematosphaeria* (*p* = 0.048)) across the five soil types. The Hypocreales order was significantly associated with lawn soils (*p* = 0.034), and the Capnodiales order with lawn and parkland soils (*p* = 0.029). The Pleosporaceae and Nectriaceae families were significantly associated with the soils from lawns (*p* = 0.011), the Cladosporiaceae family with those from lawns and parklands (*p* = 0.027), and the Helotriaceae family with those from bareground and young forests (*p* = 0.001). The Inocybaceae family was non-significantly associated with old forest soils (*p* = 0.141) and Trematosphaeriaceae family with lawn and parkland soils (*p* = 0.093). The *Alternaria*, *Spermospora*, *Fusarium* and *Bipolaris* genera were significantly associated with lawn soils (*p* = 0.011), the *Cladosporium* genus with lawn and parkland soils (*p* = 0.028), and the *Cladophialophora* genus with old forest soils (*p* = 0.043). The *Inocybe* genus was non-significantly associated with soils from old forest and the *Trematosphaeria* genus with those from lawns and parklands (*p* = 0.087).

**Table 3:** Analysis of Richness and Diversity Across Fungal Taxa Within Soils from Five Urban Greenspaces Identified by a Combination of the ITS1, ITS2 and LSU Regions.

	Phylum			Class			Order		
Greenspace	Richness^a^	Diversity^b^		Richness^a^	Diversity^b^		Richness^a^	Diversity^b^	
		Shannon	Simpson		Shannon	Simpson		Shannon	Simpson
Lawns	4 ± 0	1.8 ± 0.3	1.4 ± 0.2	10 ± 2	3.6 ± 0.8	2.3 ± 0.5	16 ± 2	5.1 ± 0.7	3.2 ± 0.8
Parklands	4 ± 1	2.3 ± 0.2	2.1 ± 0.1	10 ± 1	4.9 ± 1.8	3.7 ± 1.3	19 ± 3	9.5 ± 2.7	6.7 ± 2.4
Bareground	4 ± 0	1.9 ± 0.6	1.7 ± 0.5	9 ± 2	4.1 ± 0.9	3.1 ± 0.4	16 ± 7	7.7 ± 4.2	5.6 ± 3.1
Young forest	4 ± 0	2.2 ± 0.2	1.8 ± 0.2	10 ± 1	4.8 ± 2.6	3.6 ± 2.6	22 ± 2	10.6 ± 3.8	7.5 ± 4.1
Old forest	4 ± 1	1.9 ± 0.4	1.7 ± 0.4	18 ± 1	3.7 ± 1.7	2.7 ± 1.2	18 ± 3	7.8 ± 3.8	5.3 ± 2.7
PERMANOVA^c^	1.00	0.41	0.19	0.21	0.84	0.78	0.40	0.33	0.45

	Family			Genus		
Greenspace	Richness^a^	Diversity^b^		Richness^a^	Diversity^b^	
		Shannon	Simpson		Shannon	Simpson
Lawns	24 ± 5	7.1 ± 2.7	4.2 ± 2.2	27 ± 6	7.4 ± 3.4	4.2 ± 2.4
Parklands	28 ± 5	11.6 ± 2.6	7.5 ± 2.1	31 ± 8	12.9 ± 3.9	8.2 ± 3.0
Bareground	24 ± 7	9.7 ± 3.1	6.2 ± 2.4	29 ± 6	9.5 ± 3.9	5.6 ± 2.8
Young forest	35 ± 8	16.4 ± 7.4	10.6 ± 6.1	39 ± 10	17.3 ± 7.8	11.2 ± 5.7
Old forest	23 ± 3	7.5 ± 1.9	5.0 ± 1.2	25 ± 3	8.1 ± 1.1	5.4 ± 0.7
PERMANOVA^c^	0.15	0.06	0.18	0.17	0.09	0.13

Regions were analysed individually using USEARCH and Blastn and results were combined, followed by statistical analysis using the *vegan* and *lmPerm* packages in R as detailed in the methods.

^a^Taxa number as determined by the *specnumber* function in *vegan*.

^b^Effective numbers of taxa as determined by the *diversity* function using the Shannon and Simpson indices in *vegan* and expressed as e^Shannon^ and 1/(1-Simpson).

^c^Multifactor permuted analysis of variance (PERMANOVA) *p* value as determined by the *aovp* function in *lmPerm*.

**Table 4:** Analysis of Differences in Community Composition Across Fungal Taxa Within Soils from Five Urban Greenspaces Identified by a Combination of the ITS1, ITS2 and LSU Regions.

Taxa	ANOSIM^a^		ADONIS^b^		Homogeneity of variance (ADONIS)^c^		
	R statistic	Significance	R statistic	Significance	F value	Significance	Homogeneous
Phylum	0.332	0.035	0.631	0.038	0.902	0.499	Y
Class	0.296	0.033	0.485	0.035	0.527	0.719	Y
Order	0.322	0.022	0.420	0.027	0.237	0.911	Y
Family	0.433	0.007	0.448	0.003	0.282	0.883	Y
Genus	0.449	0.003	0.448	0.001	0.368	0.826	Y

Regions were analysed individually using USEARCH and Blastn, and results were combined, followed by statistical analysis using the *vegan* package in R as detailed in the methods.

^a^Analysis of similarities (ANOSIM) as determined by the *anosim* function in *vegan*.

^b^Multifactor permuted analysis of variance (PERMANOVA) as determined by the *adonis* function in *vegan.*

^c^Confirmation of variance homogeneity for ADONIS test to support the conclusion that the statistically significant differences across taxa are due to type of greenspace.

**Figure 4:**
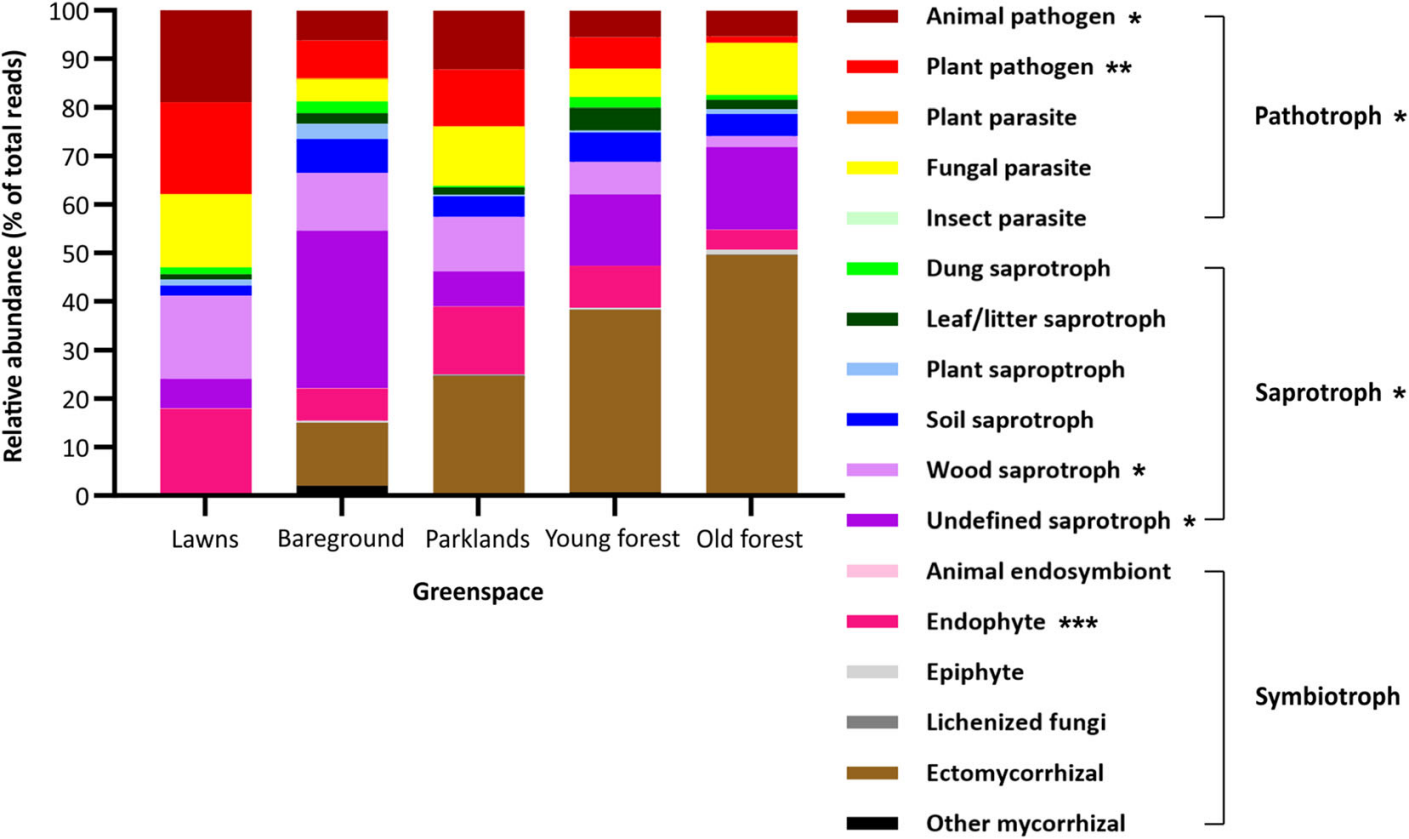
Fungal guilds within five urban greenspaces identified by the ITS1, ITS2 and LSU regions. Regions were analysed individually using USEARCH and Blastn, results were combined prior to processing in FUNGuild, and statistical analysis were performed in R as detailed in the methods. Briefly, raw sequencing reads were truncated, pooled (along with mock community samples), dereplicated and clustered into operational taxonomic units (OTUs) with a size ≥ 10 reads. All of the original truncated sequences were mapped to these OTUs. OTUs with a frequency < 0.5% across all samples were removed, and OTU tables were normalised (each sample rarefied to same number of reads as the sample with the minimum number). OTUs were assigned to fungal taxa and then to ecological guilds, and the abundance of the different guilds was plotted as percentage of total rarefied reads. Differences in specific guilds across the greenspaces were identified with a PERMANOVA test (*p < 0.05, **p < 0.01 and ***p < 0.001).

To explore the potential functional consequences of these differences, fungal taxa were assigned into ecological guilds, with 95% being successfully assigned (Fig. 4). While there were no statistically significant differences in the richness or diversity of these guilds across the greenspace soils (*p* ≥ 0.168), the guild composition varied significantly between the five soil types (*p* < 0.013). Ectomycorrhizal fungi (EMF) were present at low levels (0.3% relative abundance) in lawn soils, increased through bareground, parkland and young forest to old forest soils. Despite neither the differences in EMF across the greenspaces nor the association of EMF with a particular soil type reaching statistical significance (*p* = 0.146 and *p* = 0.189, respectively), the trend of increased EMF within the soils from forests *vs* lawns was likely driven by the Agaricales, Boletales, Cantharellales, Russulales and Thelephorales orders (Fig. 3c); the Boletaceae, Cortinariaceae, Inocybaceae, Sclerodermataceae and Thelephoraceae families (Fig. 3d); and the *Cortinarius*, *Hymenogaster*, *Inocybe*, *Lactarius*, *Scleroderma* and *Tomentella* genera (Fig. 3e). These ectomycorrhizal fungal taxa were either absent from lawn soils and/or present at greater levels in forest soils. Soils from lawns did contain some other beneficial fungal taxa, such as the endophytic *Cadophora* and *Mortierella* genera. Endophytes were significantly different across the five soil types (*p* = 0.001) and significantly associated with soils from lawns compared to the those from the other greenspaces (*p* = 0.020).

There were significant differences in saprotrophs in general (*p* = 0.049) and in both wood (*p* = 0.025) and undefined (*p* = 0.045) saprotrophs between the five soil types. Saprotrophs were significantly associated with soils from bareground *vs* those from the other greenspaces (*p* = 0.049), with the majority classified as undefined saprotrophs. Distinct fungal taxa within the different bareground samples were responsible for the increase in undefined saprotrophs (Supplementary Information 9). Most of the undefined saprotrophs in samples 8 and 12 were from the Helotiales order with some contribution from the Agaricales order, while those in sample 10 were predominantly from the Agaricales order with some contribution from the Helotiales and Trechisporales orders. Thus, the Helotiaceae and Leotiaceae families dominated the undefined saprotrophs in samples 8 and 12, while those from the Lasiosphaeriaceae and Psathyrellaceae families dominated sample 10. Greater variation was observed in genera between the three bareground samples with *Alatospora*, *Coprinellus*, *Murispora* and *Cryptosporiopsis* accounting for the increased undefined saprotrophs in sample 8, *Parasola*, *Coprinopsis*, *Apodus* and *Bloxamia* in sample 10, and *Hyaloscypha*, *Boidinia* and *Stypella* in sample 12.

There were significant differences in pathotrophs in general (*p* = 0.029) and in both animal (*p* = 0.012) and plant (*p* = 0.009) pathogens across the greenspace soils. Pathotrophs were significantly associated with lawn and parkland soils compared to the other four soil types (*p* = 0.023). In particular, animal (*p* = 0.014) and plant (*p* = 0.035) pathogens were significantly associated with soils from lawns and parklands. This was largely driven by *Alternaria*, *Bipolaris*, *Cladosporium*, *Fusarium* and *Spermospora* (Fig. 3e). As reported above, these 5 genera were significantly associated with either lawn soils (*p* = 0.011) or lawn and parkland soils (*p* = 0.028).

## Discussion

Soil microbes play an important role in public health, both indirectly by influencing food, water and air quantity and/or quality and directly via modulation of the human microbiome and immune system (Wall et al. 2015; Wall and Six 2015; Frąc et al. 2018; Mills et al. 2019; Hirt 2020). In an increasingly urbanised world, greenspaces become an important route of exposure to such environmental microbes. A better understanding of the microbiome within different urban greenspaces offers the opportunity to maximise positive and minimise negative exposures to promote improved public health (Robinson et al. 2018; Breed et al. 2019; Mills et al. 2019). Here, we present the fungal analysis of fifteen samples from five different urban greenspaces in and around Bournemouth using a combined multi-region metabarcoding approach.

### Benefits of a Multi-Region Approach

While the individual regions generated broadly similar results across multiple taxonomic ranks from phylum to genus, each region detected additional fungal taxa that were unique to the individual region. The ITS regions assigned a greater percentage of OTUs to fungal taxa than the LSU region, possibly because ITS1 and 2 have been sequenced more than LSU and so more ITS sequence data are available in the reference databases. However, the LSU region identified the greatest number of additional fungal taxa and was the only region following frequency trimming and rarefaction that distinguished all 8 species from the mock community samples, doing so with a reduced number of reads. That different regions share commonalities and differences in detecting fungal taxa is well known and emphasises how each region is inherently biased towards the detection of certain taxa. Rather than contrasting the data from different regions, or using it to determine the ‘most appropriate’ single region approach, we recommend combining results from the ITS1, ITS2 and LSU regions to provide a more comprehensive analysis across all fungal taxonomic ranks.

This will ensure that metabarcoding studies (1) continue to generate sequencing data across multiple regions to better populate databases and thus improve the assignment of sequences to fungal taxa; and (2) maximise findings from complex environmental samples.

### Differences in Fungal Taxa Between Urban Greenspace Soils

#### Study Limitations

It is important to note that this was a small observational study. Only the minimum number of sample replicates (n = 3) was available, and full diversity was not captured in all samples. In addition, there were a significant number of unassigned reads, the abundance of which was not equal across all samples. Thus, we are unlikely to have captured all relevant fungal taxa. Furthermore, the samples were all from in and around a single city, which limits the applicability of our results, and thus conclusions, to other urban areas and environment types. Fungal communities within soil are highly dependent upon their local environment, in terms of nutrient levels, climate, human activity and plant vegetation. These factors were not measured here as we were interested in the overall effect of urban greenspace on the soil mycobiome, rather than the individual factors driving the effect. Despite all of these limitations, we were able to identify statistically significant differences in fungal community composition of urban greenspace soils that are in keeping with the those from similar environments within natural and agricultural spaces, and of potential relevance to public health.

#### Study Results

Within the five different urban greenspace soils, we identified a large number of fungal taxa common to the UK, many of which are mycorrhizal and/or saprophytic and known to inhabit soil ecosystems. It was the composition of these fungal taxa, rather than overall diversity, that demonstrated statistically significant differences across the five soils types. Similar results in fungal diversity *vs* community composition have also been observed in natural and agricultural environments (Timling et al. 2014; Sepp et al. 2018). These data highlight that although changes in diversity may serve as a useful indicator of altered fungal community composition, changes in community composition can occur in the absence of altered diversity. It is therefore vital to identify the specific fungal taxa driving such changes to fully understand their biological relevance.

The predominance of Basidiomycota in the soils from urban forests *vs* Ascomycota in those from urban lawns in and around Bournemouth is in agreement with the wider literature (Tedersoo et al. 2014; Detheridge et al. 2018; Canini et al. 2019; Wang et al. 2019) and likely reflects differing nutrient sources. Low quality and woody substrates common in forest soils select for those fungi capable of extracting/cycling more recalcitrant carbon sources (mainly Basidiomycota, particularly the Agaricomycetes class). In contrast, grassland sources favour those that thrive on more readily available or simple nutrients (mainly Ascomycota) (Detheridge et al. 2018; Canini et al. 2019; Wang et al. 2019). This may also explain the increase in Agaricomycetes and EMF within the Bournemouth forest *vs* lawn soils, since many of the EMF in the Bournemouth samples were from the Agaricomycetes class. Reduced levels of EMF in soils from grassland, agricultural or industrial sites compared to those from forests have been documented at many sites around the world (Midgley et al. 2007; Tedersoo et al. 2014; Epp Schmidt et al. 2017; Detheridge et al. 2018; Wang et al. 2019). This has been linked to increased nitrogen content of fertilised soils, removal of vegetation (particularly woody material) and greater disturbance through mowing, grazing and tillage (Tedersoo et al. 2014; Epp Schmidt et al. 2017; Hui et al. 2017; Delelegn et al. 2018; Canini et al. 2019). Such factors may have contributed to the lack of EMF in the soils from Bournemouth lawns, which are likely to be subjected to greater footfall, and management processes such as fertilisation, mowing and weeding.

Distinct fungal taxa, predominantly from the two most abundant bareground soil classes (Leotiomycetes and Agaricomycetes), were responsible for the increase in undefined saprotrophs within the bareground *vs* other Bournemouth greenspace soils. The most abundant undefined saprotroph(s) in the bareground soil close to the River Stour was the aquatic *Alatospora*; near a golf course surrounded by trees were the short grass/lawn-associated *Parasola* and the wood-associated *Coprinopsis*; and within a children’s play park surrounded by trees were the wood-associated *Boidinia* and *Hyaloscypha*. This is in keeping with local environmental conditions having a large effect on the fungal composition of soils and suggests bareground soils favour saprophytic fungi capable of opportunistic survival on the available local non-living organic material.

Of potential concern was the general increase in pathogenic fungi within the soils from Bournemouth lawns and parklands *vs* forests. This was largely driven by the plant pathogens *Alternaria*, *Bipolaris*, *Cladosporium*, *Fusarium* and *Spermospora*, most of which are also common human allergens and have been associated with infection (Chowdhary et al. 2014; Nucci et al. 2015; Rick et al. 2016; Barnes 2019). Reduced competition with, and/or inhibition by, mycorrhizal fungi combined with more available nutrients may promote the increased growth of pathogenic soil fungi (Wilberforce et al. 2003; Wall et al. 2015; Detheridge et al. 2016; Frąc et al. 2018; Mills et al. 2019). Different anthropogenic practices in Bournemouth for lawns and parklands *vs* forests, such as greater disturbance and use of fertilisers, may therefore contribute to this increase in fungal pathogens in the soils from lawns and parklands. Consequently, local Bournemouth residents frequenting urban lawns and parks may be exposed to higher levels of soil pathogens, potentially impacting their health.

Whether the effects of urban greenspaces on the fungal composition of their associated soils as reported here extend beyond Bournemouth to other towns and cities or urban environments requires further investigation. In support of some commonality is the fact that the results from the Bournemouth study share similarities with studies on the effects of land-use and land-management on soil fungal communities within natural and agricultural environments. Thus, although this was a small study with numerous limitations, we can combine the results with the wider literature to suggest some potential improvements to optimise urban greenspaces in general.

### Potential Improvements to Maximise the Benefit of Urban Greenspaces

A major impact of urban lawns worldwide is the loss of EMF. Changes to both residential and community lawns that could promote the growth of mycorrhizal communities within urban grasslands include: (1) reducing human activities such as mowing and fertilisation; (2) allowing mown grass litter or leaf litter from nearby vegetation to provide more natural nutrients; (3) introducing trees and/or areas of additional vegetation such as urban meadows; and (4) adding mycorrhizal inoculants (Al-Karaki and Othman, 2007; Epp Schmidt et al. 2017; Hui et al. 2017; Frąc et al. 2018; Norton et al. 2019).

Increasing mycorrhizal fungi in the soils from both lawns and parklands may also help to outcompete and/or directly inhibit pathogenic soil fungi (Wilberforce et al. 2003; Detheridge et al. 2016; Frąc et al. 2018). It is worth noting that the soils from Bournemouth lawns did contain some beneficial fungi, including the endophyte *Cadophora* and the saprotroph *Lachnum*. Both *Cadophora* and *Lachnum* have been shown to promote plant growth (Bizabani and Dames, 2015; Berthelot et al. 2016), and *Cadophora* has been shown to inhibit colonisation of grass roots by Fusarium (Wilberforce et al. 2003; Detheridge et al. 2016). Supporting the growth of beneficial fungi may therefore improve the health of the urban ecosystem while simultaneously reducing the exposure of local residents to more harmful fungal taxa.

Maximising the use of bareground sites should also be considered. In keeping with bareground soils from natural environments (Timling et al. 2014; Canini et al. 2019), the Bournemouth bareground soils were rich in saprophytic and mycorrhizal fungi. These fungi may not be actively growing, since metabarcoding does not distinguish between viable and non-viable taxa. However, they may represent dormant spores that could act as an inoculum to support vegetated growth under favourable environmental conditions (Timling et al. 2014). It may therefore be more beneficial to encourage diverse plant growth with minimal disturbance on urban bareground sites rather than converting them to lawns.

For such urban land-use and land-management changes to be developed and actioned, more research is required. This should include characterisation of microbes in the air as well as the soil, since (1) inhalation is a major route of exposure to environmental microbes, including those from the soil, and (2) fungal communities in the air have recently been shown to be sensitive to urbanisation, even more so than those in the soil (Abrego et al. 2020). Further studies do not only need to continue to map the environmental microbiome, but also to determine how these microbes interact with the human microbiome and impact health. It is important to understand which, and under what circumstances, microbes are beneficial or harmful. Care must be taken to consider the health consequences of non-native fungi on a native human population that may not have been previously exposed to such fungi (Tischer et al. 2017). We also need to determine the best methods of introducing microbes into the urban environment to achieve sustainable growth and optimise contact with the target population for maximum impact. Finally, it is also important not just to understand these relationships in the context of human health but also with respect to the health of the whole environment (van Heezik and Brymer 2018). Fungal soil communities form complex interactions with other microbial, invertebrate and plant communities. Changes in one can impact on the condition of another. We do not want to introduce new microbes to improve human health at the expense of the health of the wider environment.

## Conclusions

Our combined metabarcoding approach provided a more comprehensive analysis of the soil mycobiomes from five different types of urban greenspace. These results provide further evidence that land-use impacts the environmental mycobiome and highlight the importance of identifying specific changes in fungal taxa (rather than overall diversity or richness) for understanding their biological relevance. Urban lawn soils in Bournemouth were the most different to the other greenspace soils, particularly those from urban forest soils. Of greatest concern for local public health was the significant increase of pathogenic fungi in the soils from urban lawns and parklands *vs* those from urban forests. As the value of greenspace within the urban environment becomes more widely recognised, it is vital that we explore the underlying biological processes linking urban greenspaces and health. The resulting knowledge will ensure that we develop the best urban planning strategies to protect and promote the health of our local urban environments and their residents.

## Supplementary Information

Below is the link to the electronic supplementary material.

Supplementary file1 (DOCX 8121 kb)
