# Supplementary material for: Metabarcoding of Soil Fungi from Different Urban Greenspaces Around Bournemouth in the UK
Source: Ecohealth. 2021 Jun 5;18(3):315–30. doi: 10.1007/s10393-021-01523-1 (PMC8626400; doi:10.1007/s10393-021-01523-1)
Supplement: Supplementary file 1 — Supplementary file1 (DOCX 8121 kb) [file 10393_2021_1523_MOESM1_ESM.docx]

**Metabarcoding of soil fungi from different urban greenspaces around Bournemouth in the UK**

Contents: Pages:

Supplementary Information 1: Detailed methods 2

Supplementary Information 2: Map of sampling sites 5

Supplementary Information 3: Mock community 6

Supplementary Information 4: Primer sequences 7

Supplementary Information 5: Numbers of reads 8

Supplementary Information 6: Octave plots 10

Supplementary Information 7: Detailed mock community analysis 13

Supplementary Information 8: Individual region analysis 18

Supplementary Information 9: Normalised data across taxa and regions 23

**Supplementary Information 1** Detailed background and description of experimental methods

1.1 Background to metabarcoding

Traditional methods of fungal identification have relied upon culture or microscopy. These bias results towards those species that can be cultured and are often unable to distinguish morphologically similar species, inevitably underestimating biodiversity. With the advent of high throughput sequencing, it has become possible to analyse genetic material recovered directly from environmental samples, so-called metagenomics. The amplicon-based approaches (termed metabarcoding) developed for bacterial identification have been modified to enable high-throughput, cost-effective and culture independent identification of fungal species. However, fungal metagenomics can offer some unique challenges: 1) fungal cells and spores are often encased in protective layers that can be more difficult to lyse, so care needs to be taken to ensure maximum nucleic acid extraction; 2) fungal spores can be of different sizes and contain different amounts of DNA, which can impact quantitative comparisons; 3) fungi have not been profiled to the same extent as bacteria, thus fungal analysis software and reference databases are not as well developed or comprehensive as those for bacterial metabarcoding; and 4) different life stages of the same fungi are morphologically different, resulting in multiple names for the same species depending on historical discovery and classification. Nevertheless, in 2014, we described a method using the benchtop ion torrent platform for identifying fungal species within two mock communities by sequencing the variable regions of the internal transcribed spacer (ITS1 and ITS2) and large subunit (LSU) of the rRNA genes [1]. This demonstrated that no single region could identify all species within the mock communities, and so we recommended a multi-region metabarcoding approach should be taken where possible [1]. The ion torrent platform has been successfully used to analyse the mycobiome of different soil types from a range of geographical locations [2-11]. However, most studies sequence a single region, predominantly ITS2, with only two analysing two regions (ITS1 & 2). In addition, while other NGS platforms have also been used to sequence multiple rRNA gene regions (including ITS1, ITS2, LSU and SSU (the small subunit)), studies have been predominantly comparative and/or focussed on clinical isolates, experimental samples, specific cultured genera, phylogenetic classification, method development, single taxonomic ranks, and/or which region gave the best results [12-20]. Only one advocated the use of multiple regions (ITS1 and SSU) for analysing complex environmental soil samples [19].

Here we apply our three-region metabarcoding approach to the Bournemouth soil samples to assess the added benefits of sequencing multiple regions for improved analysis of the mycobiome of different urban greenspaces. Specifically, our aims were to: 1) assess the metabarcoding of three regions separately and in combination across multiple taxonomic ranks; 2) examine differences in fungal taxa within the soil from five different types of urban greenspace; and 3) identify those differences of biological relevance to public and/or environmental health.

1.2 Sample collection

Fifteen soil samples were collected from around Bournemouth, UK at the urban greenspace sites shown in Supplementary Information 2. These locally representative public greenspaces were categorised based on their current land use: (1) manicured lawns, (samples 7, 13 and 14); (2) bareground, (samples 8, 10 and 12); (3) parklands with a low-density woody overstory and manicured grassy understory, (samples 4, 11 and 15); (4) young growth forest with weeds and disturbance specialist plants, (samples 2, 6 and 9); and (5) old growth forest, (samples 1, 3 and 5). On the 4-5 October 2016, the soil in each greenspace was sampled according to the Biomes of Australian Soil Environments (BASE) project protocol [21]. Using a bleached trowel, 100-200 g aliquots of the top 10 cm of soil within a 25 x 25 m quadrat were randomly sampled from 9 points (e.g. under vegetation, bareground) with a bleached trowel. Aliquots were pooled and homogenised in a sterilised container, and roots were removed by hand using sterile gloves. Of these pooled samples, 50 g subsamples were frozen on the day of collection until DNA extraction. This method has been shown to be adequate to describe and capture microbial variation within greenspaces [22].

1.3 High throughput sequencing

1.3.1 Mock community

The eight species mock community developed for our previous work [1] was used. Full details of the eight species, representing eight genera of common fungal spores of varying size and complexity, are shown in Supplementary Information 3. This served as a quality control for each sequencing run.

1.3.2 DNA extraction

DNA from three 250mg subsamples of each soil sample was extracted using the PowerSoil^TM^ DNA isolation kit according to the manufacturer’s instructions, including the optional incubations at 65^o^C and 4^o^C (Mo Bio Laboratories, now a Qiagen company). DNA extraction controls (empty tubes) were included to check for potential contamination during the extraction process. The quantity and quality of DNA from each triplicate was determined using a mySPEC microvolume spectrophotometer (VWR) and a 2200 TapeStation System (Agilent Technologies), respectively. The DNA Integrity numbers of all samples were >6, signifying they had minimal degradation and were suitable for downstream amplicon-based sequencing. The three triplicates for each soil sample were then pooled and the DNA concentration was re-measured. The resulting final set of fifteen DNA samples was stored at -80^o^C until PCR amplification.

1.3.3 PCR amplification

Three regions of the fungal rDNA genes were amplified for sequencing: ITS1, ITS2 [23, 24] and the D1/D2 region of LSU [25]. The fusion primers detailed in [1], which comprised a region specific priming element and the Ion Torrent^TM^ adapters for subsequent sequencing, were modified to include an additional 10 nt Ion Xpress^TM^ barcode. This enabled multiple samples and regions to be sequenced in the same sequencing run. Forward primers for each of the three regions were fused with sequencing adapter A and a barcode, while reverse primers were fused with a truncated version of adapter P1 (Supplementary Information 3). Independent PCR reactions, (containing 10 μl of Phusion High-Fidelity PCR Master Mix (ThermoFisher Scientific), 2 μl of 10μM primer mix, 1-100 ng of DNA and molecular grade water to a final volume of 20 μl), were performed for each of the three regions. Cycling conditions were 98^o^C for 2 min; followed by 30 cycles of 98^o^C for 10 s, 60-68^o^C (66^o^C for ITS1, 60^o^C for ITS2 and 68^o^C for LSU) for 30 s, 72^o^C for 30 s; ending with 72^o^C for 5 min. The volume of each PCR reaction was increased to 25 μl by the addition of 5 μl of molecular grade water. The resulting PCR amplicons were then purified using 45 μl (1.8X volume) of AMPure XP beads (Beckman Coulter Life Sciences) and eluted in 25 μl of Low TE buffer. A 1 μl aliquot of each PCR product was analysed with a DNA 1000 chip on a 2100 Bioanalyzer (Agilent Technologies) to determine concentration and size. Mock community, DNA extraction and non-template controls were included, the latter two demonstrating no amplification. All PCR amplicons were stored at -20^o^C until sequencing.

1.3.4 Sequencing

All PCR amplicons were diluted to 100 pM, and equal volumes of the amplicons from up to 40 samples were pooled per sequencing run. This resulted in the PCR amplicons being sequenced across three runs, each with its own mock community control. Emulsion PCR of the pooled PCR amplicons was performed on the Ion OneTouch^TM^ 2 using the Ion 520^TM^ Kit-OT2 (Ion Torrent^TM^, ThermoFisher Scientific) to produce libraries of up to 400 base-reads according to the manufacturer’s instructions. The resulting libraries were then sequenced on an Ion S5^TM^ instrument using an Ion 520^TM^ Chip Kit (Ion Torrent^TM^, ThermoFisher Scientific) according to the manufacturer’s instructions.

1.3.5 Bioinformatic analysis

Following each sequencing run, the resulting fastq files for each sample/region generated by the Torrent Suite^TM^ software (version 5.8.0, ThermoFisher Scientific) under default settings were imported into CLC Genomics Workbench 12 (version 12.0.2, Qiagen). Forward and reverse primers were removed, and only full length reads were kept. These full length reads were exported as fastq files and uploaded into Galaxy (version 19.09, [26]). Here the quality of each file was read using FastQC and the length at which per base sequence quality began to drop off was noted. The first 9 bases of the LSU sequences from samples 3, 7, 9 and 15, and the first 10 bases of the ITS2 sequences of sample 1 were removed using Trim sequences due to poor per base quality at base 8 and 9, respectively. The quality of these trimmed files was re-checked using FastQC, and all quality checked and trimmed sequences were exported as fastq files. Files were then processed using the UPARSE pipeline within USEARCH (version 11.0.667_win32, [27-29]) in the Cygwin64 terminal.

Reads for each region were truncated to the shortest required size (140 bp for ITS1, 210 bp for ITS2 and 320 bp for LSU) using the fastx_truncate command so that all sample sequences within each region were the same size. The relabel option was used to ensure each read had a unique identifier. Truncated sample files were pooled according to region to produce three files containing mock communities plus fifteen samples. Pooling is recommended in UPARSE following truncation but prior to dereplication so that dereplication reflects the abundances of unique biological sequences across all samples [30]. The three region files were dereplicated using the fastx_uniques command with a minuniquesize > 10. The Q scores for different sequencing platforms are calculated differently, thus Ion Torrent data is not suitable for the quality filtering commands within UPARSE. Instead of quality filtering prior to dereplication, it is recommended to use a minuniquesize > 10 as an alternative [31]. Sequences were then clustered into operational taxonomic units (OTUs) using the cluster_otus command, and the original pooled sample reads for each region were mapped to the corresponding OTU file using the otutab command. This allows the maximum number of reads to be mapped to OTUs. Clustering to generate OTUs is recommended over denoising to form amplicon sequence variants when using Ion Torrent data [32]. Octave plots were generated with the otutab_octave command to visualise the OTU abundance distribution (diversity) of each sample [33]. OTUs with a frequency <0.5% across all samples were removed from the resulting tables using the otu_trim command. OTU tables were then normalised (each sample rarefied to same number of reads as the sample with the minimum number, 86K for ITS1, 116K for ITS2 and 58K for LSU) with the otutab_rare command. Taxonomy was assigned to the OTU table using the sintax command (with UNITE fungal database version 8.0) and BLAST+ version 2.9.0+ (with taxdb database downloaded 02/12/19). Taxonomic results were compared, and final classifications assigned manually.

Statistical analysis of differences between the greenspaces was performed using the *vegan* (version 2.5.6), *lmPerm* (version 2.1.0) and *indicspecies* (version 1.7.9) packages in R (version 4.0.2). The richness and diversity of each greenspace at all taxa levels were measured with the *specnumber* and *diversity* functions in *vegan*, respectively [34]. Diversity was estimated as the effective numbers of taxa using both the Shannon and Simpson indices and expressed as e^Shannon^ and 1/(1-Simpson) to reflect the true diversity of the fungal community [22, 34, 35]. Statistically significant differences in richness and diversity across the greenspaces at each taxa level were tested with a multifactor permuted analysis of variance (PERMANOVA) using the *aovp* function (with 5000 permutations) in *lmPerm* [22, 36]. Statistically significant differences in community composition (rarefied abundances of fungal taxa) across the greenspaces at each taxa level were tested with an analysis of similarities (ANOSIM) using the *anosim* function (with 999 permutations and Bray-Curtis dissimilarity matrices) and a PERMANOVA using the *adonis* function (with 999 permutations and Bray-Curtis dissimilarity matrices) followed by a test for homogeneity of variance using the *anova* function in *vegan* [22, 34]. The effect of greenspace type on fungal community composition was visualised at each taxa level with non-metric multidimensional scaling (NMDS) using the *metaMDS* (with Bray-Curtis dissimilarity matrices), *stressplot* and *ordihull* functions in *vegan* [34]. The abundance of the different phyla and classes were plotted as percentage of total rarefied reads in GraphPad Prism (version 8.2.1). The number of taxa increases with taxonomic rank, which makes relative abundance plots at the order, family and genus taxonomic level difficult to interpret. Thus, those orders, families and genera best separating the different greenspaces were identified and their abundances plotted on heatmaps as Log_2_[Rarefied reads] in Qlucore Omics Explorer (version 3.5). Statistically significant differences in specific taxa across the greenspaces were identified with a PERMANOVA test using the *aovp* function (with 5000 permutations) in *lmPerm* [22, 36], and specific taxa significantly associated with particular greenspaces were determined using the *multipatt* function (with 9999 permutations) in *indicspecies* [37]*.* Finally, ecological guilds were assigned to fungal taxa using FUNGuild (version 1.1) [38], and statistically significant differences between greenspaces were tested using the *aovp* function (with 5000 permutations) in *lmPerm* [22, 36], and specific guilds significantly associated with particular greenspaces were determined using the *multipatt* function (with 9999 permutations) in *indicspecies* [37].


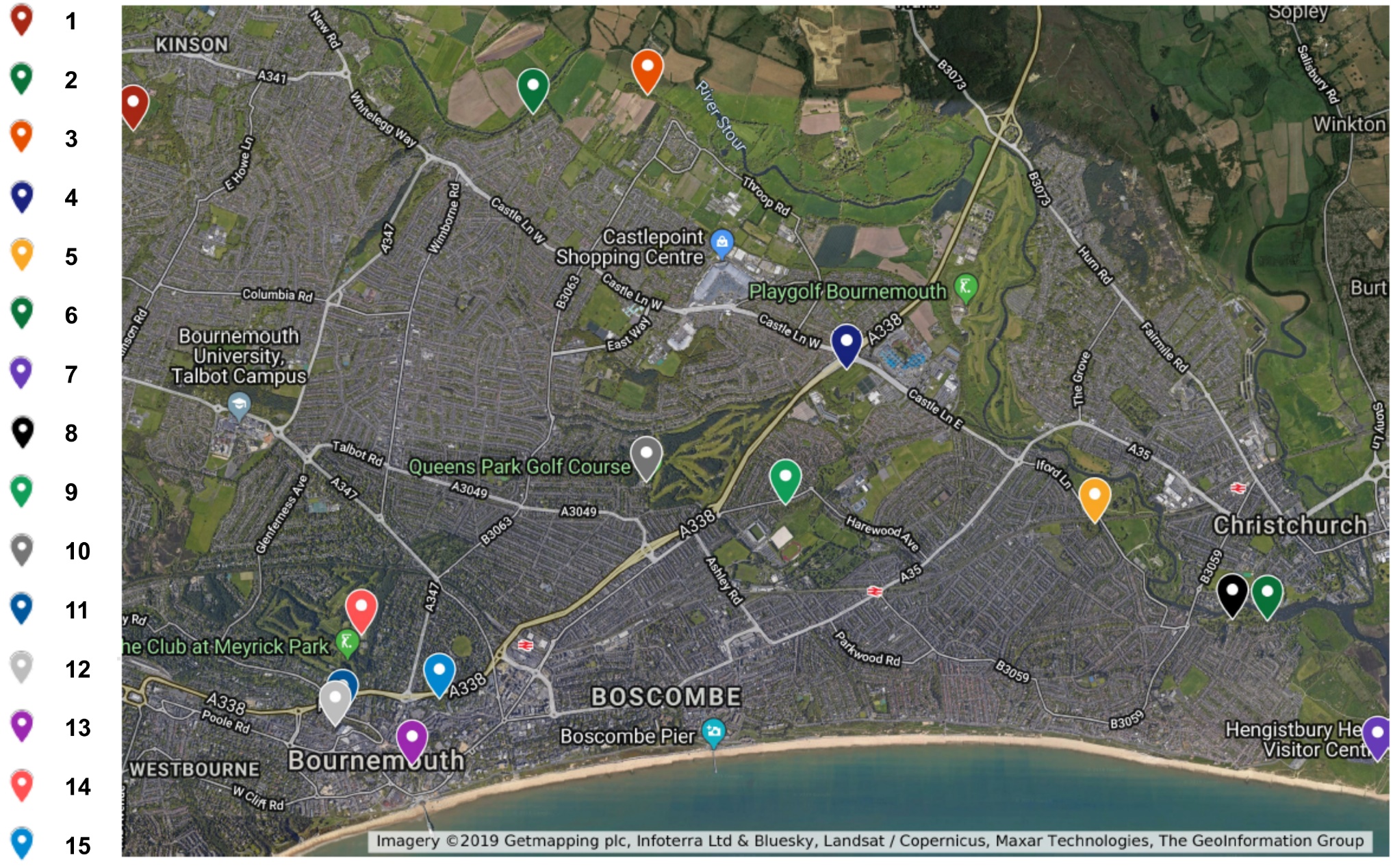
**Supplementary Information 2** Map of 15 sampling sites in and around Bournemouth. Sample site 1: Latitude: 50.76353702 Longitude : -1.907947976; Sample site 2: Latitude: 50.7646819856 Longitude : -1.8632389884; Sample site 3: Latitude: 50.7660499960 Longitude : -1.8504669797; Sample site 4: Latitude: Latitude: 50.7466479857 Longitude : -1.8282560259; Sample site 5: Latitude: 50.7357670180 Longitude : -1.8005310372; Sample site 6: Latitude: 50.7289090287 Longitude : -1.7812610418; Sample site 7: Latitude: 50.7189359888 Longitude : -1.7689490318; Sample site 8: Latitude: 50.7290179934 Longitude : -1.7851700261; Sample site 9: Latitude: 50.7370819710 Longitude : -1.8350509834; Sample site 10: Latitude: 50.7387109939 Longitude : -1.8505949713; Sample site 11: Latitude: 50.71146201 Longitude : -2.428842019; Sample site 12: Latitude: 50.7214239892 Longitude : -1.8853370380; Sample site 13: Latitude: 50.7185250241 Longitude : -1.8767020013; Sample site 14: Latitude: 50.7279049605 Longitude : -1.8824239913; Sample site 15: Latitude: 50.7233310398 Longitude : -1.8737379927.

**Supplementary Information 3** Composition of the mock community. Taken from [1].

| **Species** | **Strain** | **Spore size (μm)** | **Spore complexity** | **Concentration** |
| --- | --- | --- | --- | --- |
| *Alternaria alternata* | EGS 35–193 | 25–60 X 3–3.5 | Multi-cellular (up to 15 cells) | 1 X 10^5^/ml |
| *Aspergillus fumigatus* | NRRL 163 | 2.5–3.5 | Single celled | 1 X 10^5^/ml |
| *Botrytis cinereal* | CABI 160282 | 7–14 X 6–9 | Single celled | 1 X 10^5^/ml |
| *Cladosporium herbarum* | NCPF 2564 | 5.5–23 X 4–6 | One or two celled | 1 X 10^5^/ml |
| *Epicoccum nigrum* | CABI 127257 | 15–25 | Multi-cellular (up to 15 cells) | 1 X 10^5^/ml |
| *Fusarium moniliforme* | NCPF 2865 | 4–7 X 25–50 | 1 to 3 cells | 1 X 10^5^/ml |
| *Leptosphaeria coniothyrium* | CABI 52734 | 3–5 X 15–29 | Multi-cellular (up to 20 cells) | 1 X 10^5^/ml |
| *Penicillium chrysogenum* | NCPF 2715 | 1.8–3.5 | Single celled | 1 X 10^5^/ml |

**Supplementary Information 4** Primer sequences. Fusion primers comprised a region specific priming element ([23-25]; bold), an IonXpress^TM^ barcode (10 nt; N) with the barcode key sequence (italic) that signifies the end of the barcode and the beginning of the region specific sequence, and the Ion Torrent^TM^ adapters (plain text) with the adapter key sequence (underlined) that signifies the end of the adapters and the beginning of the barcode sequence. Barcodes and key sequences were only required on the forward primers.

| **Region** | **Forward Primer** | **Reverse Primer** | **T_m_ (^o^C)** |
| --- | --- | --- | --- |
| ITS1 | CCATCTCATCCCTGCGTGTCTCCGACTCAGNNNNNNNNNN*GAT***TCCGTAGGTGAACCTGCGG** | CCTCTCTATGGGCAGTCGGTGATGCTGCGTTCTTCATCGATGC | 66 |
| ITS2 | CCATCTCATCCCTGCGTGTCTCCGACTCAGNNNNNNNNNN*GAT*GTGAATCATCGAATCTTTGAA | CCTCTCTATGGGCAGTCGGTGATTCCTCCGCTTATTGATATGC | 60 |
| LSU | CCATCTCATCCCTGCGTGTCTCCGACTCAGNNNNNNNNNN*GAT*GAGTCGAGTTGTTTGGGAATGC | CCTCTCTATGGGCAGTCGGTGATGGTCCGTGTTTCAAGACGG | 68 |

**Supplementary Information 5** Numbers of raw and processed reads per region per sample.

| **Sample** | **Raw reads** | **Reads after primer removal** | **Truncated reads** |
| --- | --- | --- | --- |
| Mock community |  |  |  |
| ITS1_MC_1 | 888,888 | 721,358 | 719,765 |
| ITS1_MC_2 | 47,199 | 30,278 | 30,243 |
| ITS1_MC_3 | 245,292 | 133,601 | 133,530 |
|  |  |  |  |
| ITS2_MC_1 | 2,387,049 | 2,105,676 | 2,104,734 |
| ITS2_MC_2 | 131,051 | 113,962 | 113,961 |
| ITS2_MC_3 | 556,640 | 487,875 | 487,870 |
|  |  |  |  |
| LSU_MC_1 | 636,297 | 448,512 | 440,058 |
| LSU_MC_2 | 114,471 | 51,217 | 49,132 |
| LSU_MC_3 | 473,114 | 248,761 | 244,445 |
|  |  |  |  |
| Soil samples |  |  |  |
| ITS1_1 | 271,337 | 197,359 | 197,122 |
| ITS1_2 | 299,840 | 224,945 | 224,288 |
| ITS1_3 | 351,342 | 291,326 | 271,405 |
| ITS1_4 | 302,627 | 225,590 | 225,049 |
| ITS1_5 | 217,661 | 166,669 | 165,595 |
| ITS1_6 | 227,641 | 144,231 | 143,459 |
| ITS1_7 | 300,774 | 221,644 | 217,259 |
| ITS1_8 | 289,442 | 222,518 | 222,103 |
| ITS1_9 | 191,941 | 140,374 | 139,389 |
| ITS1_10 | 193,103 | 123,918 | 122,869 |
| ITS1_11 | 273,284 | 212,319 | 211,087 |
| ITS1_12 | 236,151 | 163,319 | 160,935 |
| ITS1_13 | 183,038 | 142,330 | 141,505 |
| ITS1_14 | 141,056 | 108,767 | 108,528 |
| ITS1_15 | 486,903 | 399,484 | 393,257 |
|  |  |  |  |
| ITS2_1 | 407,508 | 312,628 | 312,342 |
| ITS2_2 | 248,128 | 186,218 | 186,056 |
| ITS2_3 | 370,663 | 222,463 | 222,189 |
| ITS2_4 | 248,267 | 190,860 | 190,845 |
| ITS2_5 | 243,884 | 188,234 | 188,094 |
| ITS2_6 | 246,972 | 187,279 | 187,235 |
| ITS2_7 | 517,418 | 407,998 | 407,913 |
| ITS2_8 | 280,806 | 215,111 | 215,090 |
| ITS2_9 | 817,647 | 616,916 | 616,393 |
| ITS2_10 | 251,588 | 190,732 | 190,710 |
| ITS2_11 | 510,168 | 385,337 | 384,961 |
| ITS2_12 | 236,693 | 169,277 | 169,271 |
| ITS2_13 | 218,928 | 183,887 | 183,880 |
| ITS2_14 | 269,558 | 222,544 | 222,533 |
| ITS2_15 | 653,096 | 533,011 | 532,882 |
|  |  |  |  |
| LSU_1 | 180,569 | 85,661 | 85,426 |
| LSU_2 | 177,187 | 106,136 | 105,226 |
| LSU_3 | 339,010 | 186,999 | 184,861 |
| LSU_4 | 169,874 | 105,803 | 105,507 |
| LSU_5 | 140,376 | 75,757 | 75,358 |
| LSU_6 | 184,877 | 113,421 | 112,426 |
| LSU_7 | 484,556 | 278,239 | 274,764 |
| LSU_8 | 164,836 | 101,882 | 101,492 |
| LSU_9 | 354,569 | 204,653 | 202,945 |
| LSU_10 | 160,599 | 104,880 | 104,325 |
| LSU_11 | 179,231 | 115,086 | 114,073 |
| LSU_12 | 203,490 | 115,032 | 113,817 |
| LSU_13 | 151,538 | 97,349 | 96,056 |
| LSU_14 | 192,324 | 120,453 | 120,151 |
| LSU_15 | 181,908 | 97,673 | 96,735 |
|  |  |  |  |
| Totals |  |  |  |
| Total | 18,232,409 | 13,447,552 | 13,371,144 |
|  |  |  |  |
| Total_ITS1 | 5,147,519 | 3,870,030 | 3,827,388 |
| Total_ITS2 | 8,596,064 | 6,920,008 | 6,916,959 |
| Total_LSU | 4,488,826 | 2,657,514 | 2,626,797 |
|  |  |  |  |
| Per_Soil_Sample_ITS1 | 264,409 | 198,986 | 196,257 |
| Per_Soil_Sample_ITS2 | 368,088 | 280,833 | 280,693 |
| Per_Soil_Sample_LSU | 217,663 | 127,268 | 126,211 |

Note: The first sequencing run was a test run for the mock community, thus there were a greater number of reads for all three regions of sample MC_1.

**Supplementary Information 6** Octave plots of alpha diversity **(A)** ITS1, **(B)** ITS2 and **(C)** LSU. These plots visualise the OTU abundance distribution and thus diversity of each sample, allowing assessment of whether sampling is complete, truncated or J-shaped [33]. Complete is well-modelled by a complete log-normal distribution and signifies 100% of the diversity has been sampled. Truncated is well-modelled by a truncated log-normal distribution and signifies over 50% of the diversity has been sampled. J-shaped (common with microbial OTU data) can be well-modelled by a log-series or truncated-normal distribution, but is difficult to interpret with respect to sampling efficiency. The mock community samples were composed of much fewer OTUs and thus difficult to see as part of the pooled analysis (with mock community 2 being lost through the normalisation process). Therefore, the individual octave plots are also shown.


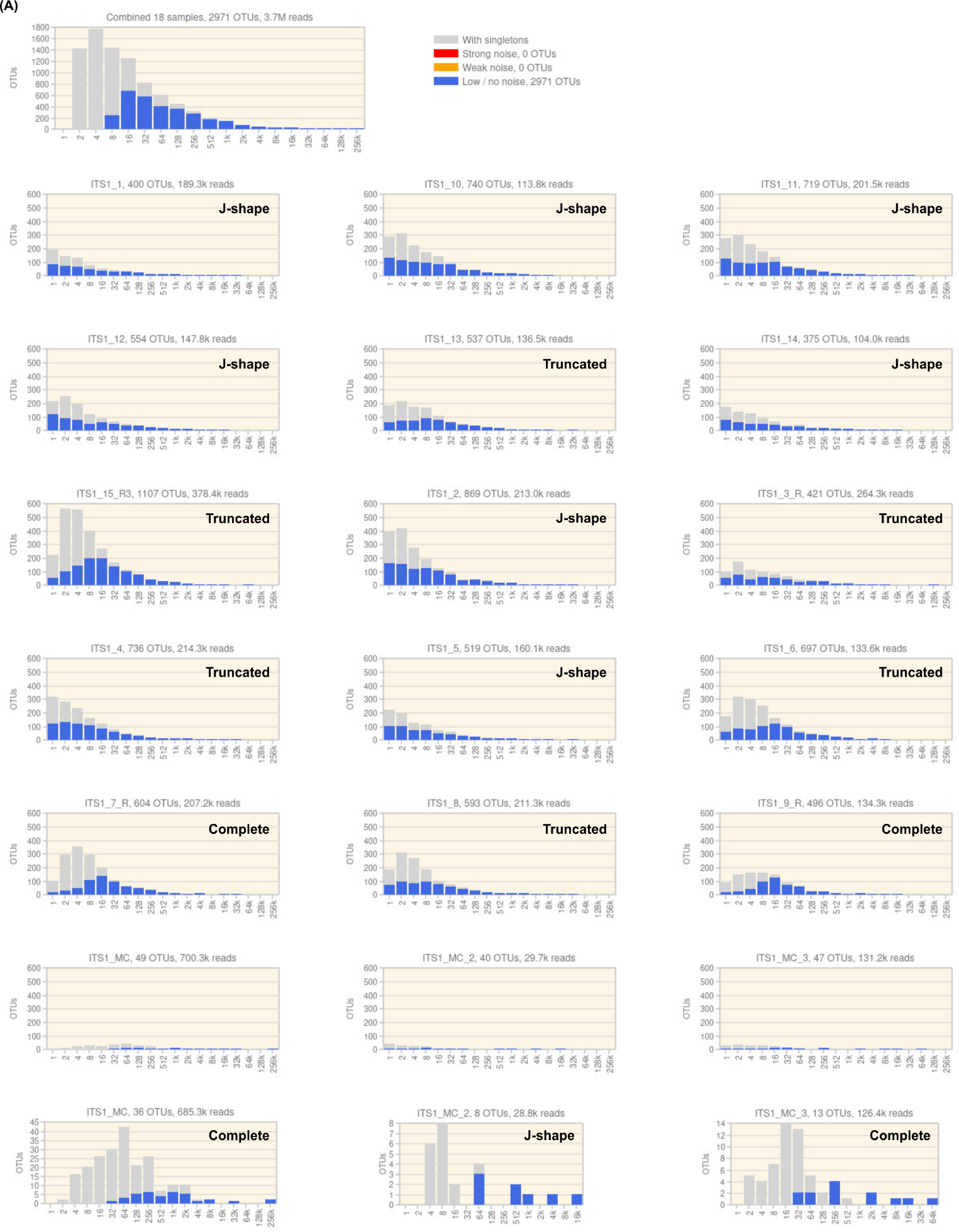


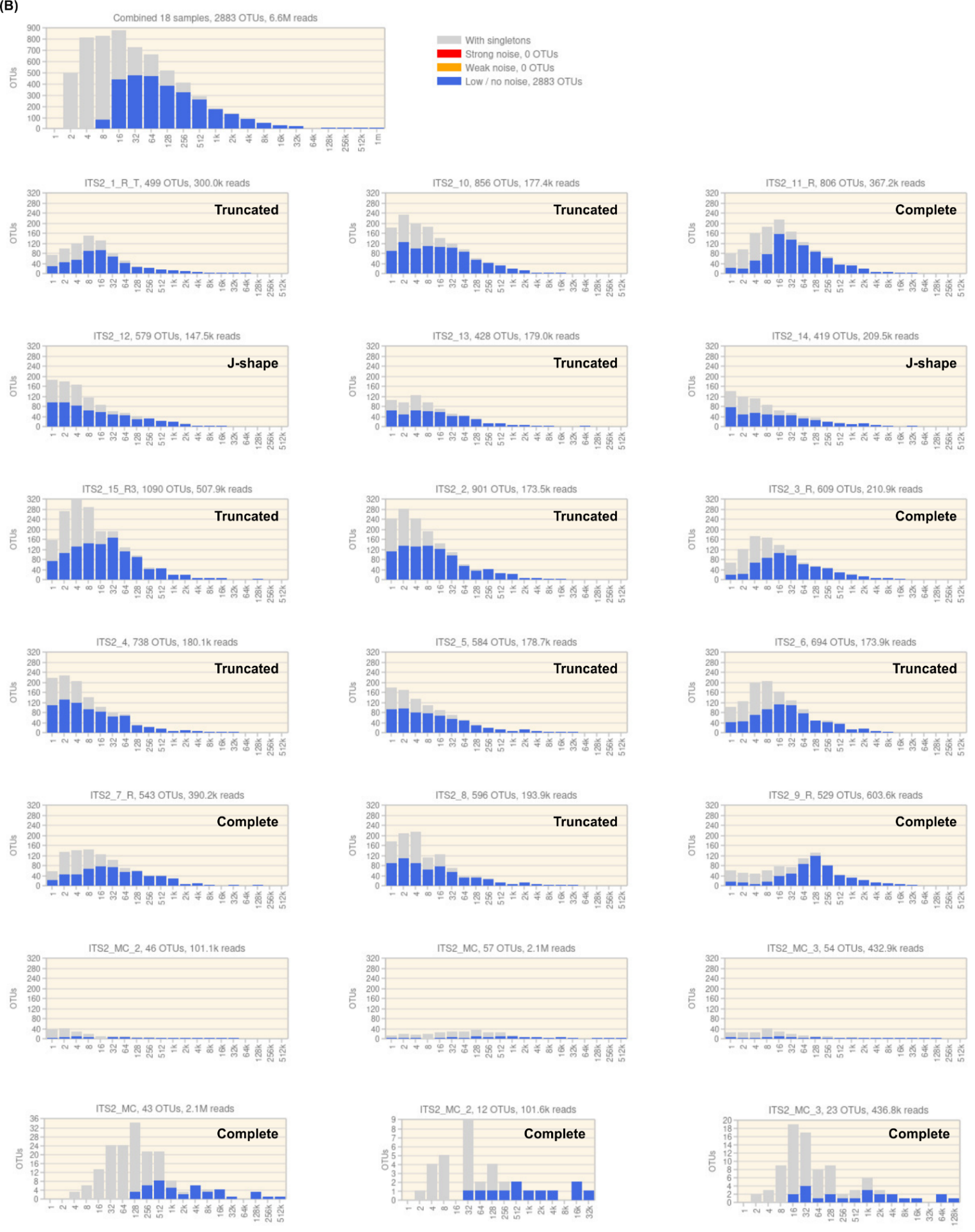


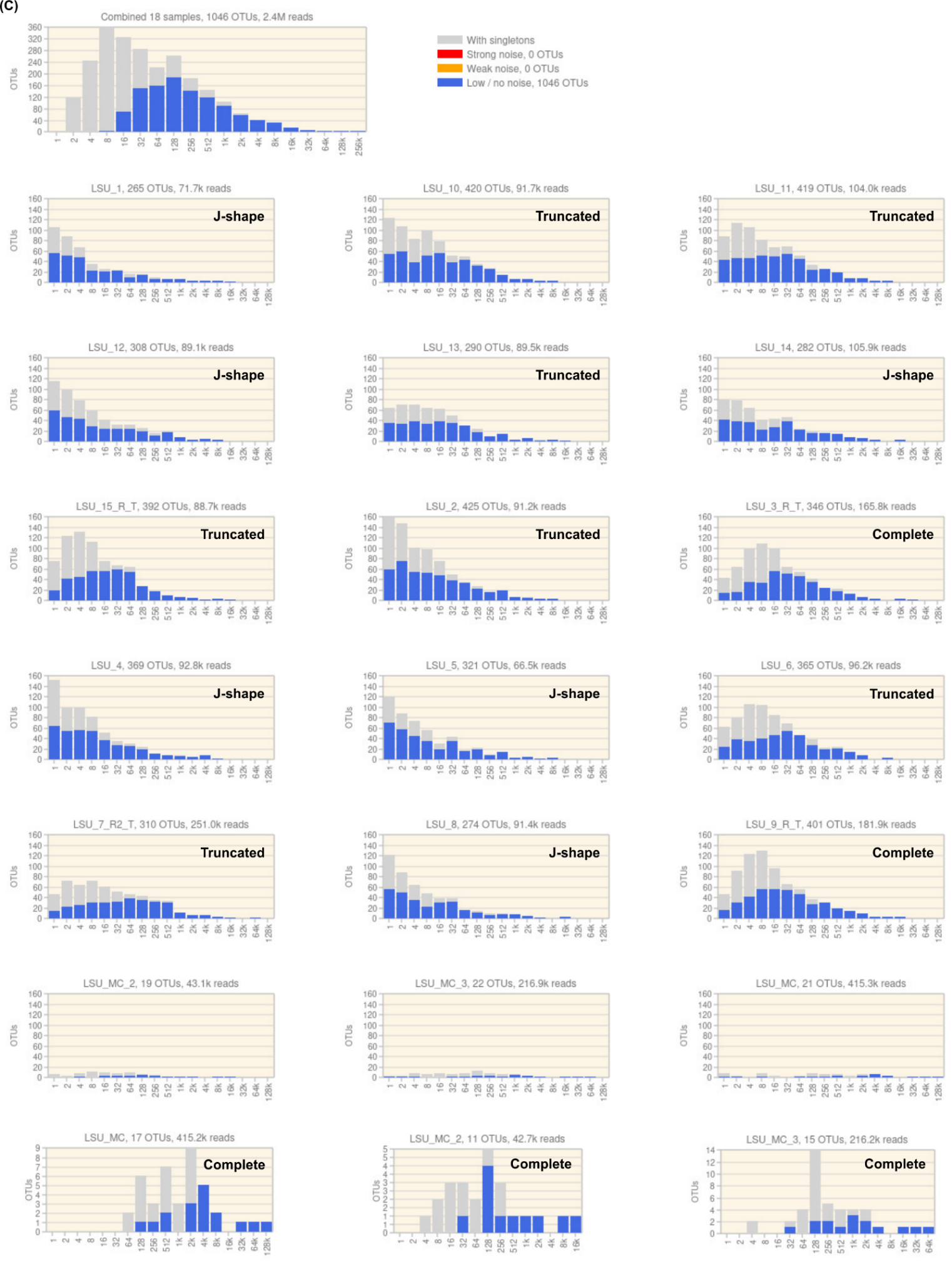


ITS2 sampled the greatest OTU diversity (where more than half or complete diversity was captured in 89% of the samples), followed by LSU (where more than half or complete diversity was captured in 67% of samples), and finally LSU (where more than half or complete diversity was captured in 60% of the samples). Consequently, Shannon entropy is the recommended diversity metric for sample comparison [33]. These plots validated the discarding of OTUs with a size <10 sequences and demonstrated that Shannon entropy is the recommended diversity metric for sample comparison, although taxon diversity metrics should be interpreted with caution as full diversity was not captured for all samples [33].

**Supplementary Information 7** Detailed mock community analysis across the three sequencing runs. Samples were analysed by region **(A)** ITS1, **(B)** ITS2 and **(C)** LSU as separate runs (i Alone), following pooling of all three runs (ii Pooled) plus pooling with the soil samples across all three runs, OTU frequency trimming and normalisation (iii Pooled, Trimmed & Normalised). Combined analysis of all three regions following pooling of mock community and soil samples plus OTU trimming and normalisation is shown in **(D)**.

**
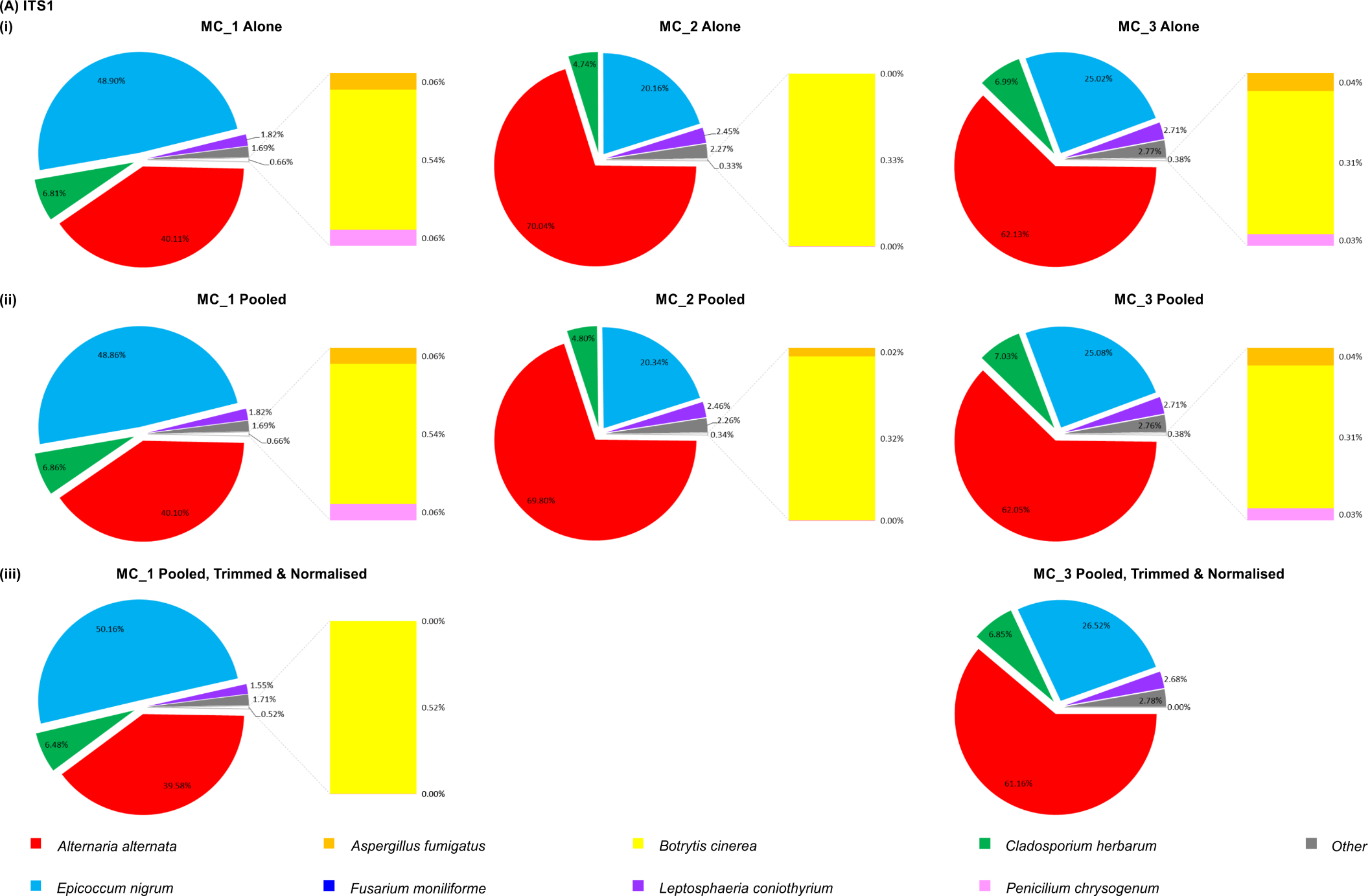
**


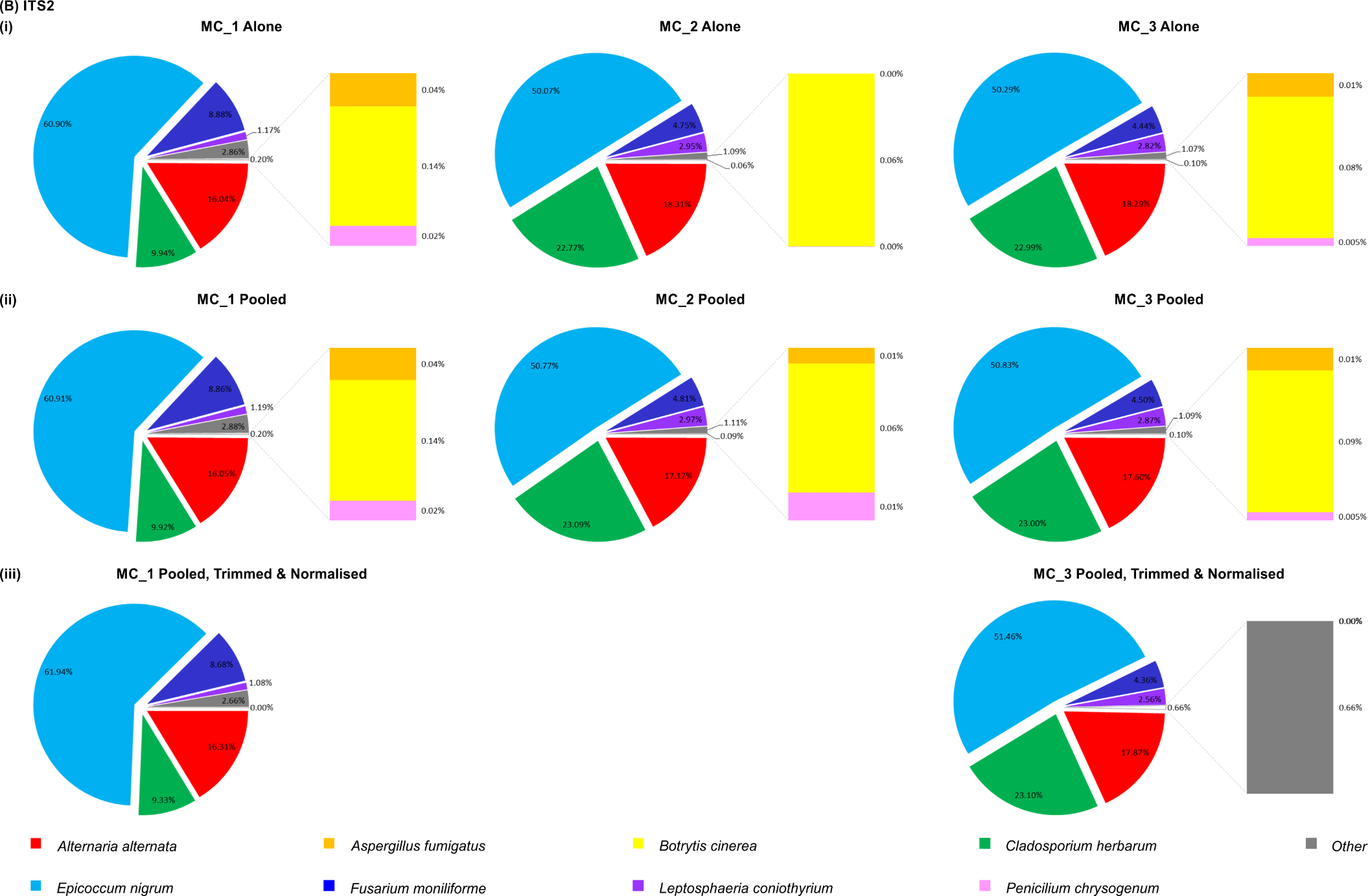


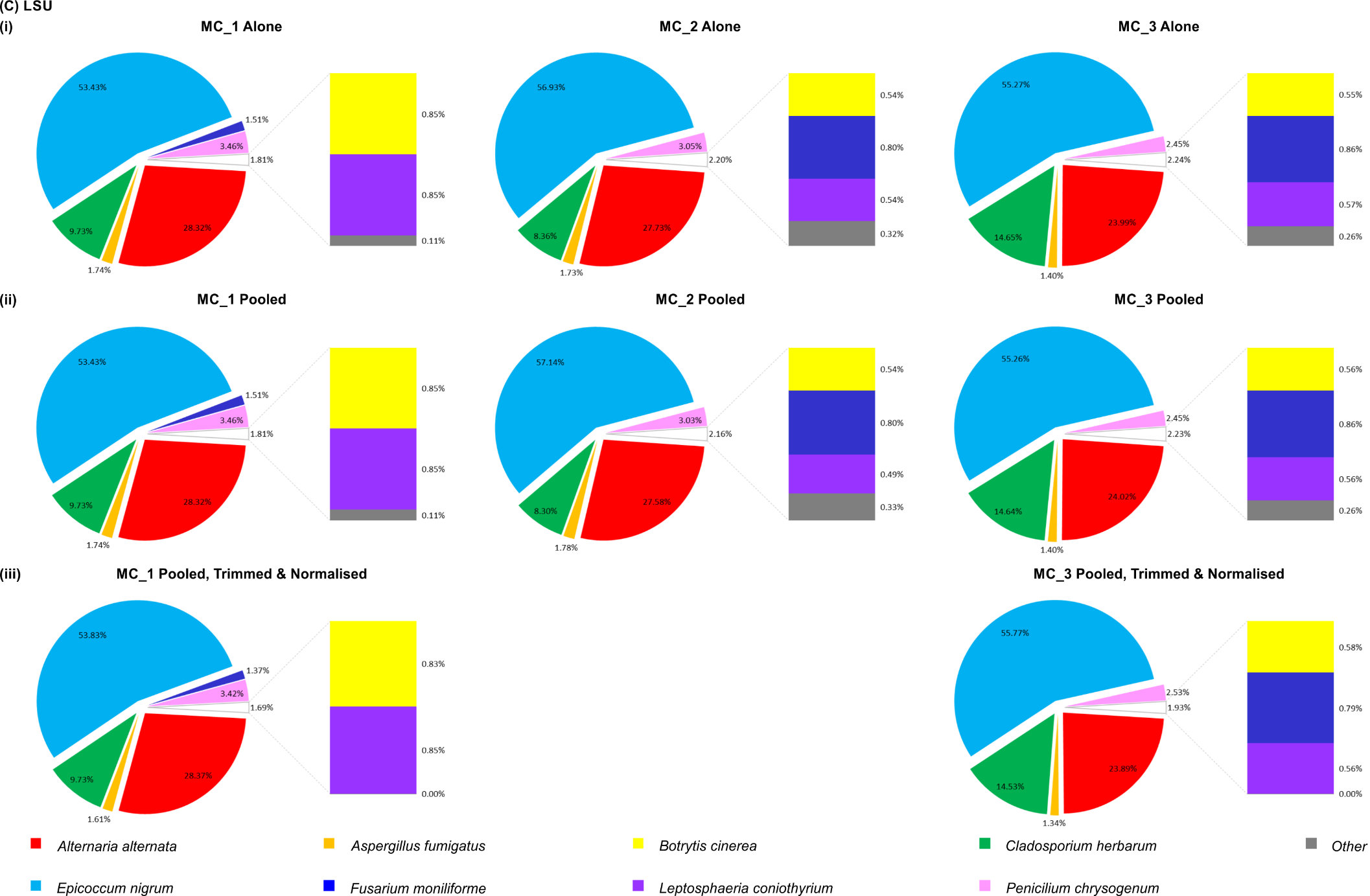


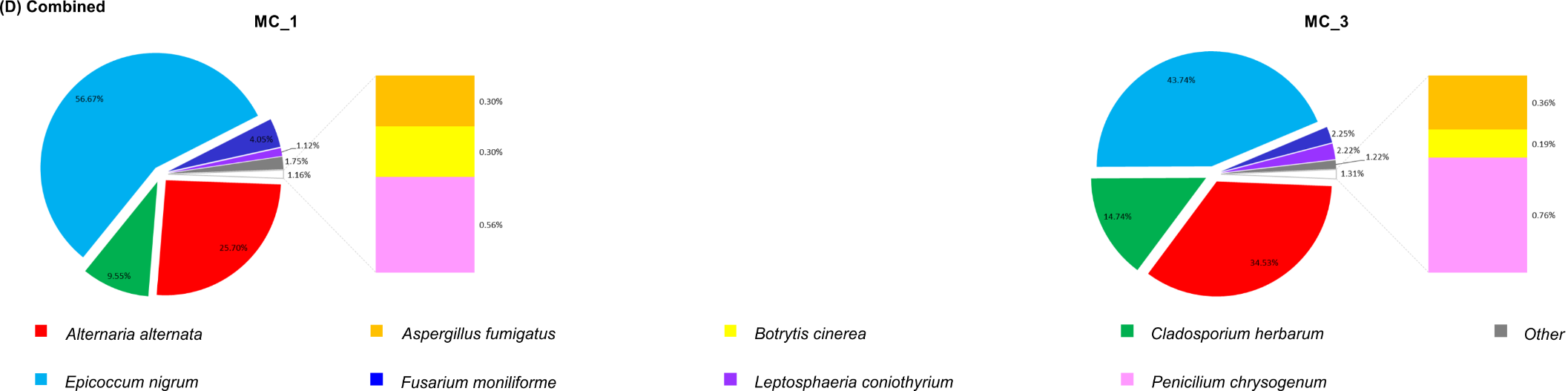


When analysed individually, ITS2 and LSU successfully identified all 8 species within the mock community, with ITS1 identifying seven of the eight species (A-C). This is in keeping with our previous work [1]. There is a mismatch between in the ITS1 region in *Fusarium moniliforme* and the forward ITS1 primer, reducing efficient amplification and resulting in the loss of any sequencing data during removal of primer sequences (where a 100% primer match was required) [1].

The results were similar across the three regions, with *Epicoccum nigrum*, *Alternaria alternata* and *Cladosporium herbarum* accounting for >90% of the total reads. However, there were some differences in the relative abundances of the mock community species, depending upon the region analysed. For example, ITS1 assigned a greater proportion of reads to *Alternaria alternata* than ITS2 or LSU; ITS2 assigned a greater proportion of reads to *Fusarium moniliforme* than the other two regions; and LSU assigned a greater proportion of reads to *Penicillium chrysogenum* and *Aspergillus fumigatus* with a reduced number of reads assigned to other (species not present in the mock community) than ITS1 or ITS2. In addition, there were differences in the relative abundances of the mock community species in MC_1 *vs* MC_2 and MC_3 vs [1], with greater differences between [1] and MC_1/MC_2/MC_3. The differences between MC_1 compared to MC_2 and MC_3 was likely a result of MC_1 being a different batch of mock community to MC_2 and MC_3. Both batches were produced from the same stock of fungal DNA, suggesting that small differences in PCR amplification between batches can result in small changes in relative abundance. The larger differences in species abundance in our previous study [1] compared with this study was likely a result of modified primers (with barcodes) being used in the latter. Modified primers may introduce amplification bias, resulting in altered relative abundances [1].

It is also worth noting that the mock community run with the lowest number of reads was MC_2. Neither *Aspergillus fumigatus* nor *Penicillium chrysogenum* were detected by the ITS1 or ITS2 regions in MC_2, likely due to reduced number of reads and thus reduced diversity sampling (Ai & Bi). The LSU region, however, detected all eight mock community species in MC_2, despite the lower number of reads (Ci).

Pooling the three mock community samples prior to dereplication had no effect on the results of the LSU region, but increased detection of the mock community species in MC_2 to 6/8 and 8/8 for the ITS1 and ITS2 regions, respectively (Aii-Cii). Metagenomic sequencing is prone to generating large numbers of spurious OTUs due to amplification and sequencing errors [27, 39], which can misrepresent diversity [40]. Thus, when generating OTUs, it is recommended that sequences present exactly once (singletons) are discarded [41]. Here we discarded sequences with a size of <10 (sequences present nine times or less) as an alternative to the quality filtering commands that are not suitable for ion torrent data [31]. Some of these low abundant sequences will represent real biological OTUs. Sequences with sizes <10 in one sample may also be present in other samples. Pooling samples prior to dereplication, (the step within which sequences are discarded), increases the inclusion of such low abundant sequences in the final OTU table compared with discarding sequences for each sample separately [30].

When the mock community samples were pooled and analysed with the soil samples, which involved trimming of OTUs with a frequency of <0.5% and normalisation (rarefaction), there was the expected loss of both low abundant OTUs (*Aspergillus fumigatus*, *Penicillium chrysogenum* and *Botryitis cinera*) from ITS1 and/or ITS2 results and MC_2 from all region results (Aiii and Biii). LSU was the only region that successfully detected all 8 mock community species in MC_1 and MC_3 following OTU frequency trimming and normalisation (Ciii).

Combining the results of all three regions maximised fungal identification in the mock community samples MC_1 and MC_3 (D). Thus, analysing multiple regions provides additional supporting data and maximises successful analysis. It was therefore decided that final analysis would include removal of OTUs with a frequency <0.5% and normalisation to a number that excluded MC_2 since: 1) the number of reads for MC_2 was so much lower than the other samples, 2) LSU alone and in combination with ITS1 and ITS2 successfully detected all 8 mock community species following trimming and normalisation, and 3) there was a balance between identifying real biological OTUs *versus* removing spurious OTUs to reduce noise.

It is also worth noting that while the mock community was prepared from an equal number of spores from each species, the results suggested a wide range of relative abundances from 0.01% to >60% (A-D). These variations in relative abundance will be due to a combination of differences in: 1) spore size and complexity, which determine the amount of DNA per spore, 2) DNA extraction efficiencies between fungal spore types and/or between spores and other fungal tissues such as mycelia, 3) PCR amplification biases dependent upon both the primers used and the amplicon length, 4) GC content and homopolymers, which affect DNA polymerase efficiency, and 5) the number of copies (copy number variation, (CNV)) of the three regions between fungal species [1, 42, 43]. Relative OTU abundances derived from metabarcoding analysis are therefore unlikely to represent the true abundances of fungal species. Nevertheless, while it is not appropriate to compare the relative abundance of different fungal species within the same sample, it is possible to make valid comparisons of the same fungal species analysed in the same way across different samples [44].

Analysis of the mock community samples therefore: 1) validated the bioinformatic pipeline, 2) highlighted the advantage of pooling sample reads prior to dereplication to enhance the signal of low abundant sequences and increase the detection of real biological OTUs over background noise, 2), demonstrated the use of a mock community as a quality control for each sequencing run (in terms of both sequencing and bioinformatic analysis), 4) established the added value of a multi-region approach, and 5) emphasised that relative OTU abundance does not reliably reflect true species abundance and so should only be compared between and not within samples.

**Supplementary Information 8** Fungal taxa identified by (i) ITS1, (ii) ITS2 and (iii) LSU within the soils from five urban greenspaces (lawns, bareground, parklands, young forest and old forest). Only those taxa best separating the urban greenspace soils are shown at the order, family and genus taxonomic levels. Combined analysis of all three regions is shown for comparison in (iiii).


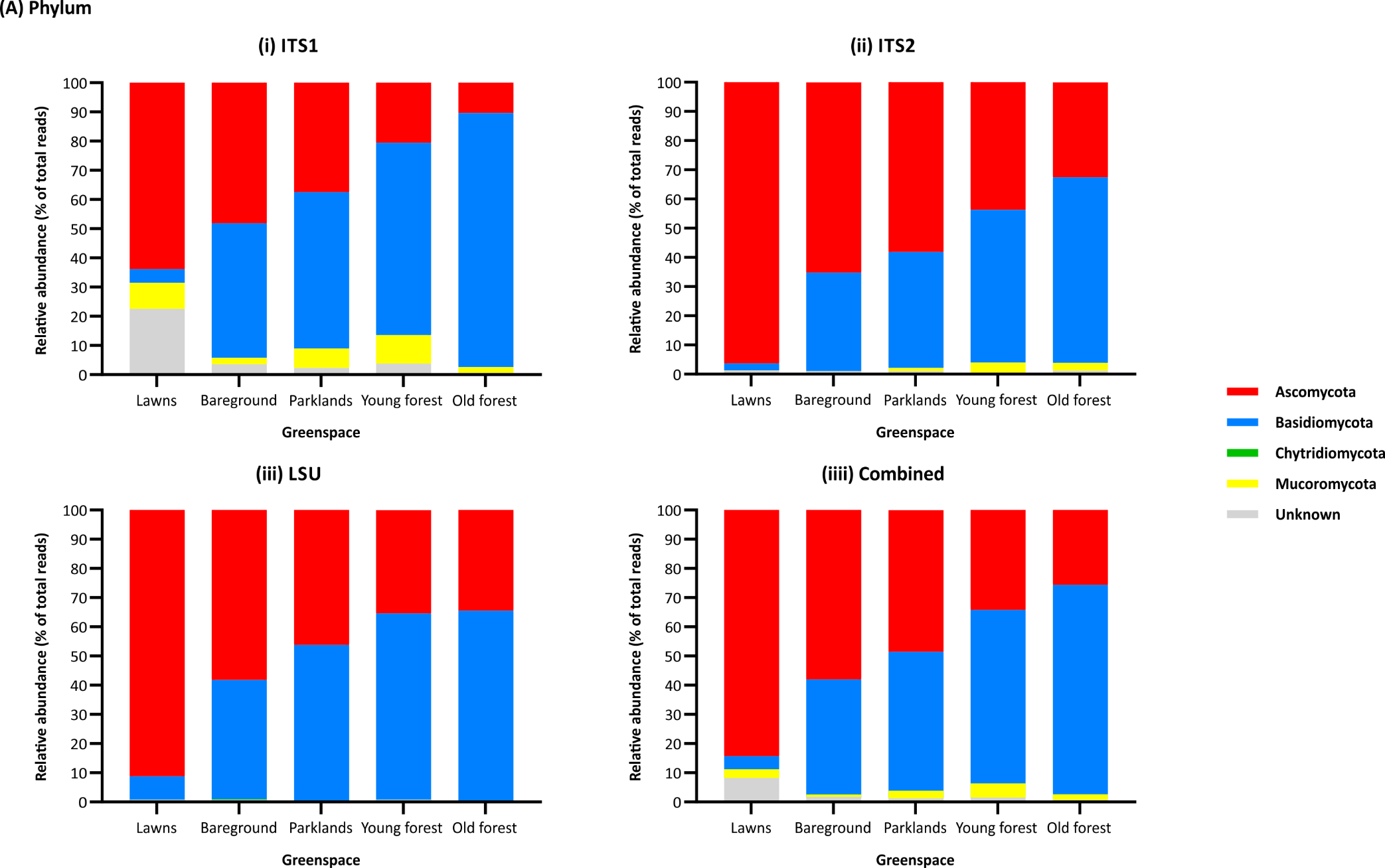


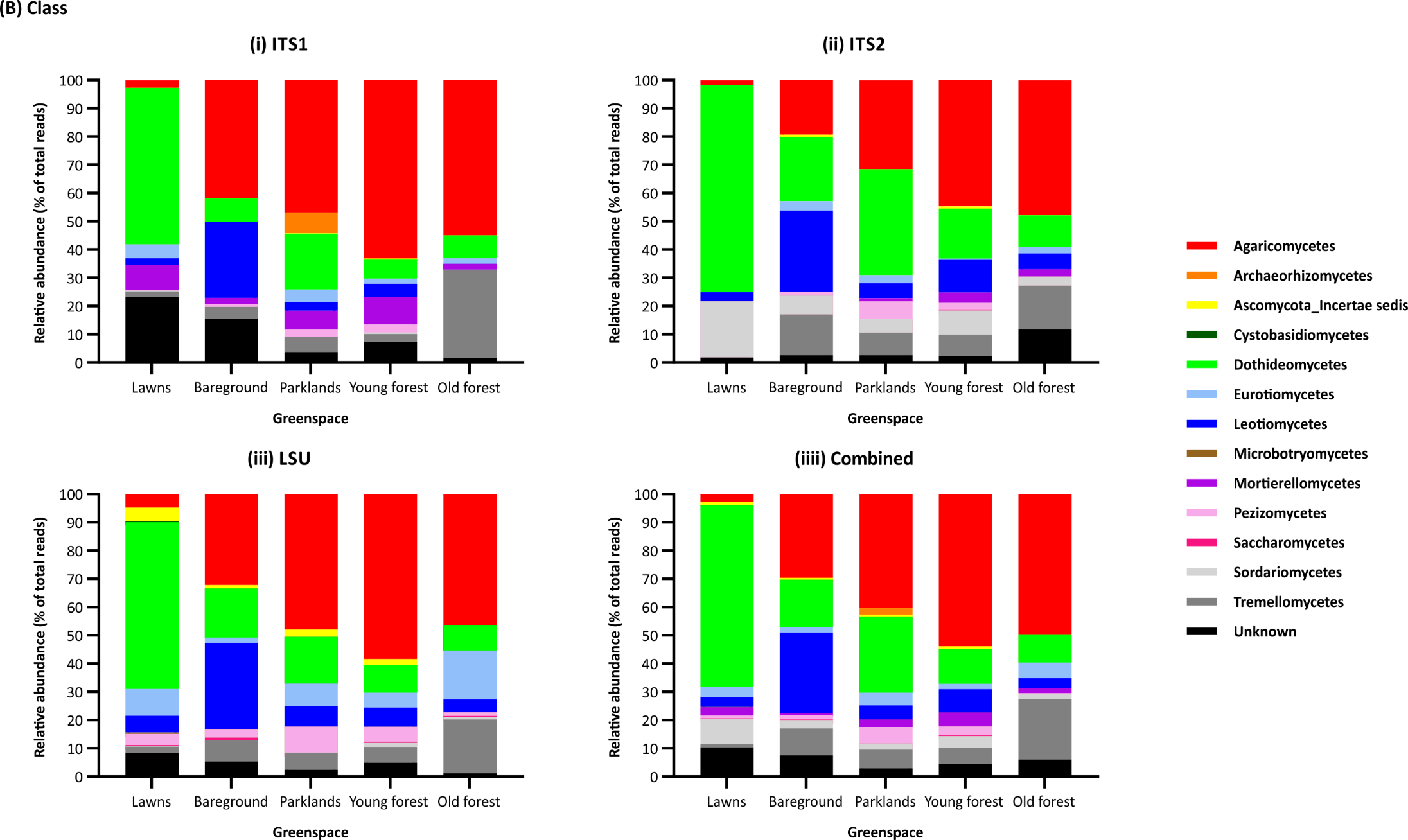


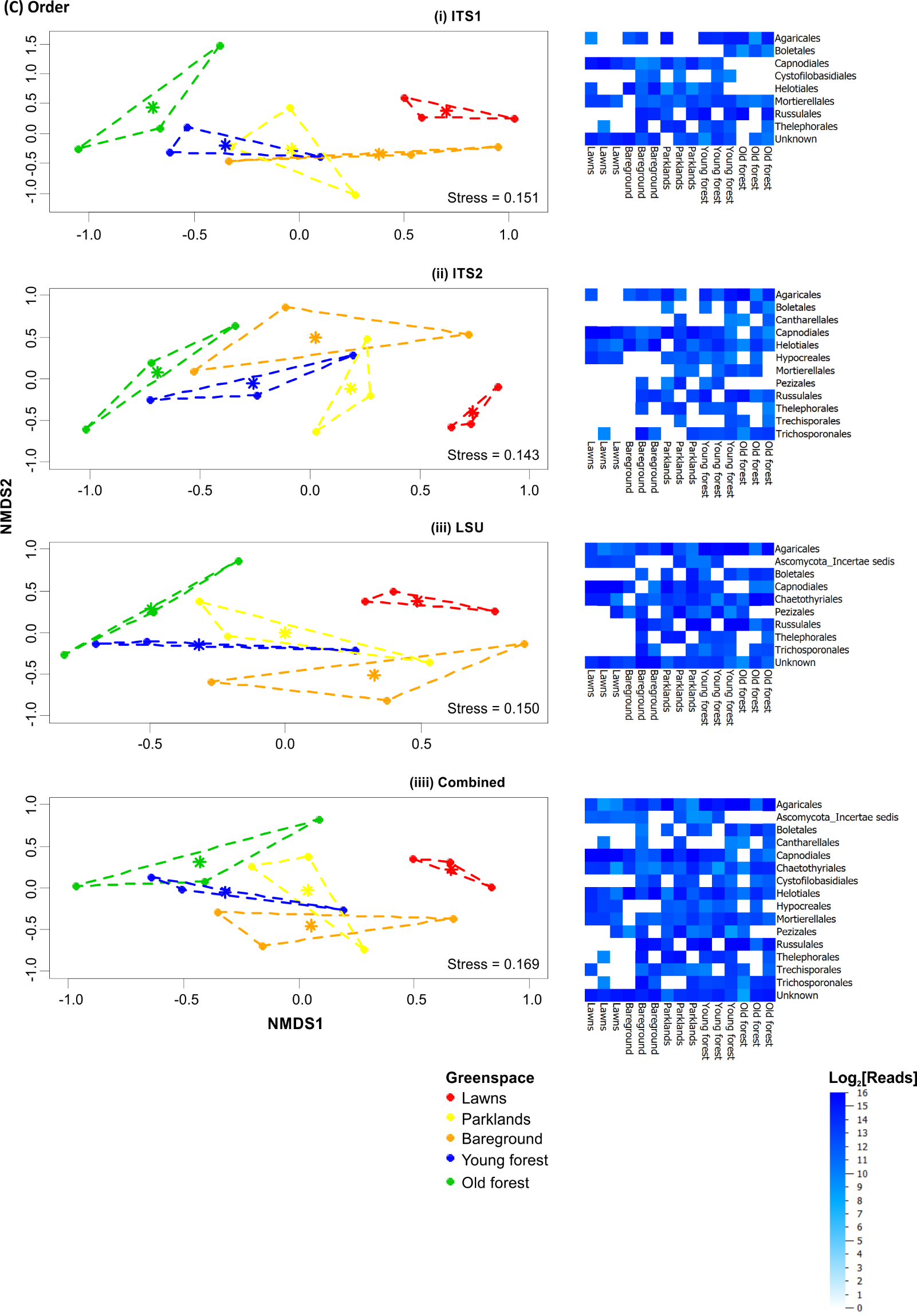


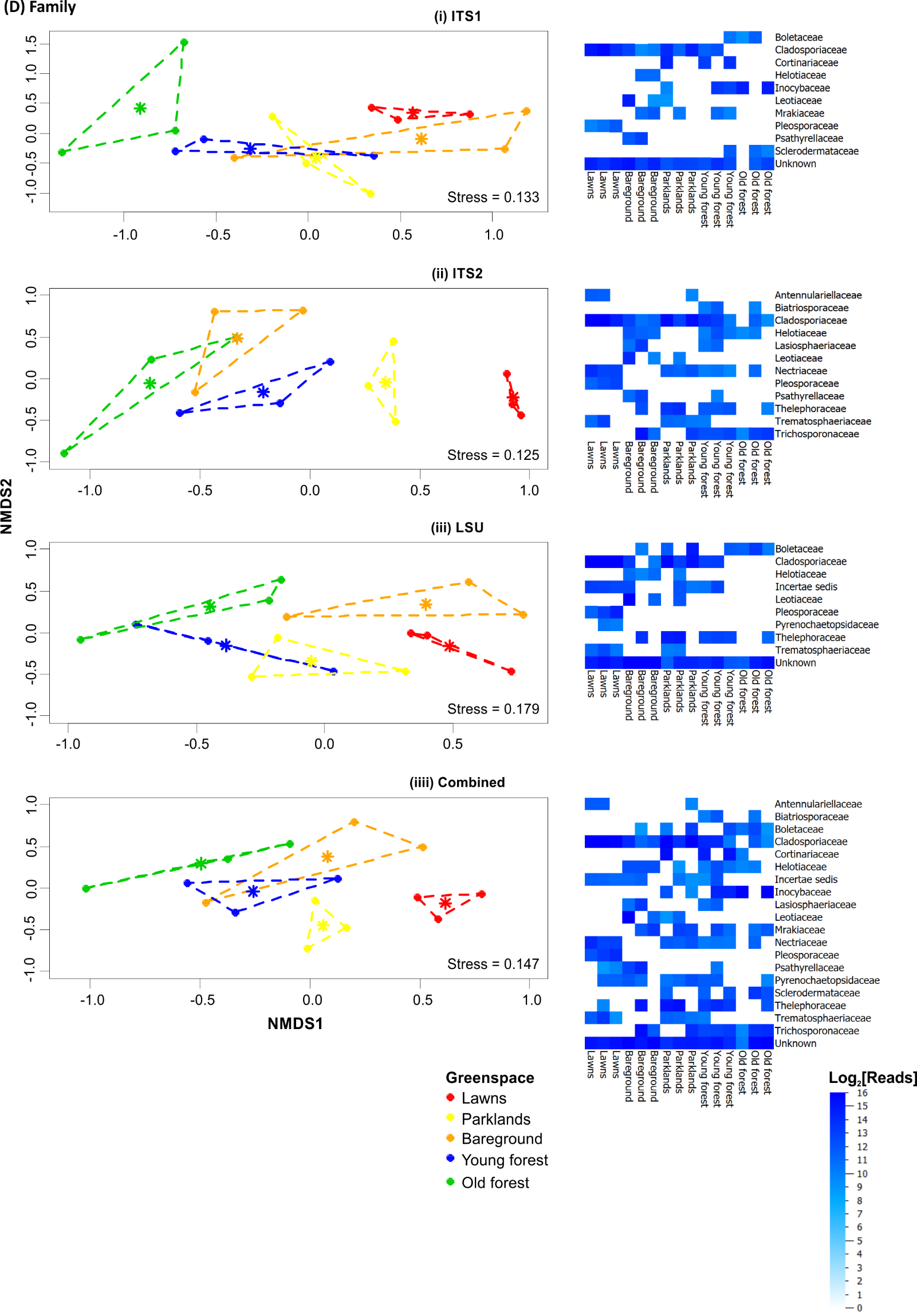


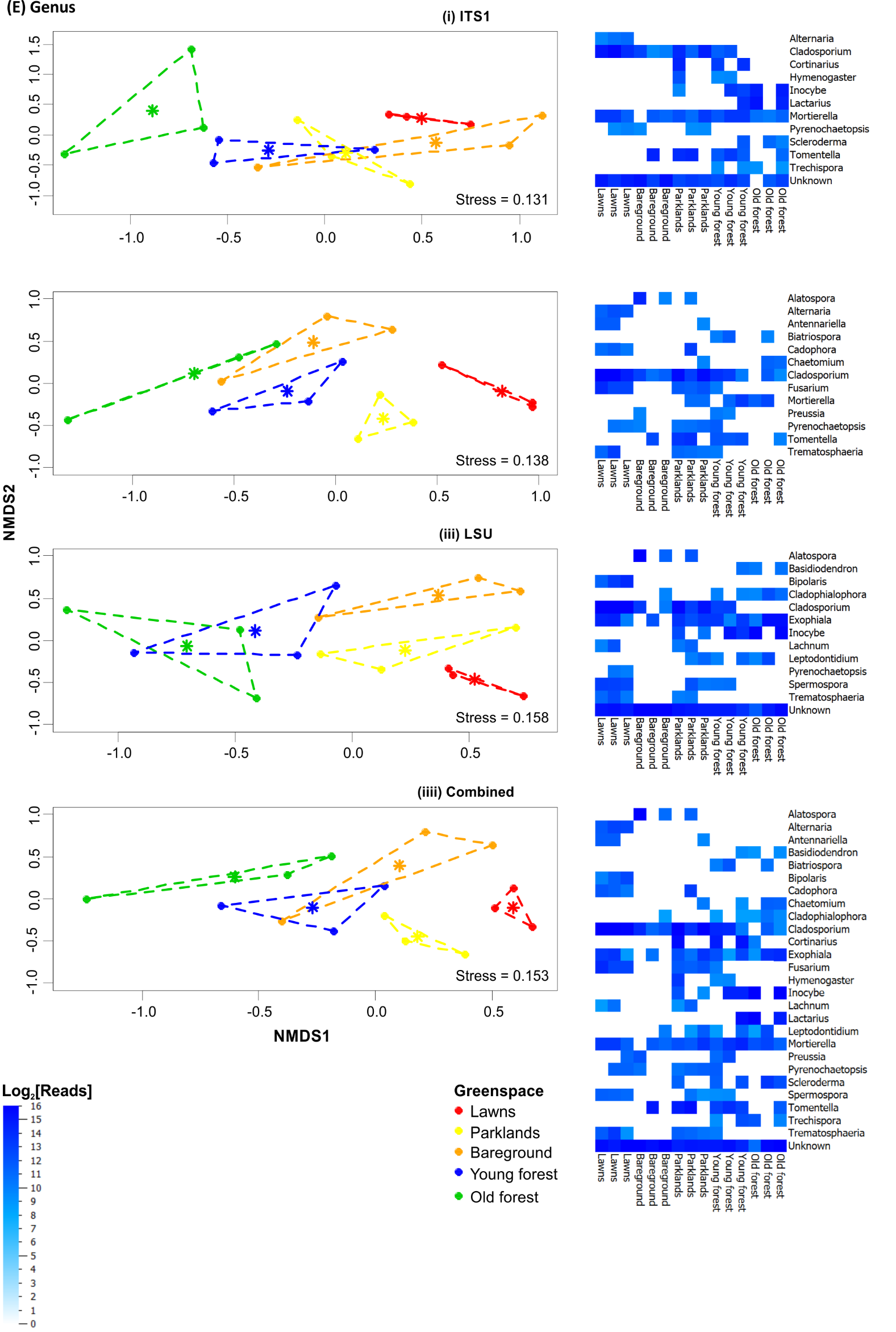


**Supplementary Information 9** Normalised reads for fungal phyla, classes, orders, families and genera within each sample for the individual and combined regions.

| **Phylum** | **ITS1_1** | **ITS1_2** | **ITS1_3** | **ITS1_4** | **ITS1_5** | **ITS1_6** | **ITS1_7** | **ITS1_8** | **ITS1_9** | **ITS1_10** | **ITS1_11** | **ITS1_12** | **ITS1_13** | **ITS1_14** | **ITS1_15** |
| --- | --- | --- | --- | --- | --- | --- | --- | --- | --- | --- | --- | --- | --- | --- | --- |
| Ascomycota | 4932 | 9688 | 572 | 21694 | 21160 | 35299 | 46285 | 78554 | 8087 | 12064 | 37870 | 33490 | 61865 | 56676 | 37019 |
| Basidiomycota | 79386 | 71815 | 82821 | 59262 | 62329 | 35576 | 6736 | 3823 | 62551 | 68656 | 41673 | 46505 | 3688 | 1624 | 37453 |
| Mucoromycota | 1682 | 3894 | 1230 | 5044 | 2511 | 10692 | 10095 | 0 | 10692 | 3563 | 1991 | 2301 | 10091 | 2872 | 10171 |
| Unknown | 0 | 603 | 1377 | 0 | 0 | 4433 | 22884 | 3623 | 4670 | 1717 | 4466 | 3704 | 10356 | 24828 | 1357 |
| **Total** | **86000** | **86000** | **86000** | **86000** | **86000** | **86000** | **86000** | **86000** | **86000** | **86000** | **86000** | **86000** | **86000** | **86000** | **86000** |

| **Class** | **ITS1_1** | **ITS1_2** | **ITS1_3** | **ITS1_4** | **ITS1_5** | **ITS1_6** | **ITS1_7** | **ITS1_8** | **ITS1_9** | **ITS1_10** | **ITS1_11** | **ITS1_12** | **ITS1_13** | **ITS1_14** | **ITS1_15** |
| --- | --- | --- | --- | --- | --- | --- | --- | --- | --- | --- | --- | --- | --- | --- | --- |
| Agaricomycetes | 79386 | 70138 | 6135 | 58657 | 56287 | 33433 | 4009 | 3823 | 58713 | 61880 | 40460 | 42341 | 2545 | 606 | 21838 |
| Archaeorhizomycetes | 0 | 0 | 0 | 0 | 0 | 0 | 0 | 0 | 894 | 0 | 18832 | 0 | 0 | 0 | 0 |
| Ascomycota_Incertae sedis | 0 | 0 | 0 | 0 | 0 | 865 | 0 | 0 | 0 | 0 | 556 | 0 | 0 | 0 | 0 |
| Dothideomycetes | 4932 | 4513 | 572 | 18691 | 15426 | 8943 | 33866 | 13944 | 3946 | 6690 | 5921 | 1058 | 54183 | 54719 | 26336 |
| Eurotiomycetes | 0 | 1429 | 0 | 1321 | 5102 | 0 | 5851 | 0 | 3247 | 0 | 0 | 0 | 6896 | 0 | 10029 |
| Leotiomycetes | 0 | 3746 | 0 | 511 | 0 | 8094 | 5899 | 33706 | 0 | 3050 | 6713 | 32432 | 0 | 0 | 654 |
| Mortierellomycetes | 1682 | 3894 | 1230 | 5044 | 2511 | 10692 | 10095 | 0 | 10692 | 3563 | 1991 | 2301 | 10091 | 2872 | 10171 |
| Pezizomycetes | 0 | 0 | 0 | 1171 | 0 | 7406 | 0 | 0 | 0 | 2324 | 5848 | 0 | 0 | 777 | 0 |
| Sordariomycetes | 0 | 0 | 0 | 0 | 0 | 1198 | 0 | 0 | 0 | 0 | 0 | 0 | 0 | 508 | 0 |
| Tremellomycetes | 0 | 1677 | 75505 | 605 | 5250 | 2143 | 2727 | 0 | 3838 | 6776 | 1213 | 4164 | 1143 | 1018 | 11941 |
| Unknown | 0 | 603 | 2558 | 0 | 1424 | 13226 | 23553 | 34527 | 4670 | 1717 | 4466 | 3704 | 11142 | 25500 | 5031 |
| **Total** | **86000** | **86000** | **86000** | **86000** | **86000** | **86000** | **86000** | **86000** | **86000** | **86000** | **86000** | **86000** | **86000** | **86000** | **86000** |

| **Order** | **ITS1_1** | **ITS1_2** | **ITS1_3** | **ITS1_4** | **ITS1_5** | **ITS1_6** | **ITS1_7** | **ITS1_8** | **ITS1_9** | **ITS1_10** | **ITS1_11** | **ITS1_12** | **ITS1_13** | **ITS1_14** | **ITS1_15** |
| --- | --- | --- | --- | --- | --- | --- | --- | --- | --- | --- | --- | --- | --- | --- | --- |
| Agaricales | 27118 | 17218 | 502 | 32592 | 26145 | 15392 | 752 | 3823 | 25692 | 9312 | 0 | 0 | 0 | 0 | 0 |
| Archaeorhizomycetales | 0 | 0 | 0 | 0 | 0 | 0 | 0 | 0 | 0 | 0 | 18832 | 0 | 0 | 0 | 0 |
| Ascomycota_Incertae sedis | 0 | 0 | 0 | 0 | 0 | 865 | 0 | 0 | 0 | 0 | 556 | 0 | 0 | 0 | 0 |
| Atheliales | 0 | 0 | 0 | 0 | 0 | 0 | 0 | 0 | 0 | 0 | 14582 | 0 | 0 | 0 | 0 |
| Auriculariales | 0 | 0 | 572 | 4697 | 0 | 0 | 0 | 0 | 0 | 0 | 1438 | 1679 | 840 | 0 | 0 |
| Boletales | 557 | 0 | 5061 | 0 | 956 | 0 | 0 | 0 | 6326 | 0 | 0 | 0 | 0 | 0 | 0 |
| Cantharellales | 0 | 0 | 0 | 0 | 0 | 0 | 0 | 0 | 0 | 1084 | 1981 | 0 | 964 | 0 | 0 |
| Capnodiales | 0 | 3041 | 0 | 16592 | 0 | 4410 | 33018 | 7214 | 0 | 663 | 2613 | 1058 | 51863 | 15546 | 24201 |
| Chaetothyriales | 0 | 1429 | 0 | 1321 | 5102 | 0 | 4981 | 0 | 3247 | 0 | 0 | 0 | 6896 | 0 | 9191 |
| Cystofilobasidiales | 0 | 0 | 0 | 0 | 0 | 2143 | 0 | 0 | 842 | 1870 | 1213 | 3539 | 0 | 0 | 0 |
| Dothideales | 0 | 0 | 0 | 0 | 0 | 0 | 0 | 1211 | 0 | 0 | 0 | 0 | 0 | 0 | 0 |
| Eurotiales | 0 | 0 | 0 | 0 | 0 | 0 | 0 | 0 | 0 | 0 | 0 | 0 | 0 | 0 | 838 |
| Filobasidiales | 0 | 0 | 0 | 0 | 0 | 0 | 0 | 0 | 0 | 0 | 0 | 0 | 0 | 1018 | 0 |
| Helotiales | 0 | 3746 | 0 | 511 | 0 | 5973 | 5899 | 33706 | 0 | 3050 | 6713 | 32432 | 0 | 0 | 654 |
| Mortierellales | 1682 | 3894 | 1230 | 5044 | 2511 | 10692 | 10095 | 0 | 10692 | 3563 | 1991 | 2301 | 10091 | 2872 | 10171 |
| Mytilinidiales | 4932 | 916 | 0 | 2099 | 8497 | 0 | 0 | 0 | 3946 | 4912 | 2662 | 0 | 0 | 0 | 0 |
| Pezizales | 0 | 0 | 0 | 1171 | 0 | 7406 | 0 | 0 | 0 | 2324 | 5848 | 0 | 0 | 777 | 0 |
| Pleosporales | 0 | 556 | 572 | 0 | 6929 | 4533 | 848 | 5519 | 0 | 1115 | 646 | 0 | 2320 | 39173 | 2135 |
| Polyporales | 0 | 0 | 0 | 0 | 0 | 6786 | 0 | 0 | 0 | 0 | 0 | 0 | 0 | 0 | 0 |
| Russulales | 51176 | 48462 | 0 | 4094 | 26582 | 0 | 0 | 0 | 22836 | 24144 | 0 | 18829 | 0 | 0 | 19955 |
| Sebacinales | 0 | 0 | 0 | 0 | 0 | 915 | 0 | 0 | 0 | 1369 | 2279 | 0 | 0 | 606 | 0 |
| Thelebolales | 0 | 0 | 0 | 0 | 0 | 2121 | 0 | 0 | 0 | 0 | 0 | 0 | 0 | 0 | 0 |
| Thelephorales | 0 | 3828 | 0 | 15658 | 2113 | 10340 | 0 | 0 | 3200 | 21433 | 20180 | 0 | 741 | 0 | 0 |
| Trechisporales | 535 | 630 | 0 | 1616 | 491 | 0 | 3257 | 0 | 659 | 1539 | 0 | 6366 | 0 | 0 | 0 |
| Tremellales | 0 | 1677 | 73277 | 605 | 3291 | 0 | 2727 | 0 | 2068 | 2494 | 0 | 0 | 1143 | 0 | 7748 |
| Trichosporonales | 0 | 0 | 2228 | 0 | 1959 | 0 | 0 | 0 | 928 | 2412 | 0 | 625 | 0 | 0 | 4193 |
| Xylariales | 0 | 0 | 0 | 0 | 0 | 1198 | 0 | 0 | 0 | 0 | 0 | 0 | 0 | 508 | 0 |
| Unknown | 0 | 603 | 2558 | 0 | 1424 | 13226 | 24423 | 34527 | 5564 | 4716 | 4466 | 19171 | 11142 | 25500 | 6914 |

| **Family** | **ITS1_1** | **ITS1_2** | **ITS1_3** | **ITS1_4** | **ITS1_5** | **ITS1_6** | **ITS1_7** | **ITS1_8** | **ITS1_9** | **ITS1_10** | **ITS1_11** | **ITS1_12** | **ITS1_13** | **ITS1_14** | **ITS1_15** |
| --- | --- | --- | --- | --- | --- | --- | --- | --- | --- | --- | --- | --- | --- | --- | --- |
| Amanitaceae | 2749 | 0 | 0 | 0 | 0 | 0 | 0 | 0 | 2980 | 0 | 0 | 0 | 0 | 0 | 0 |
| Archaeorhizomycetaceae | 0 | 0 | 0 | 0 | 0 | 0 | 0 | 0 | 0 | 0 | 17145 | 0 | 0 | 0 | 0 |
| Ascobolaceae | 0 | 0 | 0 | 0 | 0 | 629 | 0 | 0 | 0 | 0 | 0 | 0 | 0 | 0 | 0 |
| Aspergillaceae | 0 | 0 | 0 | 0 | 0 | 0 | 0 | 0 | 0 | 0 | 0 | 0 | 0 | 0 | 838 |
| Atheliaceae | 0 | 0 | 0 | 0 | 0 | 0 | 0 | 0 | 0 | 0 | 14582 | 0 | 0 | 0 | 0 |
| Aureobasidiaceae | 0 | 0 | 0 | 0 | 0 | 0 | 0 | 1211 | 0 | 0 | 0 | 0 | 0 | 0 | 0 |
| Auriculariaceae | 0 | 0 | 0 | 0 | 0 | 0 | 0 | 0 | 0 | 0 | 0 | 0 | 840 | 0 | 0 |
| Biatriosporaceae | 0 | 0 | 572 | 0 | 0 | 746 | 0 | 0 | 0 | 0 | 0 | 0 | 0 | 0 | 0 |
| Boletaceae | 557 | 0 | 3021 | 0 | 0 | 0 | 0 | 0 | 1500 | 0 | 0 | 0 | 0 | 0 | 0 |
| Cantharellales_Incertae sedis | 0 | 0 | 0 | 0 | 0 | 0 | 0 | 0 | 0 | 1084 | 0 | 0 | 0 | 0 | 0 |
| Ceratobasidiaceae | 0 | 0 | 0 | 0 | 0 | 0 | 0 | 0 | 0 | 0 | 0 | 0 | 964 | 0 | 0 |
| Cladosporiaceae | 0 | 3041 | 0 | 16592 | 0 | 4410 | 33018 | 7214 | 0 | 663 | 2613 | 1058 | 51863 | 15546 | 24201 |
| Clavulinaceae | 0 | 0 | 0 | 0 | 0 | 0 | 0 | 0 | 0 | 0 | 1981 | 0 | 0 | 0 | 0 |
| Cortinariaceae | 0 | 9222 | 0 | 21207 | 0 | 0 | 0 | 0 | 13696 | 0 | 0 | 0 | 0 | 0 | 0 |
| Dermateaceae | 0 | 0 | 0 | 0 | 0 | 0 | 0 | 5018 | 0 | 0 | 1408 | 0 | 0 | 0 | 0 |
| Dictyosporiaceae | 0 | 0 | 0 | 0 | 0 | 0 | 0 | 0 | 0 | 0 | 0 | 0 | 0 | 1822 | 0 |
| Didymellaceae | 0 | 0 | 0 | 0 | 0 | 0 | 0 | 2119 | 0 | 0 | 0 | 0 | 0 | 26505 | 0 |
| Filobasidiaceae | 0 | 0 | 0 | 0 | 0 | 0 | 0 | 0 | 0 | 0 | 0 | 0 | 0 | 1018 | 0 |
| Ganodermataceae | 0 | 0 | 0 | 0 | 0 | 6786 | 0 | 0 | 0 | 0 | 0 | 0 | 0 | 0 | 0 |
| Gloniaceae | 4932 | 916 | 0 | 2099 | 8497 | 0 | 0 | 0 | 3946 | 4912 | 2662 | 0 | 0 | 0 | 0 |
| Helotiaceae | 0 | 0 | 0 | 0 | 0 | 0 | 0 | 0 | 0 | 2145 | 0 | 2062 | 0 | 0 | 0 |
| Helotiales_Incertae sedis | 0 | 0 | 0 | 0 | 0 | 5973 | 2167 | 536 | 0 | 0 | 1014 | 523 | 0 | 0 | 654 |
| Herpotrichiellaceae | 0 | 1429 | 0 | 1321 | 5102 | 0 | 3952 | 0 | 2617 | 0 | 0 | 0 | 5348 | 0 | 9191 |
| Hyaloriaceae | 0 | 0 | 0 | 0 | 0 | 0 | 0 | 0 | 0 | 0 | 0 | 1679 | 0 | 0 | 0 |
| Hyaloscyphaceae | 0 | 0 | 0 | 0 | 0 | 0 | 3732 | 0 | 0 | 0 | 2313 | 25540 | 0 | 0 | 0 |
| Hydnangiaceae | 0 | 2755 | 0 | 1614 | 0 | 0 | 0 | 0 | 0 | 0 | 0 | 0 | 0 | 0 | 0 |
| Hydnodontaceae | 535 | 630 | 0 | 1616 | 491 | 0 | 3257 | 0 | 659 | 1539 | 0 | 6366 | 0 | 0 | 0 |
| Hygrophoraceae | 0 | 4580 | 0 | 0 | 0 | 0 | 0 | 0 | 0 | 0 | 0 | 0 | 0 | 0 | 0 |
| Hymenogastraceae | 0 | 661 | 0 | 9026 | 0 | 4739 | 0 | 0 | 0 | 0 | 0 | 0 | 0 | 0 | 0 |
| Hyponectriaceae | 0 | 0 | 0 | 0 | 0 | 0 | 0 | 0 | 0 | 0 | 0 | 0 | 0 | 508 | 0 |
| Inocybaceae | 24369 | 0 | 0 | 745 | 26145 | 10653 | 0 | 0 | 7133 | 0 | 0 | 0 | 0 | 0 | 0 |
| Leotiaceae | 0 | 0 | 0 | 511 | 0 | 0 | 0 | 27010 | 0 | 0 | 0 | 525 | 0 | 0 | 0 |
| Leptosphaeriaceae | 0 | 0 | 0 | 0 | 0 | 0 | 0 | 0 | 0 | 0 | 0 | 0 | 0 | 1841 | 1473 |
| Massarinaceae | 0 | 0 | 0 | 0 | 0 | 965 | 0 | 0 | 0 | 0 | 0 | 0 | 0 | 0 | 0 |
| Microdochiaceae | 0 | 0 | 0 | 0 | 0 | 1198 | 0 | 0 | 0 | 0 | 0 | 0 | 0 | 0 | 0 |
| Mortierellaceae | 1682 | 3894 | 1230 | 5044 | 2511 | 10692 | 10095 | 0 | 10692 | 3563 | 1991 | 2301 | 10091 | 2872 | 10171 |
| Mrakiaceae | 0 | 0 | 0 | 0 | 0 | 2143 | 0 | 0 | 842 | 1870 | 1213 | 3539 | 0 | 0 | 0 |
| Paxillaceae | 0 | 0 | 0 | 0 | 0 | 0 | 0 | 0 | 968 | 0 | 0 | 0 | 0 | 0 | 0 |
| Pezizaceae | 0 | 0 | 0 | 0 | 0 | 680 | 0 | 0 | 0 | 0 | 0 | 0 | 0 | 0 | 0 |
| Phaeosphaeriaceae | 0 | 0 | 0 | 0 | 0 | 0 | 0 | 0 | 0 | 0 | 0 | 0 | 0 | 1977 | 0 |
| Physalacriaceae | 0 | 0 | 502 | 0 | 0 | 0 | 0 | 0 | 0 | 0 | 0 | 0 | 0 | 0 | 0 |
| Pleosporaceae | 0 | 0 | 0 | 0 | 0 | 0 | 848 | 0 | 0 | 0 | 0 | 0 | 1636 | 3649 | 0 |
| Pleosporales_Incertae sedis | 0 | 0 | 0 | 0 | 0 | 0 | 0 | 729 | 0 | 0 | 0 | 0 | 0 | 0 | 0 |
| Psathyrellaceae | 0 | 0 | 0 | 0 | 0 | 0 | 0 | 3823 | 0 | 7715 | 0 | 0 | 0 | 0 | 0 |
| Pseudeurotiaceae | 0 | 0 | 0 | 0 | 0 | 2125 | 0 | 0 | 0 | 0 | 556 | 0 | 0 | 0 | 0 |
| Pyrenochaetopsidaceae | 0 | 0 | 0 | 0 | 0 | 0 | 0 | 683 | 0 | 0 | 646 | 0 | 684 | 984 | 662 |
| Pyronemataceae | 0 | 0 | 0 | 526 | 0 | 6097 | 0 | 0 | 0 | 2324 | 5848 | 0 | 0 | 777 | 0 |
| Russulaceae | 51176 | 48462 | 0 | 4094 | 26582 | 0 | 0 | 0 | 22836 | 24144 | 0 | 1616 | 0 | 0 | 19955 |
| Russulales_Incertae sedis | 0 | 0 | 0 | 0 | 0 | 0 | 0 | 0 | 0 | 0 | 0 | 17213 | 0 | 0 | 0 |
| Sclerodermataceae | 0 | 0 | 2040 | 0 | 956 | 0 | 0 | 0 | 3858 | 0 | 0 | 0 | 0 | 0 | 0 |
| Sclerotiniaceae | 0 | 0 | 0 | 0 | 0 | 0 | 0 | 0 | 0 | 0 | 0 | 524 | 0 | 0 | 0 |
| Sebacinaceae | 0 | 0 | 0 | 0 | 0 | 0 | 0 | 0 | 0 | 0 | 2279 | 0 | 0 | 0 | 0 |
| Serendipitaceae | 0 | 0 | 0 | 0 | 0 | 915 | 0 | 0 | 0 | 1369 | 0 | 0 | 0 | 606 | 0 |
| Sporormiaceae | 0 | 0 | 0 | 0 | 0 | 2127 | 0 | 1988 | 0 | 0 | 0 | 0 | 0 | 2395 | 0 |
| Thelebolaceae | 0 | 0 | 0 | 0 | 0 | 861 | 0 | 0 | 0 | 0 | 0 | 0 | 0 | 0 | 0 |
| Thelephoraceae | 0 | 3828 | 0 | 15658 | 2113 | 10340 | 0 | 0 | 3200 | 21433 | 20180 | 0 | 741 | 0 | 0 |
| Tricholomataceae | 0 | 0 | 0 | 0 | 0 | 0 | 752 | 0 | 1883 | 0 | 0 | 0 | 0 | 0 | 0 |
| Trichosporonaceae | 0 | 0 | 2228 | 0 | 1959 | 0 | 0 | 0 | 928 | 2412 | 0 | 625 | 0 | 0 | 4193 |
| Trimorphomycetaceae | 0 | 1677 | 73277 | 605 | 3291 | 0 | 2727 | 0 | 2068 | 2494 | 0 | 0 | 1143 | 0 | 7748 |
| Tuberaceae | 0 | 0 | 0 | 645 | 0 | 0 | 0 | 0 | 0 | 0 | 0 | 0 | 0 | 0 | 0 |
| Vibrisseaceae | 0 | 0 | 0 | 0 | 0 | 0 | 0 | 0 | 0 | 0 | 1978 | 0 | 0 | 0 | 0 |
| Unknown | 0 | 4905 | 3130 | 4697 | 8353 | 13921 | 25452 | 35669 | 6194 | 8333 | 7591 | 22429 | 12690 | 25500 | 6914 |
| **Total** | **86000** | **86000** | **86000** | **86000** | **86000** | **86000** | **86000** | **86000** | **86000** | **86000** | **86000** | **86000** | **86000** | **86000** | **86000** |

| **Genus** | **ITS1_1** | **ITS1_2** | **ITS1_3** | **ITS1_4** | **ITS1_5** | **ITS1_6** | **ITS1_7** | **ITS1_8** | **ITS1_9** | **ITS1_10** | **ITS1_11** | **ITS1_12** | **ITS1_13** | **ITS1_14** | **ITS1_15** |
| --- | --- | --- | --- | --- | --- | --- | --- | --- | --- | --- | --- | --- | --- | --- | --- |
| Acephala | 0 | 0 | 0 | 0 | 0 | 0 | 0 | 0 | 0 | 0 | 1014 | 0 | 0 | 0 | 0 |
| Alatospora | 0 | 0 | 0 | 0 | 0 | 0 | 0 | 25338 | 0 | 0 | 0 | 0 | 0 | 0 | 0 |
| Alternaria | 0 | 0 | 0 | 0 | 0 | 0 | 848 | 0 | 0 | 0 | 0 | 0 | 1636 | 2159 | 0 |
| Amanita | 2749 | 0 | 0 | 0 | 0 | 0 | 0 | 0 | 2980 | 0 | 0 | 0 | 0 | 0 | 0 |
| Amphinema | 0 | 0 | 0 | 0 | 0 | 0 | 0 | 0 | 0 | 0 | 8075 | 0 | 0 | 0 | 0 |
| Apiotrichum | 0 | 0 | 1279 | 0 | 1231 | 0 | 0 | 0 | 0 | 1151 | 0 | 0 | 0 | 0 | 1134 |
| Archaeorhizomyces | 0 | 0 | 0 | 0 | 0 | 0 | 0 | 0 | 0 | 0 | 17145 | 0 | 0 | 0 | 0 |
| Armillaria | 0 | 0 | 502 | 0 | 0 | 0 | 0 | 0 | 0 | 0 | 0 | 0 | 0 | 0 | 0 |
| Ascobolus | 0 | 0 | 0 | 0 | 0 | 629 | 0 | 0 | 0 | 0 | 0 | 0 | 0 | 0 | 0 |
| Aspergillus | 0 | 0 | 0 | 0 | 0 | 0 | 0 | 0 | 0 | 0 | 0 | 0 | 0 | 0 | 838 |
| Aureobasidium | 0 | 0 | 0 | 0 | 0 | 0 | 0 | 1211 | 0 | 0 | 0 | 0 | 0 | 0 | 0 |
| Auricularia | 0 | 0 | 0 | 0 | 0 | 0 | 0 | 0 | 0 | 0 | 0 | 0 | 840 | 0 | 0 |
| Biatriospora | 0 | 0 | 572 | 0 | 0 | 746 | 0 | 0 | 0 | 0 | 0 | 0 | 0 | 0 | 0 |
| Bloxamia | 0 | 0 | 0 | 0 | 0 | 0 | 0 | 0 | 0 | 1042 | 0 | 0 | 0 | 0 | 0 |
| Boidinia | 0 | 0 | 0 | 0 | 0 | 0 | 0 | 0 | 0 | 0 | 0 | 17213 | 0 | 0 | 0 |
| Botrytis | 0 | 0 | 0 | 0 | 0 | 0 | 0 | 0 | 0 | 0 | 0 | 524 | 0 | 0 | 0 |
| Cadophora | 0 | 0 | 0 | 0 | 0 | 0 | 2167 | 0 | 0 | 0 | 0 | 0 | 0 | 0 | 0 |
| Calycina | 0 | 0 | 0 | 0 | 0 | 0 | 3732 | 0 | 0 | 0 | 0 | 0 | 0 | 0 | 0 |
| Cenococcum | 4932 | 916 | 0 | 2099 | 8497 | 0 | 0 | 0 | 3946 | 4912 | 2662 | 0 | 0 | 0 | 0 |
| Ceratobasidium | 0 | 0 | 0 | 0 | 0 | 0 | 0 | 0 | 0 | 0 | 0 | 0 | 964 | 0 | 0 |
| Cladosporium | 0 | 3041 | 0 | 16592 | 0 | 4410 | 33018 | 7214 | 0 | 663 | 2613 | 1058 | 51863 | 15546 | 24201 |
| Clavulina | 0 | 0 | 0 | 0 | 0 | 0 | 0 | 0 | 0 | 0 | 1981 | 0 | 0 | 0 | 0 |
| Cochliobolus | 0 | 0 | 0 | 0 | 0 | 0 | 0 | 0 | 0 | 0 | 0 | 0 | 0 | 1007 | 0 |
| Coprinellus | 0 | 0 | 0 | 0 | 0 | 0 | 0 | 3823 | 0 | 0 | 0 | 0 | 0 | 0 | 0 |
| Coprinopsis | 0 | 0 | 0 | 0 | 0 | 0 | 0 | 0 | 0 | 2505 | 0 | 0 | 0 | 0 | 0 |
| Cortinarius | 0 | 9222 | 0 | 21207 | 0 | 0 | 0 | 0 | 13696 | 0 | 0 | 0 | 0 | 0 | 0 |
| Cryptosporiopsis | 0 | 0 | 0 | 0 | 0 | 0 | 0 | 5018 | 0 | 0 | 869 | 0 | 0 | 0 | 0 |
| Dictyosporium | 0 | 0 | 0 | 0 | 0 | 0 | 0 | 0 | 0 | 0 | 0 | 0 | 0 | 1822 | 0 |
| Didymella | 0 | 0 | 0 | 0 | 0 | 0 | 0 | 0 | 0 | 0 | 0 | 0 | 0 | 25529 | 0 |
| Epicoccum | 0 | 0 | 0 | 0 | 0 | 0 | 0 | 0 | 0 | 0 | 0 | 0 | 0 | 976 | 0 |
| Exophiala | 0 | 1429 | 0 | 1321 | 5102 | 0 | 3952 | 0 | 2617 | 0 | 0 | 0 | 5348 | 0 | 9191 |
| Ganoderma | 0 | 0 | 0 | 0 | 0 | 6786 | 0 | 0 | 0 | 0 | 0 | 0 | 0 | 0 | 0 |
| Genabea | 0 | 0 | 0 | 0 | 0 | 6097 | 0 | 0 | 0 | 0 | 0 | 0 | 0 | 0 | 0 |
| Genea | 0 | 0 | 0 | 526 | 0 | 0 | 0 | 0 | 0 | 0 | 0 | 0 | 0 | 0 | 0 |
| Hebeloma | 0 | 0 | 0 | 3443 | 0 | 3938 | 0 | 0 | 0 | 0 | 0 | 0 | 0 | 0 | 0 |
| Hyaloscypha | 0 | 0 | 0 | 0 | 0 | 0 | 0 | 0 | 0 | 0 | 1392 | 13109 | 0 | 0 | 0 |
| Hygrocybe | 0 | 4580 | 0 | 0 | 0 | 0 | 0 | 0 | 0 | 0 | 0 | 0 | 0 | 0 | 0 |
| Hymenogaster | 0 | 661 | 0 | 4586 | 0 | 801 | 0 | 0 | 0 | 0 | 0 | 0 | 0 | 0 | 0 |
| Idriella | 0 | 0 | 0 | 0 | 0 | 1198 | 0 | 0 | 0 | 0 | 0 | 0 | 0 | 0 | 0 |
| Inocybe | 24369 | 0 | 0 | 745 | 26145 | 10653 | 0 | 0 | 7133 | 0 | 0 | 0 | 0 | 0 | 0 |
| Itersonilia | 0 | 0 | 0 | 0 | 0 | 0 | 0 | 0 | 842 | 0 | 0 | 0 | 0 | 0 | 0 |
| Juxtiphoma | 0 | 0 | 0 | 0 | 0 | 0 | 0 | 2119 | 0 | 0 | 0 | 0 | 0 | 0 | 0 |
| Laccaria | 0 | 2755 | 0 | 1614 | 0 | 0 | 0 | 0 | 0 | 0 | 0 | 0 | 0 | 0 | 0 |
| Lachnum | 0 | 0 | 0 | 0 | 0 | 0 | 0 | 0 | 0 | 0 | 921 | 0 | 0 | 0 | 0 |
| Lactarius | 38035 | 0 | 0 | 0 | 21276 | 0 | 0 | 0 | 14638 | 0 | 0 | 0 | 0 | 0 | 0 |
| Leotia | 0 | 0 | 0 | 511 | 0 | 0 | 0 | 0 | 0 | 0 | 0 | 0 | 0 | 0 | 0 |
| Leptodontidium | 0 | 0 | 0 | 0 | 0 | 0 | 0 | 0 | 0 | 0 | 0 | 0 | 0 | 0 | 654 |
| Leptosphaeria | 0 | 0 | 0 | 0 | 0 | 0 | 0 | 0 | 0 | 0 | 0 | 0 | 0 | 1841 | 1473 |
| Massarina | 0 | 0 | 0 | 0 | 0 | 965 | 0 | 0 | 0 | 0 | 0 | 0 | 0 | 0 | 0 |
| Microdochium | 0 | 0 | 0 | 0 | 0 | 0 | 0 | 0 | 0 | 0 | 0 | 0 | 0 | 508 | 0 |
| Mollisia | 0 | 0 | 0 | 0 | 0 | 0 | 0 | 0 | 0 | 0 | 539 | 0 | 0 | 0 | 0 |
| Mortierella | 1682 | 3894 | 1230 | 5044 | 2511 | 10692 | 10095 | 0 | 10692 | 3563 | 1991 | 2301 | 10091 | 2872 | 10171 |
| Mycena | 0 | 0 | 0 | 0 | 0 | 0 | 752 | 0 | 0 | 0 | 0 | 0 | 0 | 0 | 0 |
| Naganishia | 0 | 0 | 0 | 0 | 0 | 0 | 0 | 0 | 0 | 0 | 0 | 0 | 0 | 1018 | 0 |
| Naucoria | 0 | 0 | 0 | 997 | 0 | 0 | 0 | 0 | 0 | 0 | 0 | 0 | 0 | 0 | 0 |
| Neobulgaria | 0 | 0 | 0 | 0 | 0 | 0 | 0 | 1672 | 0 | 0 | 0 | 0 | 0 | 0 | 0 |
| Ochrocladosporium | 0 | 0 | 0 | 0 | 0 | 0 | 0 | 729 | 0 | 0 | 0 | 0 | 0 | 0 | 0 |
| Parasola | 0 | 0 | 0 | 0 | 0 | 0 | 0 | 0 | 0 | 4023 | 0 | 0 | 0 | 0 | 0 |
| Paxillus | 0 | 0 | 0 | 0 | 0 | 0 | 0 | 0 | 968 | 0 | 0 | 0 | 0 | 0 | 0 |
| Peziza | 0 | 0 | 0 | 0 | 0 | 680 | 0 | 0 | 0 | 0 | 0 | 0 | 0 | 0 | 0 |
| Pezoloma | 0 | 0 | 0 | 0 | 0 | 0 | 0 | 0 | 0 | 0 | 0 | 525 | 0 | 0 | 0 |
| Phialocephala | 0 | 0 | 0 | 0 | 0 | 0 | 0 | 0 | 0 | 0 | 1978 | 0 | 0 | 0 | 0 |
| Phylloporus | 0 | 0 | 3021 | 0 | 0 | 0 | 0 | 0 | 0 | 0 | 0 | 0 | 0 | 0 | 0 |
| Pithomyces | 0 | 0 | 0 | 0 | 0 | 0 | 0 | 0 | 0 | 0 | 0 | 0 | 0 | 1490 | 0 |
| Preussia | 0 | 0 | 0 | 0 | 0 | 2127 | 0 | 1988 | 0 | 0 | 0 | 0 | 0 | 2395 | 0 |
| Psathyrella | 0 | 0 | 0 | 0 | 0 | 0 | 0 | 0 | 0 | 1187 | 0 | 0 | 0 | 0 | 0 |
| Pseudeurotium | 0 | 0 | 0 | 0 | 0 | 865 | 0 | 0 | 0 | 0 | 556 | 0 | 0 | 0 | 0 |
| Pseudogymnoascus | 0 | 0 | 0 | 0 | 0 | 1260 | 0 | 0 | 0 | 0 | 0 | 0 | 0 | 0 | 0 |
| Pyrenochaetopsis | 0 | 0 | 0 | 0 | 0 | 0 | 0 | 683 | 0 | 0 | 646 | 0 | 684 | 984 | 662 |
| Russula | 13141 | 48462 | 0 | 4094 | 5306 | 0 | 0 | 0 | 8198 | 24144 | 0 | 1616 | 0 | 0 | 19955 |
| Saitozyma | 0 | 1677 | 73277 | 605 | 3291 | 0 | 2727 | 0 | 2068 | 2494 | 0 | 0 | 1143 | 0 | 7748 |
| Scleroderma | 0 | 0 | 2040 | 0 | 956 | 0 | 0 | 0 | 3858 | 0 | 0 | 0 | 0 | 0 | 0 |
| Scytalidium | 0 | 0 | 0 | 0 | 0 | 0 | 0 | 0 | 0 | 1103 | 0 | 0 | 0 | 0 | 0 |
| Sebacina | 0 | 0 | 0 | 0 | 0 | 0 | 0 | 0 | 0 | 0 | 2279 | 0 | 0 | 0 | 0 |
| Serendipita | 0 | 0 | 0 | 0 | 0 | 915 | 0 | 0 | 0 | 1369 | 0 | 0 | 0 | 606 | 0 |
| Setophoma | 0 | 0 | 0 | 0 | 0 | 0 | 0 | 0 | 0 | 0 | 0 | 0 | 0 | 970 | 0 |
| Sistotrema | 0 | 0 | 0 | 0 | 0 | 0 | 0 | 0 | 0 | 1084 | 0 | 0 | 0 | 0 | 0 |
| Stypella | 0 | 0 | 0 | 0 | 0 | 0 | 0 | 0 | 0 | 0 | 0 | 1679 | 0 | 0 | 0 |
| Subulicystidium | 0 | 0 | 0 | 0 | 0 | 0 | 0 | 0 | 0 | 1539 | 0 | 0 | 0 | 0 | 0 |
| Tausonia | 0 | 0 | 0 | 0 | 0 | 2143 | 0 | 0 | 0 | 1870 | 1213 | 3539 | 0 | 0 | 0 |
| Tetracladium | 0 | 0 | 0 | 0 | 0 | 5973 | 0 | 536 | 0 | 0 | 0 | 0 | 0 | 0 | 0 |
| Thelebolus | 0 | 0 | 0 | 0 | 0 | 861 | 0 | 0 | 0 | 0 | 0 | 0 | 0 | 0 | 0 |
| Tomentella | 0 | 3828 | 0 | 15658 | 2113 | 9430 | 0 | 0 | 3200 | 21433 | 20180 | 0 | 0 | 0 | 0 |
| Trechispora | 535 | 630 | 0 | 0 | 491 | 0 | 0 | 0 | 659 | 0 | 0 | 0 | 0 | 0 | 0 |
| Tricholoma | 0 | 0 | 0 | 0 | 0 | 0 | 0 | 0 | 1883 | 0 | 0 | 0 | 0 | 0 | 0 |
| Trichophaea | 0 | 0 | 0 | 0 | 0 | 0 | 0 | 0 | 0 | 2324 | 0 | 0 | 0 | 0 | 0 |
| Trichosporon | 0 | 0 | 949 | 0 | 728 | 0 | 0 | 0 | 928 | 1261 | 0 | 625 | 0 | 0 | 3059 |
| Tuber | 0 | 0 | 0 | 645 | 0 | 0 | 0 | 0 | 0 | 0 | 0 | 0 | 0 | 0 | 0 |
| Tylospora | 0 | 0 | 0 | 0 | 0 | 0 | 0 | 0 | 0 | 0 | 3744 | 0 | 0 | 0 | 0 |
| Varicosporium | 0 | 0 | 0 | 0 | 0 | 0 | 0 | 0 | 0 | 0 | 0 | 743 | 0 | 0 | 0 |
| Wilcoxina | 0 | 0 | 0 | 0 | 0 | 0 | 0 | 0 | 0 | 0 | 5848 | 0 | 0 | 0 | 0 |
| Xenopolyscytalum | 0 | 0 | 0 | 0 | 0 | 0 | 0 | 0 | 0 | 0 | 0 | 523 | 0 | 0 | 0 |
| Xerocomellus | 557 | 0 | 0 | 0 | 0 | 0 | 0 | 0 | 0 | 0 | 0 | 0 | 0 | 0 | 0 |
| Xerocomus | 0 | 0 | 0 | 0 | 0 | 0 | 0 | 0 | 1500 | 0 | 0 | 0 | 0 | 0 | 0 |
| Unknown | 0 | 4905 | 3130 | 6313 | 8353 | 14831 | 28709 | 35669 | 6194 | 8333 | 10354 | 42545 | 13431 | 26277 | 6914 |
| **Total** | **86000** | **86000** | **86000** | **86000** | **86000** | **86000** | **86000** | **86000** | **86000** | **86000** | **86000** | **86000** | **86000** | **86000** | **86000** |

| **Phylum** | **ITS2_1** | **ITS2_2** | **ITS2_3** | **ITS2_4** | **ITS2_5** | **ITS2_6** | **ITS2_7** | **ITS2_8** | **ITS2_9** | **ITS2_10** | **ITS2_11** | **ITS2_12** | **ITS2_13** | **ITS2_14** | **ITS2_15** |
| --- | --- | --- | --- | --- | --- | --- | --- | --- | --- | --- | --- | --- | --- | --- | --- |
| Ascomycota | 11096 | 45814 | 45652 | 62277 | 56464 | 91555 | 108454 | 113091 | 14593 | 48180 | 59202 | 65770 | 114293 | 112283 | 80765 |
| Basidiomycota | 102068 | 69150 | 62641 | 53723 | 56648 | 22215 | 7546 | 2909 | 90970 | 67820 | 54037 | 46611 | 785 | 0 | 30732 |
| Mucoromycota | 2836 | 0 | 6283 | 0 | 0 | 2230 | 0 | 0 | 10437 | 0 | 1868 | 0 | 0 | 0 | 1891 |
| Unknown | 0 | 1036 | 1424 | 0 | 2888 | 0 | 0 | 0 | 0 | 0 | 893 | 3619 | 922 | 3717 | 2612 |
| **Total** | **116000** | **116000** | **116000** | **116000** | **116000** | **116000** | **116000** | **116000** | **116000** | **116000** | **116000** | **116000** | **116000** | **116000** | **116000** |

| **Class** | **ITS2_1** | **ITS2_2** | **ITS2_3** | **ITS2_4** | **ITS2_5** | **ITS2_6** | **ITS2_7** | **ITS2_8** | **ITS2_9** | **ITS2_10** | **ITS2_11** | **ITS2_12** | **ITS2_13** | **ITS2_14** | **ITS2_15** |
| --- | --- | --- | --- | --- | --- | --- | --- | --- | --- | --- | --- | --- | --- | --- | --- |
| Agaricomycetes | 101302 | 62205 | 25912 | 53723 | 39258 | 13690 | 6327 | 2909 | 79751 | 25998 | 48523 | 38226 | 0 | 0 | 7518 |
| Ascomycota_Incertae sedis | 0 | 0 | 0 | 0 | 0 | 2654 | 0 | 0 | 0 | 2809 | 0 | 0 | 0 | 0 | 0 |
| Dothideomycetes | 8039 | 25943 | 9176 | 56995 | 22043 | 30299 | 84764 | 61047 | 5527 | 14178 | 18140 | 3916 | 101911 | 68135 | 55364 |
| Eurotiomycetes | 2320 | 0 | 5536 | 0 | 0 | 0 | 0 | 11687 | 1239 | 0 | 0 | 0 | 0 | 0 | 9790 |
| Leotiomycetes | 737 | 11275 | 16018 | 798 | 2552 | 24389 | 7067 | 34331 | 4785 | 3675 | 15984 | 61854 | 2848 | 1286 | 1845 |
| Mortierellomycetes | 2836 | 0 | 6283 | 0 | 0 | 2230 | 0 | 0 | 10437 | 0 | 1868 | 0 | 0 | 0 | 1891 |
| Pezizomycetes | 0 | 1518 | 0 | 1012 | 0 | 6755 | 0 | 0 | 0 | 4476 | 20620 | 0 | 0 | 0 | 0 |
| Saccharomycetes | 0 | 0 | 0 | 0 | 0 | 1195 | 0 | 0 | 0 | 0 | 0 | 0 | 0 | 0 | 0 |
| Sordariomycetes | 0 | 4696 | 7798 | 3472 | 3600 | 22099 | 16623 | 1685 | 3042 | 21798 | 2861 | 0 | 9534 | 42862 | 10865 |
| Tremellomycetes | 766 | 6945 | 35395 | 0 | 17390 | 8525 | 0 | 0 | 11219 | 41822 | 5514 | 8385 | 785 | 0 | 22030 |
| Unknown | 0 | 3418 | 9882 | 0 | 31157 | 4164 | 1219 | 4341 | 0 | 1244 | 2490 | 3619 | 922 | 3717 | 6697 |
| **Total** | **116000** | **116000** | **116000** | **116000** | **116000** | **116000** | **116000** | **116000** | **116000** | **116000** | **116000** | **116000** | **116000** | **116000** | **116000** |

| **Order** | **ITS2_1** | **ITS2_2** | **ITS2_3** | **ITS2_4** | **ITS2_5** | **ITS2_6** | **ITS2_7** | **ITS2_8** | **ITS2_9** | **ITS2_10** | **ITS2_11** | **ITS2_12** | **ITS2_13** | **ITS2_14** | **ITS2_15** |
| --- | --- | --- | --- | --- | --- | --- | --- | --- | --- | --- | --- | --- | --- | --- | --- |
| Agaricales | 64345 | 18803 | 894 | 31195 | 23744 | 2947 | 4622 | 2909 | 39345 | 8757 | 1697 | 4055 | 0 | 0 | 0 |
| Ascomycota_Incertae sedis | 0 | 0 | 0 | 0 | 0 | 2654 | 0 | 0 | 0 | 2809 | 0 | 0 | 0 | 0 | 0 |
| Atheliales | 0 | 0 | 0 | 0 | 0 | 0 | 0 | 0 | 0 | 0 | 19528 | 0 | 0 | 0 | 0 |
| Auriculariales | 0 | 845 | 4717 | 6704 | 1848 | 0 | 0 | 0 | 1514 | 876 | 1184 | 7855 | 0 | 0 | 0 |
| Boletales | 0 | 2107 | 8772 | 1112 | 895 | 0 | 0 | 0 | 5087 | 0 | 0 | 0 | 0 | 0 | 0 |
| Cantharellales | 719 | 0 | 0 | 0 | 3701 | 0 | 0 | 0 | 1016 | 0 | 5663 | 0 | 0 | 0 | 0 |
| Capnodiales | 0 | 14477 | 3977 | 49628 | 676 | 9573 | 79827 | 7669 | 1227 | 1762 | 8630 | 2898 | 83303 | 25469 | 47624 |
| Chaetothyriales | 0 | 0 | 5536 | 0 | 0 | 0 | 0 | 11687 | 1239 | 0 | 0 | 0 | 0 | 0 | 8930 |
| Coniochaetales | 0 | 0 | 0 | 0 | 0 | 0 | 0 | 0 | 0 | 3076 | 0 | 0 | 1662 | 0 | 0 |
| Cystofilobasidiales | 0 | 0 | 1643 | 0 | 2572 | 3126 | 0 | 0 | 950 | 1555 | 5514 | 6069 | 0 | 0 | 5371 |
| Diaporthales | 0 | 0 | 0 | 0 | 0 | 1924 | 0 | 0 | 0 | 0 | 0 | 0 | 0 | 0 | 0 |
| Dothideales | 0 | 0 | 0 | 0 | 0 | 0 | 0 | 4999 | 0 | 0 | 0 | 0 | 0 | 0 | 0 |
| Eurotiales | 2320 | 0 | 0 | 0 | 0 | 0 | 0 | 0 | 0 | 0 | 0 | 0 | 0 | 0 | 0 |
| Filobasidiales | 0 | 0 | 15457 | 0 | 0 | 0 | 0 | 0 | 914 | 0 | 0 | 0 | 0 | 0 | 3102 |
| Geastrales | 0 | 2730 | 0 | 0 | 0 | 0 | 0 | 0 | 0 | 0 | 0 | 0 | 0 | 0 | 0 |
| Helotiales | 737 | 6975 | 16018 | 0 | 2552 | 22273 | 7067 | 34331 | 4785 | 3675 | 15103 | 61854 | 2848 | 1286 | 1845 |
| Hymenochaetales | 0 | 0 | 0 | 0 | 0 | 1780 | 0 | 0 | 0 | 0 | 0 | 802 | 0 | 0 | 0 |
| Hypocreales | 0 | 1171 | 2126 | 3472 | 0 | 3659 | 16623 | 0 | 832 | 0 | 2861 | 0 | 7181 | 8005 | 8240 |
| Mortierellales | 2836 | 0 | 6283 | 0 | 0 | 2230 | 0 | 0 | 10437 | 0 | 1868 | 0 | 0 | 0 | 1891 |
| Mytilinidiales | 8039 | 2365 | 0 | 2657 | 16644 | 0 | 0 | 0 | 4300 | 4134 | 2097 | 0 | 0 | 0 | 0 |
| Onygenales | 0 | 0 | 0 | 0 | 0 | 0 | 0 | 0 | 0 | 0 | 0 | 0 | 0 | 0 | 860 |
| Pezizales | 0 | 1518 | 0 | 1012 | 0 | 6755 | 0 | 0 | 0 | 4476 | 20620 | 0 | 0 | 0 | 0 |
| Pleosporales | 0 | 7735 | 3669 | 4710 | 4723 | 17925 | 4937 | 48379 | 0 | 7189 | 7413 | 0 | 18608 | 42666 | 7740 |
| Polyporales | 0 | 0 | 0 | 0 | 0 | 4267 | 0 | 0 | 0 | 0 | 0 | 0 | 0 | 0 | 0 |
| Russulales | 32570 | 33756 | 1824 | 3513 | 4978 | 0 | 0 | 0 | 22235 | 10884 | 0 | 15260 | 0 | 0 | 7518 |
| Saccharomycetales | 0 | 0 | 0 | 0 | 0 | 1195 | 0 | 0 | 0 | 0 | 0 | 0 | 0 | 0 | 0 |
| Sebacinales | 0 | 0 | 0 | 0 | 0 | 876 | 0 | 0 | 0 | 0 | 0 | 925 | 0 | 0 | 0 |
| Sordariales | 0 | 3525 | 5672 | 0 | 3600 | 8310 | 0 | 1685 | 816 | 18722 | 0 | 0 | 691 | 33416 | 1698 |
| Sordariomycetes_Incertae sedis | 0 | 0 | 0 | 0 | 0 | 0 | 0 | 0 | 0 | 0 | 0 | 0 | 0 | 1441 | 0 |
| Thelebolales | 0 | 0 | 0 | 0 | 0 | 2116 | 0 | 0 | 0 | 0 | 0 | 0 | 0 | 0 | 0 |
| Thelephorales | 0 | 3964 | 0 | 11199 | 1016 | 3820 | 0 | 0 | 4770 | 5481 | 15716 | 0 | 0 | 0 | 0 |
| Trechisporales | 3668 | 0 | 0 | 0 | 1698 | 0 | 0 | 0 | 5784 | 0 | 2115 | 0 | 0 | 0 | 0 |
| Tremellales | 0 | 1490 | 9704 | 0 | 3217 | 0 | 0 | 0 | 2217 | 1196 | 0 | 0 | 0 | 0 | 4684 |
| Trichosporonales | 766 | 5455 | 8591 | 0 | 11601 | 5399 | 0 | 0 | 7138 | 39071 | 0 | 2316 | 785 | 0 | 8873 |
| Venturiales | 0 | 0 | 1530 | 0 | 0 | 0 | 0 | 0 | 0 | 0 | 0 | 0 | 0 | 0 | 0 |
| Xylariales | 0 | 0 | 0 | 0 | 0 | 8206 | 0 | 0 | 0 | 0 | 0 | 0 | 0 | 0 | 927 |
| Unknown | 0 | 9084 | 19587 | 798 | 32535 | 6965 | 2924 | 4341 | 1394 | 2337 | 5991 | 13966 | 922 | 3717 | 6697 |
| **Total** | **116000** | **116000** | **116000** | **116000** | **116000** | **116000** | **116000** | **116000** | **116000** | **116000** | **116000** | **116000** | **116000** | **116000** | **116000** |

| **Family** | **ITS2_1** | **ITS2_2** | **ITS2_3** | **ITS2_4** | **ITS2_5** | **ITS2_6** | **ITS2_7** | **ITS2_8** | **ITS2_9** | **ITS2_10** | **ITS2_11** | **ITS2_12** | **ITS2_13** | **ITS2_14** | **ITS2_15** |
| --- | --- | --- | --- | --- | --- | --- | --- | --- | --- | --- | --- | --- | --- | --- | --- |
| Agaricaceae | 0 | 0 | 0 | 0 | 0 | 0 | 0 | 0 | 0 | 888 | 0 | 0 | 0 | 0 | 0 |
| Amanitaceae | 28785 | 0 | 0 | 0 | 0 | 0 | 0 | 0 | 8394 | 0 | 0 | 0 | 0 | 0 | 0 |
| Amniculicolaceae | 0 | 0 | 0 | 0 | 0 | 0 | 0 | 2609 | 0 | 0 | 0 | 0 | 0 | 0 | 0 |
| Antennulariellaceae | 0 | 0 | 0 | 0 | 0 | 0 | 3377 | 0 | 0 | 0 | 0 | 0 | 3207 | 0 | 791 |
| Ascocorticiaceae | 0 | 0 | 0 | 0 | 0 | 0 | 0 | 0 | 0 | 0 | 0 | 11483 | 0 | 0 | 0 |
| Ascodesmidaceae | 0 | 0 | 0 | 0 | 0 | 932 | 0 | 0 | 0 | 0 | 0 | 0 | 0 | 0 | 0 |
| Atheliaceae | 0 | 0 | 0 | 0 | 0 | 0 | 0 | 0 | 0 | 0 | 19528 | 0 | 0 | 0 | 0 |
| Aureobasidiaceae | 0 | 0 | 0 | 0 | 0 | 0 | 0 | 1500 | 0 | 0 | 0 | 0 | 0 | 0 | 0 |
| Biatriosporaceae | 0 | 1130 | 899 | 0 | 0 | 3298 | 0 | 0 | 0 | 0 | 0 | 0 | 0 | 0 | 0 |
| Bionectriaceae | 0 | 0 | 0 | 0 | 0 | 0 | 0 | 0 | 0 | 0 | 0 | 0 | 0 | 0 | 2523 |
| Boletaceae | 0 | 0 | 0 | 0 | 0 | 0 | 0 | 0 | 3085 | 0 | 0 | 0 | 0 | 0 | 0 |
| Ceratobasidiaceae | 719 | 0 | 0 | 0 | 3701 | 0 | 0 | 0 | 1016 | 0 | 0 | 0 | 0 | 0 | 0 |
| Chaetomiaceae | 0 | 1885 | 4885 | 0 | 3600 | 0 | 0 | 0 | 816 | 1307 | 0 | 0 | 691 | 0 | 1698 |
| Cladosporiaceae | 0 | 14477 | 3159 | 49628 | 676 | 9573 | 76450 | 7669 | 1227 | 1762 | 8630 | 2898 | 80096 | 25469 | 46833 |
| Clavulinaceae | 0 | 0 | 0 | 0 | 0 | 0 | 0 | 0 | 0 | 0 | 5663 | 0 | 0 | 0 | 0 |
| Coniochaetaceae | 0 | 0 | 0 | 0 | 0 | 0 | 0 | 0 | 0 | 3076 | 0 | 0 | 1662 | 0 | 0 |
| Coprinaceae | 0 | 0 | 0 | 0 | 0 | 0 | 0 | 781 | 0 | 0 | 0 | 0 | 0 | 0 | 0 |
| Cortinariaceae | 1060 | 13632 | 0 | 16336 | 0 | 0 | 0 | 0 | 16832 | 0 | 0 | 0 | 0 | 0 | 0 |
| Debaryomycetaceae | 0 | 0 | 0 | 0 | 0 | 1195 | 0 | 0 | 0 | 0 | 0 | 0 | 0 | 0 | 0 |
| Dermateaceae | 0 | 1232 | 0 | 0 | 0 | 0 | 0 | 6141 | 0 | 0 | 1318 | 0 | 0 | 0 | 0 |
| Dictyosporiaceae | 0 | 0 | 0 | 0 | 0 | 0 | 0 | 0 | 0 | 0 | 0 | 0 | 0 | 1799 | 0 |
| Didymellaceae | 0 | 0 | 1629 | 742 | 0 | 1734 | 0 | 3551 | 0 | 0 | 986 | 0 | 0 | 23966 | 875 |
| Didymosphaeriaceae | 0 | 0 | 0 | 0 | 0 | 0 | 0 | 0 | 0 | 0 | 0 | 0 | 0 | 0 | 1143 |
| Dothioraceae | 0 | 0 | 0 | 0 | 0 | 0 | 0 | 3499 | 0 | 0 | 0 | 0 | 0 | 0 | 0 |
| Elaphomycetaceae | 2320 | 0 | 0 | 0 | 0 | 0 | 0 | 0 | 0 | 0 | 0 | 0 | 0 | 0 | 0 |
| Ganodermataceae | 0 | 0 | 0 | 0 | 0 | 4267 | 0 | 0 | 0 | 0 | 0 | 0 | 0 | 0 | 0 |
| Geastraceae | 0 | 2730 | 0 | 0 | 0 | 0 | 0 | 0 | 0 | 0 | 0 | 0 | 0 | 0 | 0 |
| Gloniaceae | 8039 | 2365 | 0 | 2657 | 16644 | 0 | 0 | 0 | 4300 | 4134 | 2097 | 0 | 0 | 0 | 0 |
| Helotiaceae | 737 | 1138 | 1067 | 0 | 0 | 5399 | 0 | 4223 | 1657 | 2709 | 0 | 2187 | 0 | 0 | 0 |
| Helotiales_Incertae sedis | 0 | 0 | 4209 | 0 | 0 | 11326 | 4622 | 1188 | 1645 | 0 | 8822 | 1198 | 2848 | 1286 | 1845 |
| Herpotrichiellaceae | 0 | 0 | 2910 | 0 | 0 | 0 | 0 | 11687 | 1239 | 0 | 0 | 0 | 0 | 0 | 0 |
| Hyaloriaceae | 0 | 0 | 0 | 0 | 0 | 0 | 0 | 0 | 0 | 0 | 0 | 7026 | 0 | 0 | 0 |
| Hyaloscyphaceae | 0 | 0 | 0 | 0 | 0 | 0 | 0 | 0 | 0 | 0 | 1117 | 9392 | 0 | 0 | 0 |
| Hydnangiaceae | 2213 | 5171 | 0 | 4397 | 0 | 0 | 0 | 0 | 0 | 0 | 0 | 0 | 0 | 0 | 0 |
| Hydnodontaceae | 3668 | 0 | 0 | 0 | 0 | 0 | 0 | 0 | 907 | 0 | 1112 | 0 | 0 | 0 | 0 |
| Hymenogastraceae | 0 | 0 | 0 | 8631 | 0 | 0 | 0 | 0 | 0 | 0 | 0 | 0 | 0 | 0 | 0 |
| Incertae sedis | 0 | 0 | 0 | 0 | 0 | 2654 | 0 | 0 | 0 | 2809 | 0 | 0 | 0 | 0 | 0 |
| Inocybaceae | 32287 | 0 | 0 | 1831 | 23744 | 1931 | 0 | 0 | 9707 | 0 | 0 | 0 | 0 | 0 | 0 |
| Lasiosphaeriaceae | 0 | 1640 | 0 | 0 | 0 | 2844 | 0 | 1685 | 0 | 10795 | 0 | 0 | 0 | 0 | 0 |
| Leotiaceae | 0 | 0 | 0 | 0 | 0 | 0 | 0 | 18730 | 0 | 0 | 1119 | 856 | 0 | 0 | 0 |
| Lindgomycetaceae | 0 | 0 | 0 | 0 | 0 | 0 | 0 | 0 | 0 | 0 | 972 | 0 | 0 | 5544 | 0 |
| Lophiostomataceae | 0 | 0 | 0 | 0 | 0 | 0 | 0 | 0 | 0 | 913 | 0 | 0 | 0 | 0 | 0 |
| Melanconidaceae | 0 | 0 | 0 | 0 | 0 | 1924 | 0 | 0 | 0 | 0 | 0 | 0 | 0 | 0 | 0 |
| Melanogastraceae | 0 | 0 | 890 | 0 | 0 | 0 | 0 | 0 | 0 | 0 | 0 | 0 | 0 | 0 | 0 |
| Microdochiaceae | 0 | 0 | 0 | 0 | 0 | 8206 | 0 | 0 | 0 | 0 | 0 | 0 | 0 | 0 | 0 |
| Mortierellaceae | 2836 | 0 | 6283 | 0 | 0 | 2230 | 0 | 0 | 10437 | 0 | 1868 | 0 | 0 | 0 | 1891 |
| Mrakiaceae | 0 | 0 | 1643 | 0 | 2572 | 3126 | 0 | 0 | 950 | 1555 | 5514 | 6069 | 0 | 0 | 5371 |
| Myxotrichaceae | 0 | 0 | 0 | 0 | 0 | 4673 | 0 | 4049 | 0 | 0 | 0 | 0 | 0 | 0 | 0 |
| Nectriaceae | 0 | 1171 | 2126 | 3472 | 0 | 1506 | 16623 | 0 | 832 | 0 | 2861 | 0 | 7181 | 8005 | 4546 |
| Onygenales_Incertae sedis | 0 | 0 | 0 | 0 | 0 | 0 | 0 | 0 | 0 | 0 | 0 | 0 | 0 | 0 | 860 |
| Ophiocordycipitaceae | 0 | 0 | 0 | 0 | 0 | 0 | 0 | 0 | 0 | 0 | 0 | 0 | 0 | 0 | 1171 |
| Paxillaceae | 0 | 0 | 0 | 0 | 0 | 0 | 0 | 0 | 2002 | 0 | 0 | 0 | 0 | 0 | 0 |
| Pezizaceae | 0 | 0 | 0 | 0 | 0 | 5823 | 0 | 0 | 0 | 0 | 0 | 0 | 0 | 0 | 0 |
| Phaeosphaeriaceae | 0 | 0 | 0 | 0 | 0 | 0 | 0 | 0 | 0 | 0 | 0 | 0 | 0 | 1695 | 0 |
| Piskurozymaceae | 0 | 0 | 15457 | 0 | 0 | 0 | 0 | 0 | 914 | 0 | 0 | 0 | 0 | 0 | 3102 |
| Pleosporaceae | 0 | 0 | 0 | 0 | 0 | 0 | 2652 | 0 | 0 | 0 | 0 | 0 | 5243 | 7275 | 0 |
| Pleosporales_Incertae sedis | 0 | 0 | 0 | 0 | 0 | 0 | 0 | 1792 | 0 | 0 | 0 | 0 | 868 | 0 | 0 |
| Psathyrellaceae | 0 | 0 | 0 | 0 | 0 | 1016 | 0 | 2128 | 0 | 7869 | 0 | 0 | 0 | 0 | 0 |
| Pyrenochaetopsidaceae | 0 | 3114 | 0 | 1484 | 764 | 3495 | 0 | 3426 | 0 | 2189 | 1691 | 0 | 1444 | 1233 | 4286 |
| Pyronemataceae | 0 | 1518 | 0 | 0 | 0 | 0 | 0 | 0 | 0 | 3613 | 20620 | 0 | 0 | 0 | 0 |
| Russulaceae | 32570 | 33756 | 1824 | 3513 | 4978 | 0 | 0 | 0 | 22235 | 10884 | 0 | 1925 | 0 | 0 | 7518 |
| Russulales_Incertae sedis | 0 | 0 | 0 | 0 | 0 | 0 | 0 | 0 | 0 | 0 | 0 | 12222 | 0 | 0 | 0 |
| Schizoporaceae | 0 | 0 | 0 | 0 | 0 | 1780 | 0 | 0 | 0 | 0 | 0 | 0 | 0 | 0 | 0 |
| Sclerodermataceae | 0 | 2107 | 7882 | 1112 | 895 | 0 | 0 | 0 | 0 | 0 | 0 | 0 | 0 | 0 | 0 |
| Sclerotiniaceae | 0 | 0 | 0 | 0 | 0 | 0 | 0 | 0 | 0 | 0 | 0 | 1628 | 0 | 0 | 0 |
| Serendipitaceae | 0 | 0 | 0 | 0 | 0 | 876 | 0 | 0 | 0 | 0 | 0 | 0 | 0 | 0 | 0 |
| Sordariaceae | 0 | 0 | 0 | 0 | 0 | 4393 | 0 | 0 | 0 | 0 | 0 | 0 | 0 | 20944 | 0 |
| Sordariales_Incertae sedis | 0 | 0 | 0 | 0 | 0 | 0 | 0 | 0 | 0 | 5553 | 0 | 0 | 0 | 8041 | 0 |
| Sordariomycetes_Incertae sedis | 0 | 0 | 0 | 0 | 0 | 0 | 0 | 0 | 0 | 0 | 0 | 0 | 0 | 1441 | 0 |
| Sporormiaceae | 0 | 1396 | 0 | 0 | 0 | 994 | 0 | 821 | 0 | 0 | 0 | 0 | 0 | 0 | 0 |
| Tetragoniomycetaceae | 0 | 0 | 0 | 0 | 0 | 0 | 0 | 0 | 0 | 0 | 0 | 0 | 785 | 0 | 0 |
| Thelebolaceae | 0 | 0 | 0 | 0 | 0 | 2116 | 0 | 0 | 0 | 0 | 0 | 0 | 0 | 0 | 0 |
| Thelephoraceae | 0 | 3964 | 0 | 11199 | 1016 | 3820 | 0 | 0 | 4770 | 5481 | 15716 | 0 | 0 | 0 | 0 |
| Thyridariaceae | 0 | 0 | 0 | 0 | 0 | 3628 | 0 | 0 | 0 | 0 | 0 | 0 | 0 | 0 | 0 |
| Trematosphaeriaceae | 0 | 1253 | 0 | 2484 | 0 | 0 | 2285 | 0 | 0 | 0 | 1916 | 0 | 8652 | 0 | 1436 |
| Tricholomataceae | 0 | 0 | 894 | 0 | 0 | 0 | 3790 | 0 | 4412 | 0 | 1697 | 4055 | 0 | 0 | 0 |
| Trichosporonaceae | 766 | 5455 | 8591 | 0 | 11601 | 5399 | 0 | 0 | 7138 | 39071 | 0 | 2316 | 0 | 0 | 8873 |
| Trimorphomycetaceae | 0 | 1490 | 9704 | 0 | 3217 | 0 | 0 | 0 | 2217 | 1196 | 0 | 0 | 0 | 0 | 4684 |
| Tuberaceae | 0 | 0 | 0 | 1012 | 0 | 0 | 0 | 0 | 0 | 863 | 0 | 0 | 0 | 0 | 0 |
| Venturiaceae | 0 | 0 | 1530 | 0 | 0 | 0 | 0 | 0 | 0 | 0 | 0 | 0 | 0 | 0 | 0 |
| Xylariaceae | 0 | 0 | 0 | 0 | 0 | 0 | 0 | 0 | 0 | 0 | 0 | 0 | 0 | 0 | 927 |
| Unknown | 0 | 15376 | 40418 | 7502 | 42592 | 15842 | 6201 | 40521 | 9268 | 9333 | 12753 | 52745 | 3323 | 9302 | 15627 |
| **Total** | **116000** | **116000** | **116000** | **116000** | **116000** | **116000** | **116000** | **116000** | **116000** | **116000** | **116000** | **116000** | **116000** | **116000** | **116000** |

| **Genus** | **ITS2_1** | **ITS2_2** | **ITS2_3** | **ITS2_4** | **ITS2_5** | **ITS2_6** | **ITS2_7** | **ITS2_8** | **ITS2_9** | **ITS2_10** | **ITS2_11** | **ITS2_12** | **ITS2_13** | **ITS2_14** | **ITS2_15** |
| --- | --- | --- | --- | --- | --- | --- | --- | --- | --- | --- | --- | --- | --- | --- | --- |
| Agaricus | 0 | 0 | 0 | 0 | 0 | 0 | 0 | 0 | 0 | 888 | 0 | 0 | 0 | 0 | 0 |
| Alatospora | 0 | 0 | 0 | 0 | 0 | 0 | 0 | 18730 | 0 | 0 | 1119 | 856 | 0 | 0 | 0 |
| Alternaria | 0 | 0 | 0 | 0 | 0 | 0 | 2652 | 0 | 0 | 0 | 0 | 0 | 5243 | 3668 | 0 |
| Amanita | 28785 | 0 | 0 | 0 | 0 | 0 | 0 | 0 | 8394 | 0 | 0 | 0 | 0 | 0 | 0 |
| Amphinema | 0 | 0 | 0 | 0 | 0 | 0 | 0 | 0 | 0 | 0 | 8152 | 0 | 0 | 0 | 0 |
| Anguillospora | 0 | 0 | 0 | 0 | 0 | 5399 | 0 | 2577 | 0 | 0 | 0 | 0 | 0 | 0 | 0 |
| Antennariella | 0 | 0 | 0 | 0 | 0 | 0 | 3377 | 0 | 0 | 0 | 0 | 0 | 3207 | 0 | 791 |
| Apiotrichum | 0 | 5455 | 8591 | 0 | 11601 | 5399 | 0 | 0 | 7138 | 39071 | 0 | 2316 | 0 | 0 | 8873 |
| Apodus | 0 | 1640 | 0 | 0 | 0 | 1240 | 0 | 0 | 0 | 2537 | 0 | 0 | 0 | 0 | 0 |
| Ascocorticium | 0 | 0 | 0 | 0 | 0 | 0 | 0 | 0 | 0 | 0 | 0 | 11483 | 0 | 0 | 0 |
| Aureobasidium | 0 | 0 | 0 | 0 | 0 | 0 | 0 | 1500 | 0 | 0 | 0 | 0 | 0 | 0 | 0 |
| Biatriospora | 0 | 1130 | 899 | 0 | 0 | 3298 | 0 | 0 | 0 | 0 | 0 | 0 | 0 | 0 | 0 |
| Bipolaris | 0 | 0 | 0 | 0 | 0 | 0 | 0 | 0 | 0 | 0 | 0 | 0 | 0 | 1638 | 0 |
| Bloxamia | 0 | 0 | 0 | 0 | 0 | 0 | 0 | 0 | 0 | 1470 | 0 | 0 | 0 | 0 | 0 |
| Boidinia | 0 | 0 | 0 | 0 | 0 | 0 | 0 | 0 | 0 | 0 | 0 | 12222 | 0 | 0 | 0 |
| Botrytis | 0 | 0 | 0 | 0 | 0 | 0 | 0 | 0 | 0 | 0 | 0 | 1628 | 0 | 0 | 0 |
| Brevicellicium | 0 | 0 | 0 | 0 | 0 | 0 | 0 | 0 | 0 | 0 | 1112 | 0 | 0 | 0 | 0 |
| Cadophora | 0 | 0 | 0 | 0 | 0 | 0 | 1961 | 0 | 0 | 0 | 8822 | 0 | 2848 | 1286 | 0 |
| Cenococcum | 8039 | 2365 | 0 | 2657 | 16644 | 0 | 0 | 0 | 4300 | 4134 | 2097 | 0 | 0 | 0 | 0 |
| Cephaliophora | 0 | 0 | 0 | 0 | 0 | 932 | 0 | 0 | 0 | 0 | 0 | 0 | 0 | 0 | 0 |
| Chaetomium | 0 | 0 | 2668 | 0 | 2143 | 0 | 0 | 0 | 0 | 0 | 0 | 0 | 0 | 0 | 841 |
| Chalara | 0 | 0 | 0 | 0 | 0 | 0 | 2661 | 0 | 0 | 0 | 0 | 0 | 0 | 0 | 0 |
| Chrysosporium | 0 | 0 | 0 | 0 | 0 | 0 | 0 | 0 | 0 | 0 | 0 | 0 | 0 | 0 | 860 |
| Cladosporium | 0 | 14477 | 3159 | 49628 | 676 | 9573 | 76450 | 7669 | 1227 | 1762 | 8630 | 2898 | 80096 | 25469 | 46833 |
| Clavulina | 0 | 0 | 0 | 0 | 0 | 0 | 0 | 0 | 0 | 0 | 5663 | 0 | 0 | 0 | 0 |
| Clitocybe | 0 | 0 | 894 | 0 | 0 | 0 | 0 | 0 | 1826 | 0 | 0 | 0 | 0 | 0 | 0 |
| Clohesyomyces | 0 | 0 | 0 | 0 | 0 | 0 | 0 | 0 | 0 | 0 | 972 | 0 | 0 | 1865 | 0 |
| Clonostachys | 0 | 0 | 0 | 0 | 0 | 0 | 0 | 0 | 0 | 0 | 0 | 0 | 0 | 0 | 2523 |
| Coniochaeta | 0 | 0 | 0 | 0 | 0 | 0 | 0 | 0 | 0 | 3076 | 0 | 0 | 0 | 0 | 0 |
| Coprinellus | 0 | 0 | 0 | 0 | 0 | 1016 | 0 | 2128 | 0 | 0 | 0 | 0 | 0 | 0 | 0 |
| Coprinopsis | 0 | 0 | 0 | 0 | 0 | 0 | 0 | 0 | 0 | 3273 | 0 | 0 | 0 | 0 | 0 |
| Coprinus | 0 | 0 | 0 | 0 | 0 | 0 | 0 | 781 | 0 | 0 | 0 | 0 | 0 | 0 | 0 |
| Cortinarius | 1060 | 13632 | 0 | 16336 | 0 | 0 | 0 | 0 | 16832 | 0 | 0 | 0 | 0 | 0 | 0 |
| Cuspidatispora | 0 | 0 | 0 | 0 | 0 | 1604 | 0 | 0 | 0 | 0 | 0 | 0 | 0 | 0 | 0 |
| Debaryomyces | 0 | 0 | 0 | 0 | 0 | 1195 | 0 | 0 | 0 | 0 | 0 | 0 | 0 | 0 | 0 |
| Dictyosporium | 0 | 0 | 0 | 0 | 0 | 0 | 0 | 0 | 0 | 0 | 0 | 0 | 0 | 1799 | 0 |
| Elaphomyces | 2320 | 0 | 0 | 0 | 0 | 0 | 0 | 0 | 0 | 0 | 0 | 0 | 0 | 0 | 0 |
| Epicoccum | 0 | 0 | 1629 | 742 | 0 | 1734 | 0 | 3551 | 0 | 0 | 0 | 0 | 0 | 23966 | 875 |
| Exophiala | 0 | 0 | 2910 | 0 | 0 | 0 | 0 | 0 | 1239 | 0 | 0 | 0 | 0 | 0 | 0 |
| Fusarium | 0 | 1171 | 0 | 3472 | 0 | 0 | 16623 | 0 | 0 | 0 | 2861 | 0 | 7181 | 7091 | 4546 |
| Ganoderma | 0 | 0 | 0 | 0 | 0 | 4267 | 0 | 0 | 0 | 0 | 0 | 0 | 0 | 0 | 0 |
| Geastrum | 0 | 2730 | 0 | 0 | 0 | 0 | 0 | 0 | 0 | 0 | 0 | 0 | 0 | 0 | 0 |
| Gibberella | 0 | 0 | 0 | 0 | 0 | 0 | 0 | 0 | 0 | 0 | 0 | 0 | 0 | 914 | 0 |
| Hormiactis | 0 | 0 | 0 | 0 | 0 | 2654 | 0 | 0 | 0 | 0 | 0 | 0 | 0 | 0 | 0 |
| Hormonema | 0 | 0 | 0 | 0 | 0 | 0 | 0 | 3499 | 0 | 0 | 0 | 0 | 0 | 0 | 0 |
| Hyaloscypha | 0 | 0 | 0 | 0 | 0 | 0 | 0 | 0 | 0 | 0 | 0 | 9392 | 0 | 0 | 0 |
| Hymenogaster | 0 | 0 | 0 | 6776 | 0 | 0 | 0 | 0 | 0 | 0 | 0 | 0 | 0 | 0 | 0 |
| Hyphodontia | 0 | 0 | 0 | 0 | 0 | 1780 | 0 | 0 | 0 | 0 | 0 | 0 | 0 | 0 | 0 |
| Idriella | 0 | 0 | 0 | 0 | 0 | 8206 | 0 | 0 | 0 | 0 | 0 | 0 | 0 | 0 | 0 |
| Ilyonectria | 0 | 0 | 2126 | 0 | 0 | 0 | 0 | 0 | 832 | 0 | 0 | 0 | 0 | 0 | 0 |
| Infundichalara | 0 | 1138 | 0 | 0 | 0 | 0 | 0 | 0 | 0 | 0 | 0 | 0 | 0 | 0 | 0 |
| Inocybe | 32287 | 0 | 0 | 1831 | 23744 | 1931 | 0 | 0 | 9707 | 0 | 0 | 0 | 0 | 0 | 0 |
| Laccaria | 2213 | 5171 | 0 | 4397 | 0 | 0 | 0 | 0 | 0 | 0 | 0 | 0 | 0 | 0 | 0 |
| Lachnum | 0 | 0 | 0 | 0 | 0 | 0 | 0 | 0 | 0 | 0 | 1117 | 0 | 0 | 0 | 0 |
| Lactarius | 20509 | 0 | 0 | 0 | 1202 | 0 | 0 | 0 | 15011 | 0 | 0 | 0 | 0 | 0 | 0 |
| Lasiosphaeris | 0 | 0 | 0 | 0 | 0 | 0 | 0 | 0 | 0 | 1422 | 0 | 0 | 0 | 0 | 0 |
| Leptodontidium | 0 | 0 | 4209 | 0 | 0 | 0 | 0 | 0 | 1645 | 0 | 0 | 1198 | 0 | 0 | 1845 |
| Lindgomyces | 0 | 0 | 0 | 0 | 0 | 0 | 0 | 0 | 0 | 0 | 0 | 0 | 0 | 3679 | 0 |
| Lophiostoma | 0 | 0 | 0 | 0 | 0 | 0 | 0 | 0 | 0 | 913 | 0 | 0 | 0 | 0 | 0 |
| Melanconium | 0 | 0 | 0 | 0 | 0 | 1924 | 0 | 0 | 0 | 0 | 0 | 0 | 0 | 0 | 0 |
| Melanogaster | 0 | 0 | 890 | 0 | 0 | 0 | 0 | 0 | 0 | 0 | 0 | 0 | 0 | 0 | 0 |
| Meliniomyces | 737 | 0 | 1067 | 0 | 0 | 0 | 0 | 0 | 1657 | 0 | 0 | 1086 | 0 | 0 | 0 |
| Mortierella | 2836 | 0 | 6283 | 0 | 0 | 2230 | 0 | 0 | 10437 | 0 | 1868 | 0 | 0 | 0 | 1891 |
| Murispora | 0 | 0 | 0 | 0 | 0 | 0 | 0 | 2609 | 0 | 0 | 0 | 0 | 0 | 0 | 0 |
| Mycena | 0 | 0 | 0 | 0 | 0 | 0 | 3790 | 0 | 0 | 0 | 1697 | 4055 | 0 | 0 | 0 |
| Naucoria | 0 | 0 | 0 | 1855 | 0 | 0 | 0 | 0 | 0 | 0 | 0 | 0 | 0 | 0 | 0 |
| Neoascochyta | 0 | 0 | 0 | 0 | 0 | 0 | 0 | 0 | 0 | 0 | 986 | 0 | 0 | 0 | 0 |
| Neopyrenochaeta | 0 | 0 | 0 | 0 | 764 | 3495 | 0 | 2441 | 0 | 2189 | 0 | 0 | 0 | 0 | 1902 |
| Ochrocladosporium | 0 | 0 | 0 | 0 | 0 | 0 | 0 | 1792 | 0 | 0 | 0 | 0 | 0 | 0 | 0 |
| Oidiodendron | 0 | 0 | 0 | 0 | 0 | 4673 | 0 | 4049 | 0 | 0 | 0 | 0 | 0 | 0 | 0 |
| Paraphaeosphaeria | 0 | 0 | 0 | 0 | 0 | 0 | 0 | 0 | 0 | 0 | 0 | 0 | 0 | 0 | 1143 |
| Parasola | 0 | 0 | 0 | 0 | 0 | 0 | 0 | 0 | 0 | 2975 | 0 | 0 | 0 | 0 | 0 |
| Paxillus | 0 | 0 | 0 | 0 | 0 | 0 | 0 | 0 | 2002 | 0 | 0 | 0 | 0 | 0 | 0 |
| Peziza | 0 | 0 | 0 | 0 | 0 | 5823 | 0 | 0 | 0 | 0 | 0 | 0 | 0 | 0 | 0 |
| Phialophora | 0 | 0 | 0 | 0 | 0 | 0 | 0 | 11687 | 0 | 0 | 0 | 0 | 0 | 0 | 0 |
| Pithomyces | 0 | 0 | 0 | 0 | 0 | 0 | 0 | 0 | 0 | 0 | 0 | 0 | 0 | 1969 | 0 |
| Pleurophragmium | 0 | 0 | 0 | 0 | 0 | 0 | 0 | 0 | 0 | 0 | 0 | 0 | 0 | 1441 | 0 |
| Preussia | 0 | 1396 | 0 | 0 | 0 | 994 | 0 | 821 | 0 | 0 | 0 | 0 | 0 | 0 | 0 |
| Psathyrella | 0 | 0 | 0 | 0 | 0 | 0 | 0 | 0 | 0 | 1621 | 0 | 0 | 0 | 0 | 0 |
| Pseudaleuria | 0 | 1518 | 0 | 0 | 0 | 0 | 0 | 0 | 0 | 0 | 0 | 0 | 0 | 0 | 0 |
| Pseudohydnum | 0 | 0 | 0 | 0 | 0 | 0 | 0 | 0 | 0 | 0 | 0 | 843 | 0 | 0 | 0 |
| Pseudotomentella | 0 | 0 | 0 | 0 | 0 | 0 | 0 | 0 | 0 | 0 | 1265 | 0 | 0 | 0 | 0 |
| Pyrenochaeta | 0 | 0 | 0 | 0 | 0 | 0 | 0 | 0 | 0 | 0 | 0 | 0 | 868 | 0 | 0 |
| Pyrenochaetopsis | 0 | 3114 | 0 | 1484 | 0 | 0 | 0 | 985 | 0 | 0 | 1691 | 0 | 1444 | 1233 | 2384 |
| Ramophialophora | 0 | 0 | 0 | 0 | 0 | 0 | 0 | 0 | 0 | 5553 | 0 | 0 | 0 | 8041 | 0 |
| Rosellinia | 0 | 0 | 0 | 0 | 0 | 0 | 0 | 0 | 0 | 0 | 0 | 0 | 0 | 0 | 927 |
| Russula | 12061 | 33756 | 1824 | 3513 | 3776 | 0 | 0 | 0 | 7224 | 10884 | 0 | 1925 | 0 | 0 | 7518 |
| Saitozyma | 0 | 1490 | 9704 | 0 | 3217 | 0 | 0 | 0 | 2217 | 1196 | 0 | 0 | 0 | 0 | 4684 |
| Schizothecium | 0 | 0 | 0 | 0 | 0 | 0 | 0 | 1685 | 0 | 0 | 0 | 0 | 0 | 0 | 0 |
| Scleroderma | 0 | 2107 | 7882 | 1112 | 895 | 0 | 0 | 0 | 0 | 0 | 0 | 0 | 0 | 0 | 0 |
| Scytalidium | 0 | 0 | 0 | 0 | 0 | 0 | 0 | 0 | 0 | 1239 | 0 | 0 | 0 | 0 | 0 |
| Serendipita | 0 | 0 | 0 | 0 | 0 | 876 | 0 | 0 | 0 | 0 | 0 | 0 | 0 | 0 | 0 |
| Setophoma | 0 | 0 | 0 | 0 | 0 | 0 | 0 | 0 | 0 | 0 | 0 | 0 | 0 | 1695 | 0 |
| Solicoccozyma | 0 | 0 | 15457 | 0 | 0 | 0 | 0 | 0 | 914 | 0 | 0 | 0 | 0 | 0 | 3102 |
| Sordaria | 0 | 0 | 0 | 0 | 0 | 4393 | 0 | 0 | 0 | 0 | 0 | 0 | 0 | 0 | 0 |
| Spondylocladiella | 0 | 0 | 0 | 0 | 0 | 0 | 0 | 0 | 0 | 2809 | 0 | 0 | 0 | 0 | 0 |
| Stypella | 0 | 0 | 0 | 0 | 0 | 0 | 0 | 0 | 0 | 0 | 0 | 6183 | 0 | 0 | 0 |
| Tausonia | 0 | 0 | 1643 | 0 | 2572 | 3126 | 0 | 0 | 950 | 1555 | 5514 | 6069 | 0 | 0 | 5371 |
| Tetracladium | 0 | 0 | 0 | 0 | 0 | 11326 | 0 | 1188 | 0 | 0 | 0 | 0 | 0 | 0 | 0 |
| Tetragoniomyces | 0 | 0 | 0 | 0 | 0 | 0 | 0 | 0 | 0 | 0 | 0 | 0 | 785 | 0 | 0 |
| Thelebolus | 0 | 0 | 0 | 0 | 0 | 2116 | 0 | 0 | 0 | 0 | 0 | 0 | 0 | 0 | 0 |
| Tolypocladium | 0 | 0 | 0 | 0 | 0 | 0 | 0 | 0 | 0 | 0 | 0 | 0 | 0 | 0 | 1171 |
| Tomentella | 0 | 3964 | 0 | 11199 | 1016 | 3820 | 0 | 0 | 4770 | 5481 | 14451 | 0 | 0 | 0 | 0 |
| Trechispora | 3668 | 0 | 0 | 0 | 0 | 0 | 0 | 0 | 907 | 0 | 0 | 0 | 0 | 0 | 0 |
| Trematosphaeria | 0 | 1253 | 0 | 2484 | 0 | 0 | 2285 | 0 | 0 | 0 | 1916 | 0 | 8652 | 0 | 1436 |
| Trichocladium | 0 | 0 | 0 | 0 | 0 | 0 | 0 | 0 | 0 | 0 | 0 | 0 | 691 | 0 | 0 |
| Tricholoma | 0 | 0 | 0 | 0 | 0 | 0 | 0 | 0 | 2586 | 0 | 0 | 0 | 0 | 0 | 0 |
| Trichophaea | 0 | 0 | 0 | 0 | 0 | 0 | 0 | 0 | 0 | 3613 | 0 | 0 | 0 | 0 | 0 |
| Trichosporon | 766 | 0 | 0 | 0 | 0 | 0 | 0 | 0 | 0 | 0 | 0 | 0 | 0 | 0 | 0 |
| Tricladium | 0 | 0 | 0 | 0 | 0 | 0 | 0 | 1646 | 0 | 0 | 0 | 0 | 0 | 0 | 0 |
| Tuber | 0 | 0 | 0 | 1012 | 0 | 0 | 0 | 0 | 0 | 863 | 0 | 0 | 0 | 0 | 0 |
| Tylospora | 0 | 0 | 0 | 0 | 0 | 0 | 0 | 0 | 0 | 0 | 11376 | 0 | 0 | 0 | 0 |
| Varicosporium | 0 | 0 | 0 | 0 | 0 | 0 | 0 | 0 | 0 | 0 | 0 | 1101 | 0 | 0 | 0 |
| Wilcoxina | 0 | 0 | 0 | 0 | 0 | 0 | 0 | 0 | 0 | 0 | 20620 | 0 | 0 | 0 | 0 |
| Xerocomus | 0 | 0 | 0 | 0 | 0 | 0 | 0 | 0 | 3085 | 0 | 0 | 0 | 0 | 0 | 0 |
| Unknown | 719 | 18493 | 44165 | 7502 | 47750 | 20976 | 6201 | 46662 | 11100 | 17476 | 14071 | 52745 | 4985 | 30246 | 16484 |
| **Total** | **116000** | **116000** | **116000** | **116000** | **116000** | **116000** | **116000** | **116000** | **116000** | **116000** | **116000** | **116000** | **116000** | **116000** | **116000** |

| **Phylum** | **LSU_1** | **LSU_2** | **LSU_3** | **LSU_4** | **LSU_5** | **LSU_6** | **LSU_7** | **LSU_8** | **LSU_9** | **LSU_10** | **LSU_11** | **LSU_12** | **LSU_13** | **LSU_14** | **LSU_15** |
| --- | --- | --- | --- | --- | --- | --- | --- | --- | --- | --- | --- | --- | --- | --- | --- |
| Ascomycota | 8179 | 13780 | 24174 | 20918 | 27593 | 38981 | 48885 | 56447 | 8767 | 12082 | 37001 | 32786 | 55388 | 54449 | 22514 |
| Basidiomycota | 49821 | 44220 | 33826 | 37082 | 30407 | 18608 | 8160 | 1232 | 48336 | 45918 | 20999 | 23781 | 2612 | 3139 | 35486 |
| Chytridiomycota | 0 | 0 | 0 | 0 | 0 | 0 | 0 | 321 | 0 | 0 | 0 | 0 | 0 | 0 | 0 |
| Unknown | 0 | 0 | 0 | 0 | 0 | 411 | 955 | 0 | 897 | 0 | 0 | 1433 | 0 | 412 | 0 |
| **Total** | **58000** | **58000** | **58000** | **58000** | **58000** | **58000** | **58000** | **58000** | **58000** | **58000** | **58000** | **58000** | **58000** | **58000** | **58000** |

| **Class** | **LSU_1** | **LSU_2** | **LSU_3** | **LSU_4** | **LSU_5** | **LSU_6** | **LSU_7** | **LSU_8** | **LSU_9** | **LSU_10** | **LSU_11** | **LSU_12** | **LSU_13** | **LSU_14** | **LSU_15** |
| --- | --- | --- | --- | --- | --- | --- | --- | --- | --- | --- | --- | --- | --- | --- | --- |
| Agaricomycetes | 49341 | 40967 | 7467 | 36489 | 23761 | 15544 | 5672 | 1232 | 45014 | 34218 | 19492 | 20529 | 1266 | 1390 | 27452 |
| Ascomycota_Incertae sedis | 0 | 603 | 0 | 0 | 0 | 3158 | 2826 | 2155 | 0 | 0 | 3879 | 0 | 3212 | 2153 | 562 |
| Cystobasidiomycetes | 0 | 0 | 0 | 0 | 0 | 0 | 0 | 0 | 0 | 0 | 0 | 0 | 746 | 0 | 0 |
| Dothideomycetes | 3256 | 4048 | 629 | 14488 | 11942 | 11688 | 28123 | 27671 | 1313 | 2074 | 3849 | 514 | 33402 | 41212 | 10508 |
| Eurotiomycetes | 1808 | 3843 | 15207 | 3647 | 12905 | 1100 | 8264 | 0 | 4122 | 1943 | 2500 | 1381 | 7719 | 469 | 7585 |
| Leotiomycetes | 756 | 1205 | 4814 | 538 | 2398 | 9778 | 6474 | 24237 | 881 | 1296 | 11218 | 27451 | 3948 | 0 | 932 |
| Microbotryomycetes | 0 | 0 | 0 | 0 | 0 | 0 | 0 | 0 | 0 | 0 | 0 | 0 | 0 | 784 | 0 |
| Pezizomycetes | 2359 | 646 | 0 | 1734 | 0 | 8268 | 0 | 662 | 384 | 4614 | 12953 | 0 | 0 | 6816 | 1375 |
| Saccharomycetes | 0 | 0 | 413 | 0 | 0 | 634 | 0 | 0 | 0 | 1611 | 0 | 0 | 0 | 615 | 0 |
| Sordariomycetes | 0 | 0 | 1421 | 0 | 348 | 407 | 0 | 0 | 2067 | 0 | 0 | 0 | 0 | 410 | 435 |
| Tremellomycetes | 480 | 3253 | 25934 | 593 | 6646 | 3064 | 2488 | 0 | 3322 | 10390 | 1507 | 2623 | 600 | 965 | 8034 |
| Unknown | 0 | 3435 | 2115 | 511 | 0 | 4359 | 4153 | 2043 | 897 | 1854 | 2602 | 5502 | 7107 | 3186 | 1117 |
| **Total** | **58000** | **58000** | **58000** | **58000** | **58000** | **58000** | **58000** | **58000** | **58000** | **58000** | **58000** | **58000** | **58000** | **58000** | **58000** |

| **Order** | **LSU_1** | **LSU_2** | **LSU_3** | **LSU_4** | **LSU_5** | **LSU_6** | **LSU_7** | **LSU_8** | **LSU_9** | **LSU_10** | **LSU_11** | **LSU_12** | **LSU_13** | **LSU_14** | **LSU_15** |
| --- | --- | --- | --- | --- | --- | --- | --- | --- | --- | --- | --- | --- | --- | --- | --- |
| Agaricales | 31264 | 15969 | 675 | 22572 | 13933 | 12465 | 2948 | 1232 | 18522 | 3830 | 1624 | 1756 | 447 | 902 | 622 |
| Ascomycota_Incertae sedis | 0 | 603 | 0 | 0 | 0 | 3158 | 2826 | 2155 | 0 | 0 | 3879 | 0 | 3212 | 2153 | 562 |
| Atheliales | 0 | 0 | 0 | 0 | 0 | 0 | 0 | 0 | 0 | 0 | 7856 | 0 | 0 | 0 | 0 |
| Auriculariales | 502 | 0 | 0 | 0 | 605 | 0 | 0 | 0 | 681 | 0 | 0 | 972 | 0 | 488 | 0 |
| Boletales | 1107 | 832 | 4933 | 2665 | 3770 | 0 | 0 | 0 | 3061 | 1241 | 0 | 0 | 0 | 0 | 8325 |
| Cantharellales | 0 | 0 | 0 | 0 | 0 | 0 | 0 | 0 | 0 | 797 | 0 | 0 | 396 | 0 | 0 |
| Capnodiales | 0 | 2684 | 629 | 13529 | 543 | 2900 | 26191 | 3180 | 0 | 0 | 2842 | 514 | 27346 | 16236 | 10508 |
| Chaetothyriales | 874 | 3843 | 14854 | 3647 | 12313 | 392 | 7365 | 0 | 4122 | 1533 | 2500 | 907 | 7719 | 469 | 6124 |
| Cystofilobasidiales | 0 | 0 | 498 | 0 | 807 | 970 | 0 | 0 | 0 | 400 | 1033 | 1961 | 0 | 0 | 1348 |
| Dothideales | 0 | 0 | 0 | 0 | 0 | 0 | 0 | 1242 | 0 | 0 | 0 | 0 | 0 | 0 | 0 |
| Erythrobasidiales | 0 | 0 | 0 | 0 | 0 | 0 | 0 | 0 | 0 | 0 | 0 | 0 | 746 | 0 | 0 |
| Eurotiales | 934 | 0 | 0 | 0 | 0 | 0 | 448 | 0 | 0 | 0 | 0 | 474 | 0 | 0 | 824 |
| Filobasidiales | 0 | 402 | 7856 | 0 | 684 | 0 | 523 | 0 | 597 | 0 | 0 | 0 | 0 | 965 | 2254 |
| Helotiales | 756 | 1205 | 4393 | 538 | 2398 | 8435 | 6474 | 24237 | 881 | 1296 | 11218 | 27451 | 3948 | 0 | 932 |
| Hymenochaetales | 0 | 0 | 0 | 0 | 0 | 466 | 0 | 0 | 0 | 0 | 0 | 0 | 0 | 0 | 0 |
| Hypocreales | 0 | 0 | 1002 | 0 | 0 | 0 | 0 | 0 | 1540 | 0 | 0 | 0 | 0 | 0 | 0 |
| Leotiales | 0 | 0 | 0 | 0 | 0 | 412 | 0 | 0 | 0 | 0 | 0 | 0 | 0 | 0 | 0 |
| Minutisphaerales | 0 | 0 | 0 | 0 | 0 | 855 | 0 | 0 | 0 | 0 | 0 | 0 | 0 | 0 | 0 |
| Mytilinidiales | 3256 | 0 | 0 | 486 | 2872 | 0 | 0 | 0 | 1313 | 471 | 0 | 0 | 0 | 0 | 0 |
| Onygenales | 0 | 0 | 0 | 0 | 592 | 708 | 451 | 0 | 0 | 0 | 0 | 0 | 0 | 0 | 637 |
| Pezizales | 2359 | 646 | 0 | 1734 | 0 | 8268 | 0 | 662 | 384 | 4614 | 12953 | 0 | 0 | 6816 | 1375 |
| Pleosporales | 0 | 1364 | 0 | 473 | 8527 | 7933 | 1932 | 23249 | 0 | 456 | 1007 | 0 | 5662 | 24976 | 0 |
| Russulales | 16159 | 21798 | 653 | 2084 | 3276 | 0 | 0 | 0 | 16403 | 11182 | 0 | 3494 | 0 | 0 | 15340 |
| Saccharomycetales | 0 | 0 | 413 | 0 | 0 | 634 | 0 | 0 | 0 | 1611 | 0 | 0 | 0 | 615 | 0 |
| Sebacinales | 0 | 0 | 0 | 0 | 0 | 0 | 0 | 0 | 0 | 466 | 655 | 0 | 0 | 0 | 0 |
| Sordariales | 0 | 0 | 419 | 0 | 348 | 407 | 0 | 0 | 527 | 0 | 0 | 0 | 0 | 410 | 435 |
| Sporidiobolales | 0 | 0 | 0 | 0 | 0 | 0 | 0 | 0 | 0 | 0 | 0 | 0 | 0 | 784 | 0 |
| Thelebolales | 0 | 0 | 0 | 0 | 0 | 931 | 0 | 0 | 0 | 0 | 0 | 0 | 0 | 0 | 0 |
| Thelephorales | 0 | 1990 | 0 | 7476 | 872 | 2613 | 0 | 0 | 2184 | 3894 | 8095 | 0 | 0 | 0 | 0 |
| Trechisporales | 0 | 378 | 0 | 1110 | 335 | 0 | 2341 | 0 | 4163 | 603 | 0 | 5725 | 0 | 0 | 1412 |
| Tremellales | 480 | 2217 | 15816 | 593 | 2969 | 0 | 1965 | 0 | 1104 | 1957 | 474 | 0 | 600 | 0 | 2471 |
| Trichosporonales | 0 | 634 | 1764 | 0 | 2186 | 2094 | 0 | 0 | 1621 | 8033 | 0 | 662 | 0 | 0 | 1961 |
| Tubeufiales | 0 | 0 | 0 | 0 | 0 | 0 | 0 | 0 | 0 | 1147 | 0 | 0 | 0 | 0 | 0 |
| Verrucariales | 0 | 0 | 353 | 0 | 0 | 0 | 0 | 0 | 0 | 0 | 0 | 0 | 0 | 0 | 0 |
| Unknown | 309 | 3435 | 3742 | 1093 | 970 | 4359 | 4536 | 2043 | 897 | 14469 | 3864 | 14084 | 7924 | 3186 | 2870 |
| **Total** | **58000** | **58000** | **58000** | **58000** | **58000** | **58000** | **58000** | **58000** | **58000** | **58000** | **58000** | **58000** | **58000** | **58000** | **58000** |

| **Family** | **LSU_1** | **LSU_2** | **LSU_3** | **LSU_4** | **LSU_5** | **LSU_6** | **LSU_7** | **LSU_8** | **LSU_9** | **LSU_10** | **LSU_11** | **LSU_12** | **LSU_13** | **LSU_14** | **LSU_15** |
| --- | --- | --- | --- | --- | --- | --- | --- | --- | --- | --- | --- | --- | --- | --- | --- |
| Amanitaceae | 15408 | 0 | 0 | 0 | 0 | 0 | 0 | 0 | 3739 | 0 | 0 | 0 | 0 | 0 | 0 |
| Amniculicolaceae | 0 | 0 | 0 | 0 | 0 | 0 | 0 | 2399 | 0 | 0 | 0 | 0 | 0 | 0 | 0 |
| Ascobolaceae | 0 | 0 | 0 | 0 | 0 | 523 | 0 | 0 | 0 | 0 | 0 | 0 | 0 | 4695 | 534 |
| Ascocorticiaceae | 0 | 0 | 0 | 0 | 0 | 0 | 0 | 0 | 0 | 0 | 939 | 4073 | 0 | 0 | 0 |
| Aspergillaceae | 0 | 0 | 0 | 0 | 0 | 0 | 448 | 0 | 0 | 0 | 0 | 474 | 0 | 0 | 824 |
| Atheliaceae | 0 | 0 | 0 | 0 | 0 | 0 | 0 | 0 | 0 | 0 | 7856 | 0 | 0 | 0 | 0 |
| Aureobasidiaceae | 0 | 0 | 0 | 0 | 0 | 0 | 0 | 364 | 0 | 0 | 0 | 0 | 0 | 0 | 0 |
| Auriculariales_Incertae sedis | 0 | 0 | 0 | 0 | 0 | 0 | 0 | 0 | 0 | 0 | 0 | 0 | 0 | 488 | 0 |
| Boletaceae | 1107 | 0 | 3282 | 1187 | 517 | 0 | 0 | 0 | 1365 | 448 | 0 | 0 | 0 | 0 | 8325 |
| Bulgariaceae | 0 | 0 | 0 | 0 | 0 | 412 | 0 | 0 | 0 | 0 | 0 | 0 | 0 | 0 | 0 |
| Cantharellales_Incertae sedis | 0 | 0 | 0 | 0 | 0 | 0 | 0 | 0 | 0 | 797 | 0 | 0 | 0 | 0 | 0 |
| Ceratobasidiaceae | 0 | 0 | 0 | 0 | 0 | 0 | 0 | 0 | 0 | 0 | 0 | 0 | 396 | 0 | 0 |
| Chaetomiaceae | 0 | 0 | 419 | 0 | 348 | 0 | 0 | 0 | 527 | 0 | 0 | 0 | 0 | 0 | 435 |
| Cladosporiaceae | 0 | 2684 | 0 | 13529 | 0 | 2900 | 26191 | 3180 | 0 | 0 | 2842 | 514 | 27346 | 16236 | 10508 |
| Clavariaceae | 0 | 581 | 0 | 0 | 0 | 0 | 533 | 0 | 0 | 0 | 1018 | 0 | 0 | 0 | 0 |
| Clavicipitaceae | 0 | 0 | 518 | 0 | 0 | 0 | 0 | 0 | 448 | 0 | 0 | 0 | 0 | 0 | 0 |
| Cordycipitaceae | 0 | 0 | 0 | 0 | 0 | 0 | 0 | 0 | 608 | 0 | 0 | 0 | 0 | 0 | 0 |
| Cortinariaceae | 0 | 3500 | 0 | 11632 | 0 | 0 | 0 | 0 | 8430 | 0 | 0 | 0 | 0 | 0 | 0 |
| Cucurbitariaceae | 0 | 0 | 0 | 0 | 0 | 0 | 0 | 390 | 0 | 0 | 0 | 0 | 0 | 0 | 0 |
| Cystofilobasidiaceae | 0 | 0 | 498 | 0 | 0 | 0 | 0 | 0 | 0 | 0 | 0 | 0 | 0 | 0 | 0 |
| Debaryomycetaceae | 0 | 0 | 0 | 0 | 0 | 634 | 0 | 0 | 0 | 0 | 0 | 0 | 0 | 0 | 0 |
| Dermateaceae | 0 | 0 | 0 | 0 | 0 | 0 | 0 | 3436 | 0 | 0 | 0 | 0 | 0 | 0 | 0 |
| Dictyosporiaceae | 0 | 0 | 0 | 0 | 0 | 424 | 0 | 0 | 0 | 0 | 0 | 0 | 0 | 0 | 0 |
| Didymellaceae | 0 | 0 | 0 | 0 | 0 | 0 | 0 | 637 | 0 | 0 | 0 | 0 | 0 | 13235 | 0 |
| Discinaceae | 321 | 0 | 0 | 0 | 0 | 0 | 0 | 0 | 0 | 0 | 0 | 0 | 0 | 0 | 0 |
| Dothioraceae | 0 | 0 | 0 | 0 | 0 | 0 | 0 | 878 | 0 | 0 | 0 | 0 | 0 | 0 | 0 |
| Entolomataceae | 0 | 647 | 0 | 0 | 0 | 0 | 0 | 0 | 0 | 0 | 0 | 0 | 0 | 0 | 0 |
| Epibryaceae | 0 | 0 | 697 | 0 | 0 | 0 | 0 | 0 | 0 | 0 | 0 | 0 | 0 | 0 | 0 |
| Erythrobasidiales_Incertae sedis | 0 | 0 | 0 | 0 | 0 | 0 | 0 | 0 | 0 | 0 | 0 | 0 | 746 | 0 | 0 |
| Exidiaceae | 502 | 0 | 0 | 0 | 605 | 0 | 0 | 0 | 681 | 0 | 0 | 0 | 0 | 0 | 0 |
| Filobasidiaceae | 0 | 0 | 0 | 0 | 0 | 0 | 0 | 0 | 0 | 0 | 0 | 0 | 0 | 965 | 0 |
| Gloniaceae | 3256 | 0 | 0 | 486 | 2872 | 0 | 0 | 0 | 1313 | 471 | 0 | 0 | 0 | 0 | 0 |
| Helotiaceae | 0 | 0 | 0 | 0 | 0 | 0 | 0 | 746 | 0 | 375 | 499 | 841 | 0 | 0 | 0 |
| Helotiales_Incertae sedis | 363 | 809 | 2100 | 0 | 0 | 6332 | 378 | 1106 | 881 | 0 | 2689 | 0 | 438 | 0 | 932 |
| Herpotrichiellaceae | 874 | 3843 | 12683 | 3647 | 12313 | 392 | 6164 | 0 | 3623 | 1533 | 1728 | 357 | 6542 | 469 | 3549 |
| Hyaloriaceae | 0 | 0 | 0 | 0 | 0 | 0 | 0 | 0 | 0 | 0 | 0 | 972 | 0 | 0 | 0 |
| Hyaloscyphaceae | 393 | 0 | 442 | 538 | 0 | 0 | 3295 | 0 | 0 | 0 | 4505 | 11282 | 1649 | 0 | 0 |
| Hydnangiaceae | 855 | 3854 | 0 | 2394 | 0 | 0 | 0 | 0 | 0 | 0 | 0 | 0 | 0 | 0 | 0 |
| Hydnodontaceae | 0 | 378 | 0 | 1110 | 335 | 0 | 2341 | 0 | 4163 | 603 | 0 | 5725 | 0 | 0 | 1412 |
| Hygrophoraceae | 0 | 4801 | 0 | 0 | 0 | 0 | 0 | 0 | 0 | 0 | 0 | 0 | 0 | 0 | 0 |
| Hygrophoropsidaceae | 0 | 0 | 0 | 0 | 0 | 0 | 0 | 0 | 0 | 793 | 0 | 0 | 0 | 0 | 0 |
| Hymenogastraceae | 0 | 1206 | 0 | 6649 | 0 | 2664 | 0 | 0 | 0 | 0 | 0 | 0 | 0 | 0 | 0 |
| Incertae sedis | 0 | 603 | 0 | 0 | 0 | 2405 | 2826 | 2155 | 0 | 0 | 1587 | 0 | 2580 | 2153 | 562 |
| Inocybaceae | 15001 | 0 | 0 | 1897 | 13277 | 9412 | 0 | 0 | 3732 | 0 | 0 | 0 | 0 | 0 | 622 |
| Lasiosphaeriaceae | 0 | 0 | 0 | 0 | 0 | 407 | 0 | 0 | 0 | 0 | 0 | 0 | 0 | 0 | 0 |
| Leotiaceae | 0 | 0 | 0 | 0 | 0 | 0 | 0 | 17959 | 0 | 0 | 1500 | 993 | 0 | 0 | 0 |
| Leptosphaeriaceae | 0 | 0 | 0 | 0 | 0 | 0 | 0 | 0 | 0 | 0 | 0 | 0 | 0 | 1022 | 0 |
| Lipomycetaceae | 0 | 0 | 0 | 0 | 0 | 0 | 0 | 0 | 0 | 1148 | 0 | 0 | 0 | 0 | 0 |
| Marasmiaceae | 0 | 429 | 0 | 0 | 0 | 0 | 0 | 0 | 0 | 0 | 0 | 0 | 0 | 0 | 0 |
| Melanogastraceae | 0 | 0 | 404 | 0 | 0 | 0 | 0 | 0 | 0 | 0 | 0 | 0 | 0 | 0 | 0 |
| Melanommataceae | 0 | 0 | 0 | 0 | 0 | 0 | 0 | 0 | 0 | 0 | 0 | 0 | 378 | 0 | 0 |
| Minutisphaeraceae | 0 | 0 | 0 | 0 | 0 | 855 | 0 | 0 | 0 | 0 | 0 | 0 | 0 | 0 | 0 |
| Morosphaeriaceae | 0 | 0 | 0 | 0 | 0 | 1156 | 0 | 0 | 0 | 0 | 0 | 0 | 0 | 0 | 0 |
| Mrakiaceae | 0 | 0 | 0 | 0 | 807 | 970 | 0 | 0 | 0 | 400 | 1033 | 1961 | 0 | 0 | 1348 |
| Myxotrichaceae | 0 | 0 | 0 | 0 | 364 | 0 | 0 | 0 | 0 | 0 | 0 | 0 | 0 | 0 | 0 |
| Nectriaceae | 0 | 0 | 484 | 0 | 0 | 0 | 0 | 0 | 484 | 0 | 0 | 0 | 0 | 0 | 0 |
| Nigrogranaceae | 0 | 0 | 0 | 0 | 0 | 1713 | 0 | 0 | 0 | 0 | 0 | 0 | 0 | 0 | 0 |
| Onygenaceae | 0 | 0 | 0 | 0 | 592 | 708 | 451 | 0 | 0 | 0 | 0 | 0 | 0 | 0 | 637 |
| Paxillaceae | 0 | 0 | 0 | 0 | 0 | 0 | 0 | 0 | 1018 | 0 | 0 | 0 | 0 | 0 | 0 |
| Pezizaceae | 0 | 0 | 0 | 0 | 0 | 3369 | 0 | 0 | 0 | 0 | 782 | 0 | 0 | 0 | 0 |
| Phaeosphaeriaceae | 0 | 0 | 0 | 0 | 0 | 822 | 0 | 539 | 0 | 0 | 441 | 0 | 0 | 912 | 0 |
| Piskurozymaceae | 0 | 402 | 7856 | 0 | 684 | 0 | 523 | 0 | 597 | 0 | 0 | 0 | 0 | 0 | 2254 |
| Pleosporaceae | 0 | 0 | 0 | 0 | 0 | 0 | 1008 | 0 | 0 | 0 | 0 | 0 | 2803 | 6708 | 0 |
| Psathyrellaceae | 0 | 0 | 0 | 0 | 0 | 389 | 0 | 1232 | 0 | 3431 | 0 | 0 | 447 | 902 | 0 |
| Pseudeurotiaceae | 0 | 0 | 0 | 0 | 0 | 753 | 0 | 0 | 0 | 0 | 2292 | 0 | 632 | 0 | 0 |
| Pyrenochaetopsidaceae | 0 | 0 | 0 | 0 | 0 | 0 | 0 | 0 | 0 | 0 | 0 | 0 | 588 | 469 | 0 |
| Pyronemataceae | 2038 | 646 | 0 | 692 | 0 | 2706 | 0 | 662 | 384 | 4183 | 11728 | 0 | 0 | 0 | 0 |
| Russulaceae | 16159 | 21798 | 653 | 2084 | 3276 | 0 | 0 | 0 | 16403 | 11182 | 0 | 362 | 0 | 0 | 15340 |
| Russulales_Incertae sedis | 0 | 0 | 0 | 0 | 0 | 0 | 0 | 0 | 0 | 0 | 0 | 3132 | 0 | 0 | 0 |
| Saccharomycetales | 0 | 0 | 0 | 0 | 0 | 0 | 0 | 0 | 0 | 463 | 0 | 0 | 0 | 615 | 0 |
| Saccharomycodaceae | 0 | 0 | 413 | 0 | 0 | 0 | 0 | 0 | 0 | 0 | 0 | 0 | 0 | 0 | 0 |
| Schizoporaceae | 0 | 0 | 0 | 0 | 0 | 466 | 0 | 0 | 0 | 0 | 0 | 0 | 0 | 0 | 0 |
| Sclerodermataceae | 0 | 832 | 1247 | 1478 | 3253 | 0 | 0 | 0 | 678 | 0 | 0 | 0 | 0 | 0 | 0 |
| Sclerotiniaceae | 0 | 0 | 0 | 0 | 0 | 0 | 675 | 0 | 0 | 0 | 0 | 724 | 0 | 0 | 0 |
| Sebacinaceae | 0 | 0 | 0 | 0 | 0 | 0 | 0 | 0 | 0 | 0 | 655 | 0 | 0 | 0 | 0 |
| Sordariaceae | 0 | 0 | 0 | 0 | 0 | 0 | 0 | 0 | 0 | 0 | 0 | 0 | 0 | 410 | 0 |
| Sporidiobolaceae | 0 | 0 | 0 | 0 | 0 | 0 | 0 | 0 | 0 | 0 | 0 | 0 | 0 | 784 | 0 |
| Sporormiaceae | 0 | 709 | 0 | 0 | 0 | 2462 | 0 | 1987 | 0 | 0 | 0 | 0 | 0 | 0 | 0 |
| Strophariaceae | 0 | 0 | 0 | 0 | 0 | 0 | 0 | 0 | 0 | 399 | 0 | 0 | 0 | 0 | 0 |
| Thelebolaceae | 0 | 0 | 0 | 0 | 0 | 931 | 0 | 0 | 0 | 0 | 0 | 0 | 0 | 0 | 0 |
| Thelephoraceae | 0 | 1990 | 0 | 7476 | 872 | 2613 | 0 | 0 | 2184 | 3894 | 8095 | 0 | 0 | 0 | 0 |
| Thyridariaceae | 0 | 0 | 0 | 0 | 0 | 1356 | 0 | 0 | 0 | 0 | 0 | 0 | 0 | 0 | 0 |
| Trematosphaeriaceae | 0 | 0 | 0 | 473 | 0 | 0 | 924 | 0 | 0 | 0 | 566 | 0 | 1893 | 558 | 0 |
| Tricholomataceae | 0 | 951 | 675 | 0 | 656 | 0 | 2415 | 0 | 2621 | 0 | 606 | 1756 | 0 | 0 | 0 |
| Trichosporonaceae | 0 | 634 | 1764 | 0 | 2186 | 2094 | 0 | 0 | 1621 | 8033 | 0 | 662 | 0 | 0 | 1961 |
| Trimorphomycetaceae | 480 | 2217 | 15816 | 593 | 2969 | 0 | 1965 | 0 | 1104 | 1957 | 474 | 0 | 600 | 0 | 2471 |
| Tuberaceae | 0 | 0 | 0 | 1042 | 0 | 843 | 0 | 0 | 0 | 431 | 0 | 0 | 0 | 0 | 0 |
| Tubeufiaceae | 0 | 0 | 0 | 0 | 0 | 0 | 0 | 0 | 0 | 1147 | 0 | 0 | 0 | 0 | 0 |
| Verrucariaceae | 0 | 0 | 353 | 0 | 0 | 0 | 0 | 0 | 0 | 0 | 0 | 0 | 0 | 0 | 0 |
| Unknown | 1243 | 4486 | 7696 | 1093 | 12074 | 7289 | 7863 | 20330 | 1396 | 16312 | 6165 | 24172 | 10962 | 7379 | 6286 |
| **Total** | **58000** | **58000** | **58000** | **58000** | **58000** | **58000** | **58000** | **58000** | **58000** | **58000** | **58000** | **58000** | **58000** | **58000** | **58000** |

| **Genus** | **LSU_1** | **LSU_2** | **LSU_3** | **LSU_4** | **LSU_5** | **LSU_6** | **LSU_7** | **LSU_8** | **LSU_9** | **LSU_10** | **LSU_11** | **LSU_12** | **LSU_13** | **LSU_14** | **LSU_15** |
| --- | --- | --- | --- | --- | --- | --- | --- | --- | --- | --- | --- | --- | --- | --- | --- |
| Acephala | 0 | 0 | 0 | 0 | 0 | 0 | 0 | 0 | 0 | 0 | 2247 | 0 | 0 | 0 | 0 |
| Acrocalymma | 0 | 0 | 0 | 0 | 0 | 1156 | 0 | 0 | 0 | 0 | 0 | 0 | 0 | 0 | 0 |
| Agrocybe | 0 | 0 | 0 | 0 | 0 | 0 | 0 | 0 | 0 | 399 | 0 | 0 | 0 | 0 | 0 |
| Alatospora | 0 | 0 | 0 | 0 | 0 | 0 | 0 | 17959 | 0 | 0 | 1500 | 993 | 0 | 0 | 0 |
| Alternaria | 0 | 0 | 0 | 0 | 0 | 0 | 0 | 0 | 0 | 0 | 0 | 0 | 0 | 713 | 0 |
| Amanita | 15408 | 0 | 0 | 0 | 0 | 0 | 0 | 0 | 3739 | 0 | 0 | 0 | 0 | 0 | 0 |
| Amphinema | 0 | 0 | 0 | 0 | 0 | 0 | 0 | 0 | 0 | 0 | 7856 | 0 | 0 | 0 | 0 |
| Apiotrichum | 0 | 634 | 1764 | 0 | 2186 | 2094 | 0 | 0 | 1621 | 8033 | 0 | 662 | 0 | 0 | 1961 |
| Ascobolus | 0 | 0 | 0 | 0 | 0 | 523 | 0 | 0 | 0 | 0 | 0 | 0 | 0 | 4695 | 534 |
| Ascocorticium | 0 | 0 | 0 | 0 | 0 | 0 | 0 | 0 | 0 | 0 | 939 | 4073 | 0 | 0 | 0 |
| Aspergillus | 0 | 0 | 0 | 0 | 0 | 0 | 0 | 0 | 0 | 0 | 0 | 0 | 0 | 0 | 0 |
| Aureobasidium | 0 | 0 | 0 | 0 | 0 | 0 | 0 | 364 | 0 | 0 | 0 | 0 | 0 | 0 | 0 |
| Basidiodendron | 502 | 0 | 0 | 0 | 605 | 0 | 0 | 0 | 681 | 0 | 0 | 0 | 0 | 0 | 0 |
| Belonioscyphella | 0 | 0 | 0 | 0 | 0 | 0 | 0 | 746 | 0 | 0 | 0 | 0 | 0 | 0 | 0 |
| Bipolaris | 0 | 0 | 0 | 0 | 0 | 0 | 1008 | 0 | 0 | 0 | 0 | 0 | 2803 | 4931 | 0 |
| Boidinia | 0 | 0 | 0 | 0 | 0 | 0 | 0 | 0 | 0 | 0 | 0 | 3132 | 0 | 0 | 0 |
| Botrytis | 0 | 0 | 0 | 0 | 0 | 0 | 0 | 0 | 0 | 0 | 0 | 724 | 0 | 0 | 0 |
| Byssonectria | 519 | 0 | 0 | 0 | 0 | 0 | 0 | 0 | 0 | 0 | 0 | 0 | 0 | 0 | 0 |
| Calycina | 0 | 0 | 0 | 0 | 0 | 0 | 2892 | 0 | 0 | 0 | 553 | 713 | 0 | 0 | 0 |
| Candida | 0 | 0 | 0 | 0 | 0 | 0 | 0 | 0 | 0 | 463 | 0 | 0 | 0 | 615 | 0 |
| Cenococcum | 3256 | 0 | 0 | 486 | 2872 | 0 | 0 | 0 | 1313 | 471 | 0 | 0 | 0 | 0 | 0 |
| Ceratosebacina | 0 | 0 | 0 | 0 | 0 | 0 | 0 | 0 | 0 | 0 | 0 | 0 | 0 | 488 | 0 |
| Chaetomium | 0 | 0 | 419 | 0 | 348 | 0 | 0 | 0 | 527 | 0 | 0 | 0 | 0 | 0 | 435 |
| Cheilymenia | 0 | 0 | 0 | 0 | 0 | 0 | 0 | 0 | 0 | 0 | 649 | 0 | 0 | 0 | 0 |
| Chrysosporium | 0 | 0 | 0 | 0 | 592 | 708 | 451 | 0 | 0 | 0 | 0 | 0 | 0 | 0 | 637 |
| Cladophialophora | 340 | 402 | 1717 | 0 | 702 | 0 | 0 | 0 | 369 | 0 | 0 | 357 | 0 | 0 | 0 |
| Cladosporium | 0 | 2684 | 0 | 13529 | 0 | 2900 | 26191 | 3180 | 0 | 0 | 2842 | 514 | 27346 | 16236 | 10508 |
| Claussenomyces | 0 | 0 | 0 | 0 | 0 | 0 | 0 | 0 | 0 | 0 | 499 | 0 | 0 | 0 | 0 |
| Clavaria | 0 | 0 | 0 | 0 | 0 | 0 | 0 | 0 | 0 | 0 | 1018 | 0 | 0 | 0 | 0 |
| Clavulinopsis | 0 | 581 | 0 | 0 | 0 | 0 | 0 | 0 | 0 | 0 | 0 | 0 | 0 | 0 | 0 |
| Clitocybe | 0 | 0 | 675 | 0 | 656 | 0 | 0 | 0 | 1082 | 0 | 0 | 0 | 0 | 0 | 0 |
| Coprinellus | 0 | 0 | 0 | 0 | 0 | 389 | 0 | 1232 | 0 | 0 | 0 | 0 | 0 | 0 | 0 |
| Coprinopsis | 0 | 0 | 0 | 0 | 0 | 0 | 0 | 0 | 0 | 1773 | 0 | 0 | 0 | 365 | 0 |
| Cortinarius | 0 | 3500 | 0 | 11632 | 0 | 0 | 0 | 0 | 8430 | 0 | 0 | 0 | 0 | 0 | 0 |
| Cuspidatispora | 0 | 0 | 0 | 0 | 0 | 407 | 0 | 0 | 0 | 0 | 0 | 0 | 0 | 0 | 0 |
| Cystofilobasidium | 0 | 0 | 498 | 0 | 0 | 0 | 0 | 0 | 0 | 0 | 0 | 0 | 0 | 0 | 0 |
| Dactylaria | 0 | 0 | 0 | 0 | 0 | 6332 | 378 | 1106 | 0 | 0 | 0 | 0 | 438 | 0 | 0 |
| Debaryomyces | 0 | 0 | 0 | 0 | 0 | 634 | 0 | 0 | 0 | 0 | 0 | 0 | 0 | 0 | 0 |
| Didymella | 0 | 0 | 0 | 0 | 0 | 0 | 0 | 637 | 0 | 0 | 0 | 0 | 0 | 12383 | 0 |
| Dothiora | 0 | 0 | 0 | 0 | 0 | 0 | 0 | 878 | 0 | 0 | 0 | 0 | 0 | 0 | 0 |
| Entoloma | 0 | 647 | 0 | 0 | 0 | 0 | 0 | 0 | 0 | 0 | 0 | 0 | 0 | 0 | 0 |
| Epibryon | 0 | 0 | 697 | 0 | 0 | 0 | 0 | 0 | 0 | 0 | 0 | 0 | 0 | 0 | 0 |
| Epicoccum | 0 | 0 | 0 | 0 | 0 | 0 | 0 | 0 | 0 | 0 | 0 | 0 | 0 | 852 | 0 |
| Exophiala | 534 | 3441 | 10966 | 3647 | 11611 | 392 | 6164 | 0 | 3254 | 1533 | 1728 | 0 | 6542 | 469 | 3549 |
| Genabea | 0 | 0 | 0 | 0 | 0 | 2127 | 0 | 0 | 0 | 0 | 0 | 0 | 0 | 0 | 0 |
| Genea | 1519 | 0 | 0 | 692 | 0 | 0 | 0 | 0 | 0 | 0 | 0 | 0 | 0 | 0 | 0 |
| Hebeloma | 0 | 1206 | 0 | 5491 | 0 | 2664 | 0 | 0 | 0 | 0 | 0 | 0 | 0 | 0 | 0 |
| Holwaya | 0 | 0 | 0 | 0 | 0 | 412 | 0 | 0 | 0 | 0 | 0 | 0 | 0 | 0 | 0 |
| Humaria | 0 | 0 | 0 | 0 | 0 | 0 | 0 | 0 | 384 | 0 | 0 | 0 | 0 | 0 | 0 |
| Hyalodendriella | 0 | 413 | 0 | 0 | 0 | 0 | 0 | 0 | 0 | 0 | 0 | 0 | 0 | 0 | 0 |
| Hyaloscypha | 393 | 0 | 442 | 0 | 0 | 0 | 0 | 0 | 0 | 0 | 2936 | 10569 | 0 | 0 | 0 |
| Hydnotrya | 321 | 0 | 0 | 0 | 0 | 0 | 0 | 0 | 0 | 0 | 0 | 0 | 0 | 0 | 0 |
| Hygrocybe | 0 | 4801 | 0 | 0 | 0 | 0 | 0 | 0 | 0 | 0 | 0 | 0 | 0 | 0 | 0 |
| Hyphodontia | 0 | 0 | 0 | 0 | 0 | 466 | 0 | 0 | 0 | 0 | 0 | 0 | 0 | 0 | 0 |
| Ilyonectria | 0 | 0 | 484 | 0 | 0 | 0 | 0 | 0 | 484 | 0 | 0 | 0 | 0 | 0 | 0 |
| Inocybe | 15001 | 0 | 0 | 1897 | 13277 | 9412 | 0 | 0 | 3732 | 0 | 0 | 0 | 0 | 0 | 622 |
| Jalapriya | 0 | 0 | 0 | 0 | 0 | 424 | 0 | 0 | 0 | 0 | 0 | 0 | 0 | 0 | 0 |
| Laccaria | 855 | 3854 | 0 | 2394 | 0 | 0 | 0 | 0 | 0 | 0 | 0 | 0 | 0 | 0 | 0 |
| Lachnum | 0 | 0 | 0 | 538 | 0 | 0 | 403 | 0 | 0 | 0 | 1016 | 0 | 1649 | 0 | 0 |
| Lactarius | 7390 | 0 | 0 | 0 | 823 | 0 | 0 | 0 | 10217 | 0 | 0 | 0 | 0 | 0 | 0 |
| Leccinum | 0 | 0 | 0 | 0 | 0 | 0 | 0 | 0 | 592 | 0 | 0 | 0 | 0 | 0 | 0 |
| Leptobacillium | 0 | 0 | 0 | 0 | 0 | 0 | 0 | 0 | 608 | 0 | 0 | 0 | 0 | 0 | 0 |
| Leptodontidium | 363 | 396 | 2100 | 0 | 0 | 0 | 0 | 0 | 881 | 0 | 442 | 0 | 0 | 0 | 932 |
| Leptosphaeria | 0 | 0 | 0 | 0 | 0 | 0 | 0 | 0 | 0 | 0 | 0 | 0 | 0 | 1022 | 0 |
| Leucogyrophana | 0 | 0 | 0 | 0 | 0 | 0 | 0 | 0 | 0 | 793 | 0 | 0 | 0 | 0 | 0 |
| Lipomyces | 0 | 0 | 0 | 0 | 0 | 0 | 0 | 0 | 0 | 1148 | 0 | 0 | 0 | 0 | 0 |
| Liua | 0 | 0 | 0 | 0 | 0 | 394 | 0 | 0 | 0 | 0 | 0 | 0 | 0 | 0 | 0 |
| Marasmius | 0 | 429 | 0 | 0 | 0 | 0 | 0 | 0 | 0 | 0 | 0 | 0 | 0 | 0 | 0 |
| Melanogaster | 0 | 0 | 404 | 0 | 0 | 0 | 0 | 0 | 0 | 0 | 0 | 0 | 0 | 0 | 0 |
| Metapochonia | 0 | 0 | 518 | 0 | 0 | 0 | 0 | 0 | 448 | 0 | 0 | 0 | 0 | 0 | 0 |
| Minutisphaera | 0 | 0 | 0 | 0 | 0 | 855 | 0 | 0 | 0 | 0 | 0 | 0 | 0 | 0 | 0 |
| Mucronella | 0 | 0 | 0 | 0 | 0 | 0 | 533 | 0 | 0 | 0 | 0 | 0 | 0 | 0 | 0 |
| Murispora | 0 | 0 | 0 | 0 | 0 | 0 | 0 | 2399 | 0 | 0 | 0 | 0 | 0 | 0 | 0 |
| Mycena | 0 | 951 | 0 | 0 | 0 | 0 | 2415 | 0 | 0 | 0 | 606 | 1756 | 0 | 0 | 0 |
| Nadsonia | 0 | 0 | 413 | 0 | 0 | 0 | 0 | 0 | 0 | 0 | 0 | 0 | 0 | 0 | 0 |
| Naganishia | 0 | 0 | 0 | 0 | 0 | 0 | 0 | 0 | 0 | 0 | 0 | 0 | 0 | 965 | 0 |
| Naucoria | 0 | 0 | 0 | 1158 | 0 | 0 | 0 | 0 | 0 | 0 | 0 | 0 | 0 | 0 | 0 |
| Neoconiothyrium | 0 | 0 | 0 | 0 | 0 | 413 | 0 | 0 | 0 | 0 | 0 | 0 | 0 | 0 | 0 |
| Neocucurbitaria | 0 | 0 | 0 | 0 | 0 | 0 | 0 | 390 | 0 | 0 | 0 | 0 | 0 | 0 | 0 |
| Neurospora | 0 | 0 | 0 | 0 | 0 | 0 | 0 | 0 | 0 | 0 | 0 | 0 | 0 | 410 | 0 |
| Nigrograna | 0 | 0 | 0 | 0 | 0 | 1713 | 0 | 0 | 0 | 0 | 0 | 0 | 0 | 0 | 0 |
| Oidiodendron | 0 | 0 | 0 | 0 | 364 | 0 | 0 | 0 | 0 | 0 | 0 | 0 | 0 | 0 | 0 |
| Parasola | 0 | 0 | 0 | 0 | 0 | 0 | 0 | 0 | 0 | 1658 | 0 | 0 | 447 | 537 | 0 |
| Paxillus | 0 | 0 | 0 | 0 | 0 | 0 | 0 | 0 | 1018 | 0 | 0 | 0 | 0 | 0 | 0 |
| Penicillium | 0 | 0 | 0 | 0 | 0 | 0 | 448 | 0 | 0 | 0 | 0 | 474 | 0 | 0 | 824 |
| Peziza | 0 | 0 | 0 | 0 | 0 | 3369 | 0 | 0 | 0 | 0 | 0 | 0 | 0 | 0 | 0 |
| Phaeosphaeria | 0 | 0 | 0 | 0 | 0 | 822 | 0 | 539 | 0 | 0 | 441 | 0 | 0 | 0 | 0 |
| Phylloporus | 0 | 0 | 3282 | 0 | 0 | 0 | 0 | 0 | 0 | 0 | 0 | 0 | 0 | 0 | 0 |
| Pithomyces | 0 | 0 | 0 | 0 | 0 | 0 | 0 | 0 | 0 | 0 | 0 | 0 | 0 | 1064 | 0 |
| Pleotrichocladium | 0 | 0 | 0 | 0 | 0 | 0 | 0 | 0 | 0 | 0 | 0 | 0 | 378 | 0 | 0 |
| Preussia | 0 | 709 | 0 | 0 | 0 | 2462 | 0 | 1987 | 0 | 0 | 0 | 0 | 0 | 0 | 0 |
| Pseudeurotium | 0 | 0 | 0 | 0 | 0 | 753 | 0 | 0 | 0 | 0 | 2292 | 0 | 632 | 0 | 0 |
| Pseudohelicomyces | 0 | 0 | 0 | 0 | 0 | 0 | 0 | 0 | 0 | 1147 | 0 | 0 | 0 | 0 | 0 |
| Pyrenochaetopsis | 0 | 0 | 0 | 0 | 0 | 0 | 0 | 0 | 0 | 0 | 0 | 0 | 588 | 469 | 0 |
| Rhizoctonia | 0 | 0 | 0 | 0 | 0 | 0 | 0 | 0 | 0 | 0 | 0 | 0 | 396 | 0 | 0 |
| Rhodotorula | 0 | 0 | 0 | 0 | 0 | 0 | 0 | 0 | 0 | 0 | 0 | 0 | 0 | 784 | 0 |
| Roussoella | 0 | 0 | 0 | 0 | 0 | 549 | 0 | 0 | 0 | 0 | 0 | 0 | 0 | 0 | 0 |
| Russula | 8769 | 21798 | 653 | 2084 | 2453 | 0 | 0 | 0 | 6186 | 11182 | 0 | 362 | 0 | 0 | 15340 |
| Saitozyma | 480 | 2217 | 15816 | 593 | 2969 | 0 | 1965 | 0 | 1104 | 1957 | 474 | 0 | 600 | 0 | 2471 |
| Sakaguchia | 0 | 0 | 0 | 0 | 0 | 0 | 0 | 0 | 0 | 0 | 0 | 0 | 746 | 0 | 0 |
| Scleroderma | 0 | 832 | 1247 | 1478 | 3253 | 0 | 0 | 0 | 678 | 0 | 0 | 0 | 0 | 0 | 0 |
| Sclerotinia | 0 | 0 | 0 | 0 | 0 | 0 | 675 | 0 | 0 | 0 | 0 | 0 | 0 | 0 | 0 |
| Scytalidium | 0 | 0 | 0 | 0 | 0 | 0 | 0 | 0 | 0 | 375 | 0 | 0 | 0 | 0 | 0 |
| Sebacina | 0 | 0 | 0 | 0 | 0 | 0 | 0 | 0 | 0 | 0 | 655 | 0 | 0 | 0 | 0 |
| Setophoma | 0 | 0 | 0 | 0 | 0 | 0 | 0 | 0 | 0 | 0 | 0 | 0 | 0 | 912 | 0 |
| Sistotrema | 0 | 0 | 0 | 0 | 0 | 0 | 0 | 0 | 0 | 797 | 0 | 0 | 0 | 0 | 0 |
| Solicoccozyma | 0 | 402 | 7856 | 0 | 684 | 0 | 523 | 0 | 597 | 0 | 0 | 0 | 0 | 0 | 2254 |
| Spermospora | 0 | 603 | 0 | 0 | 0 | 664 | 2826 | 0 | 0 | 0 | 1587 | 0 | 2580 | 2153 | 562 |
| Staurothele | 0 | 0 | 353 | 0 | 0 | 0 | 0 | 0 | 0 | 0 | 0 | 0 | 0 | 0 | 0 |
| Stypella | 0 | 0 | 0 | 0 | 0 | 0 | 0 | 0 | 0 | 0 | 0 | 972 | 0 | 0 | 0 |
| Subulicystidium | 0 | 0 | 0 | 0 | 0 | 0 | 0 | 0 | 0 | 603 | 0 | 0 | 0 | 0 | 0 |
| Tausonia | 0 | 0 | 0 | 0 | 807 | 970 | 0 | 0 | 0 | 400 | 1033 | 1961 | 0 | 0 | 1348 |
| Thelebolus | 0 | 0 | 0 | 0 | 0 | 931 | 0 | 0 | 0 | 0 | 0 | 0 | 0 | 0 | 0 |
| Tomentella | 0 | 0 | 0 | 0 | 0 | 2613 | 0 | 0 | 0 | 2446 | 6791 | 0 | 0 | 0 | 0 |
| Trechispora | 0 | 0 | 0 | 0 | 335 | 0 | 0 | 0 | 3660 | 0 | 0 | 0 | 0 | 0 | 0 |
| Trematosphaeria | 0 | 0 | 0 | 473 | 0 | 0 | 924 | 0 | 0 | 0 | 566 | 0 | 1893 | 558 | 0 |
| Tricholoma | 0 | 0 | 0 | 0 | 0 | 0 | 0 | 0 | 1539 | 0 | 0 | 0 | 0 | 0 | 0 |
| Trichophaea | 0 | 0 | 0 | 0 | 0 | 0 | 0 | 0 | 0 | 4183 | 0 | 0 | 0 | 0 | 0 |
| Tuber | 0 | 0 | 0 | 1042 | 0 | 843 | 0 | 0 | 0 | 431 | 0 | 0 | 0 | 0 | 0 |
| Vargamyces | 0 | 0 | 0 | 0 | 0 | 1741 | 0 | 2155 | 0 | 0 | 0 | 0 | 0 | 0 | 0 |
| Varicosporium | 0 | 0 | 0 | 0 | 0 | 0 | 0 | 0 | 0 | 0 | 0 | 841 | 0 | 0 | 0 |
| Wilcoxina | 0 | 0 | 0 | 0 | 0 | 0 | 0 | 0 | 0 | 0 | 11079 | 0 | 0 | 0 | 0 |
| Xerocomellus | 1107 | 0 | 0 | 1187 | 517 | 0 | 0 | 0 | 0 | 448 | 0 | 0 | 0 | 0 | 8325 |
| Xerocomus | 0 | 0 | 0 | 0 | 0 | 0 | 0 | 0 | 773 | 0 | 0 | 0 | 0 | 0 | 0 |
| Unknown | 1243 | 7500 | 7696 | 9679 | 12946 | 7868 | 10204 | 24428 | 4083 | 17760 | 8251 | 29897 | 10962 | 7379 | 7698 |
| **Total** | **58000** | **58000** | **58000** | **58000** | **58000** | **58000** | **58000** | **58000** | **58000** | **58000** | **58000** | **58000** | **58000** | **58000** | **58000** |

| **Phylum** | **All_1** | **All_2** | **All_3** | **All_4** | **All_5** | **All_6** | **All_7** | **All_8** | **All_9** | **All_10** | **All_11** | **All_12** | **All_13** | **All_14** | **All_15** |
| --- | --- | --- | --- | --- | --- | --- | --- | --- | --- | --- | --- | --- | --- | --- | --- |
| Ascomycota | 24207 | 69282 | 70398 | 104889 | 105217 | 165835 | 203624 | 248092 | 31447 | 72326 | 134073 | 132046 | 231546 | 222424 | 139636 |
| Basidiomycota | 231275 | 185185 | 179288 | 150067 | 149384 | 76399 | 22442 | 7964 | 201857 | 182394 | 116709 | 116897 | 7085 | 5747 | 104333 |
| Chytridiomycota | 0 | 0 | 0 | 0 | 0 | 0 | 0 | 321 | 0 | 0 | 0 | 0 | 0 | 0 | 0 |
| Mucoromycota | 4518 | 3894 | 7513 | 5044 | 2511 | 12922 | 10095 | 0 | 21129 | 3563 | 3859 | 2301 | 10091 | 2872 | 12062 |
| Unknown | 0 | 1639 | 2801 | 0 | 2888 | 4844 | 23839 | 3623 | 5567 | 1717 | 5359 | 8756 | 11278 | 28957 | 3969 |
| **Total** | **260000** | **260000** | **260000** | **260000** | **260000** | **260000** | **260000** | **260000** | **260000** | **260000** | **260000** | **260000** | **260000** | **260000** | **260000** |

| **Class** | **All_1** | **All_2** | **All_3** | **All_4** | **All_5** | **All_6** | **All_7** | **All_8** | **All_9** | **All_10** | **All_11** | **All_12** | **All_13** | **All_14** | **All_15** |
| --- | --- | --- | --- | --- | --- | --- | --- | --- | --- | --- | --- | --- | --- | --- | --- |
| Agaricomycetes | 230029 | 173310 | 39514 | 148869 | 119306 | 62667 | 16008 | 7964 | 183478 | 122096 | 108475 | 101096 | 3811 | 1996 | 56808 |
| Archaeorhizomycetes | 0 | 0 | 0 | 0 | 0 | 0 | 0 | 0 | 894 | 0 | 18832 | 0 | 0 | 0 | 0 |
| Ascomycota_Incertae sedis | 0 | 603 | 0 | 0 | 0 | 6677 | 2826 | 2155 | 0 | 2809 | 4435 | 0 | 3212 | 2153 | 562 |
| Cystobasidiomycetes | 0 | 0 | 0 | 0 | 0 | 0 | 0 | 0 | 0 | 0 | 0 | 0 | 746 | 0 | 0 |
| Dothideomycetes | 16227 | 34504 | 10377 | 90174 | 49411 | 50930 | 146753 | 102662 | 10786 | 22942 | 27910 | 5488 | 189496 | 164066 | 92208 |
| Eurotiomycetes | 4128 | 5272 | 20743 | 4968 | 18007 | 1100 | 14115 | 11687 | 8608 | 1943 | 2500 | 1381 | 14615 | 469 | 27404 |
| Leotiomycetes | 1493 | 16226 | 20832 | 1847 | 4950 | 42261 | 19440 | 92274 | 5666 | 8021 | 33915 | 121737 | 6796 | 1286 | 3431 |
| Microbotryomycetes | 0 | 0 | 0 | 0 | 0 | 0 | 0 | 0 | 0 | 0 | 0 | 0 | 0 | 784 | 0 |
| Mortierellomycetes | 4518 | 3894 | 7513 | 5044 | 2511 | 12922 | 10095 | 0 | 21129 | 3563 | 3859 | 2301 | 10091 | 2872 | 12062 |
| Pezizomycetes | 2359 | 2164 | 0 | 3917 | 0 | 22429 | 0 | 662 | 384 | 11414 | 39421 | 0 | 0 | 7593 | 1375 |
| Saccharomycetes | 0 | 0 | 413 | 0 | 0 | 1829 | 0 | 0 | 0 | 1611 | 0 | 0 | 0 | 615 | 0 |
| Sordariomycetes | 0 | 4696 | 9219 | 3472 | 3948 | 23704 | 16623 | 1685 | 5109 | 21798 | 2861 | 0 | 9534 | 43780 | 11300 |
| Tremellomycetes | 1246 | 11875 | 136834 | 1198 | 29286 | 13732 | 5215 | 0 | 18379 | 58988 | 8234 | 15172 | 2528 | 1983 | 42005 |
| Unknown | 0 | 7456 | 14555 | 511 | 32581 | 21749 | 28925 | 40911 | 5567 | 4815 | 9558 | 12825 | 19171 | 32403 | 12845 |
| **Total** | **260000** | **260000** | **260000** | **260000** | **260000** | **260000** | **260000** | **260000** | **260000** | **260000** | **260000** | **260000** | **260000** | **260000** | **260000** |

| **Order** | **All_1** | **All_2** | **All_3** | **All_4** | **All_5** | **All_6** | **All_7** | **All_8** | **All_9** | **All_10** | **All_11** | **All_12** | **All_13** | **All_14** | **All_15** |
| --- | --- | --- | --- | --- | --- | --- | --- | --- | --- | --- | --- | --- | --- | --- | --- |
| Agaricales | 122727 | 51990 | 2071 | 86359 | 63822 | 30804 | 8322 | 7964 | 83559 | 21899 | 3321 | 5811 | 447 | 902 | 622 |
| Archaeorhizomycetales | 0 | 0 | 0 | 0 | 0 | 0 | 0 | 0 | 0 | 0 | 18832 | 0 | 0 | 0 | 0 |
| Ascomycota_Incertae sedis | 0 | 603 | 0 | 0 | 0 | 6677 | 2826 | 2155 | 0 | 2809 | 4435 | 0 | 3212 | 2153 | 562 |
| Atheliales | 0 | 0 | 0 | 0 | 0 | 0 | 0 | 0 | 0 | 0 | 41966 | 0 | 0 | 0 | 0 |
| Auriculariales | 502 | 845 | 5289 | 11401 | 2453 | 0 | 0 | 0 | 2195 | 876 | 2622 | 10506 | 840 | 488 | 0 |
| Boletales | 1664 | 2939 | 18766 | 3777 | 5621 | 0 | 0 | 0 | 14474 | 1241 | 0 | 0 | 0 | 0 | 8325 |
| Cantharellales | 719 | 0 | 0 | 0 | 3701 | 0 | 0 | 0 | 1016 | 1881 | 7644 | 0 | 1360 | 0 | 0 |
| Capnodiales | 0 | 20202 | 4606 | 79749 | 1219 | 16883 | 139036 | 18063 | 1227 | 2425 | 14085 | 4470 | 162512 | 57251 | 82333 |
| Chaetothyriales | 874 | 5272 | 20390 | 4968 | 17415 | 392 | 12346 | 11687 | 8608 | 1533 | 2500 | 907 | 14615 | 469 | 24245 |
| Coniochaetales | 0 | 0 | 0 | 0 | 0 | 0 | 0 | 0 | 0 | 3076 | 0 | 0 | 1662 | 0 | 0 |
| Cystofilobasidiales | 0 | 0 | 2141 | 0 | 3379 | 6239 | 0 | 0 | 1792 | 3825 | 7760 | 11569 | 0 | 0 | 6719 |
| Diaporthales | 0 | 0 | 0 | 0 | 0 | 1924 | 0 | 0 | 0 | 0 | 0 | 0 | 0 | 0 | 0 |
| Dothideales | 0 | 0 | 0 | 0 | 0 | 0 | 0 | 7452 | 0 | 0 | 0 | 0 | 0 | 0 | 0 |
| Erythrobasidiales | 0 | 0 | 0 | 0 | 0 | 0 | 0 | 0 | 0 | 0 | 0 | 0 | 746 | 0 | 0 |
| Eurotiales | 3254 | 0 | 0 | 0 | 0 | 0 | 448 | 0 | 0 | 0 | 0 | 474 | 0 | 0 | 1662 |
| Filobasidiales | 0 | 402 | 23313 | 0 | 684 | 0 | 523 | 0 | 1511 | 0 | 0 | 0 | 0 | 1983 | 5356 |
| Geastrales | 0 | 2730 | 0 | 0 | 0 | 0 | 0 | 0 | 0 | 0 | 0 | 0 | 0 | 0 | 0 |
| Helotiales | 1493 | 11926 | 20411 | 1049 | 4950 | 36681 | 19440 | 92274 | 5666 | 8021 | 33034 | 121737 | 6796 | 1286 | 3431 |
| Hymenochaetales | 0 | 0 | 0 | 0 | 0 | 2246 | 0 | 0 | 0 | 0 | 0 | 802 | 0 | 0 | 0 |
| Hypocreales | 0 | 1171 | 3128 | 3472 | 0 | 3659 | 16623 | 0 | 2372 | 0 | 2861 | 0 | 7181 | 8005 | 8240 |
| Leotiales | 0 | 0 | 0 | 0 | 0 | 412 | 0 | 0 | 0 | 0 | 0 | 0 | 0 | 0 | 0 |
| Minutisphaerales | 0 | 0 | 0 | 0 | 0 | 855 | 0 | 0 | 0 | 0 | 0 | 0 | 0 | 0 | 0 |
| Mortierellales | 4518 | 3894 | 7513 | 5044 | 2511 | 12922 | 10095 | 0 | 21129 | 3563 | 3859 | 2301 | 10091 | 2872 | 12062 |
| Mytilinidiales | 16227 | 3281 | 0 | 5242 | 28013 | 0 | 0 | 0 | 9559 | 9517 | 4759 | 0 | 0 | 0 | 0 |
| Onygenales | 0 | 0 | 0 | 0 | 592 | 708 | 451 | 0 | 0 | 0 | 0 | 0 | 0 | 0 | 1497 |
| Pezizales | 2359 | 2164 | 0 | 3917 | 0 | 22429 | 0 | 662 | 384 | 11414 | 39421 | 0 | 0 | 7593 | 1375 |
| Pleosporales | 0 | 9655 | 4241 | 5183 | 20179 | 30391 | 7717 | 77147 | 0 | 8760 | 9066 | 0 | 26590 | 106815 | 9875 |
| Polyporales | 0 | 0 | 0 | 0 | 0 | 11053 | 0 | 0 | 0 | 0 | 0 | 0 | 0 | 0 | 0 |
| Russulales | 99905 | 104016 | 2477 | 9691 | 34836 | 0 | 0 | 0 | 61474 | 46210 | 0 | 37583 | 0 | 0 | 42813 |
| Saccharomycetales | 0 | 0 | 413 | 0 | 0 | 1829 | 0 | 0 | 0 | 1611 | 0 | 0 | 0 | 615 | 0 |
| Sebacinales | 0 | 0 | 0 | 0 | 0 | 1791 | 0 | 0 | 0 | 1835 | 2934 | 925 | 0 | 606 | 0 |
| Sordariales | 0 | 3525 | 6091 | 0 | 3948 | 8717 | 0 | 1685 | 1343 | 18722 | 0 | 0 | 691 | 33826 | 2133 |
| Sordariomycetes_Incertae sedis | 0 | 0 | 0 | 0 | 0 | 0 | 0 | 0 | 0 | 0 | 0 | 0 | 0 | 1441 | 0 |
| Sporidiobolales | 0 | 0 | 0 | 0 | 0 | 0 | 0 | 0 | 0 | 0 | 0 | 0 | 0 | 784 | 0 |
| Thelebolales | 0 | 0 | 0 | 0 | 0 | 5168 | 0 | 0 | 0 | 0 | 0 | 0 | 0 | 0 | 0 |
| Thelephorales | 0 | 9782 | 0 | 34333 | 4001 | 16773 | 0 | 0 | 10154 | 30808 | 43991 | 0 | 741 | 0 | 0 |
| Trechisporales | 4203 | 1008 | 0 | 2726 | 2524 | 0 | 5598 | 0 | 10606 | 2142 | 2115 | 12091 | 0 | 0 | 1412 |
| Tremellales | 480 | 5384 | 98797 | 1198 | 9477 | 0 | 4692 | 0 | 5389 | 5647 | 474 | 0 | 1743 | 0 | 14903 |
| Trichosporonales | 766 | 6089 | 12583 | 0 | 15746 | 7493 | 0 | 0 | 9687 | 49516 | 0 | 3603 | 785 | 0 | 15027 |
| Tubeufiales | 0 | 0 | 0 | 0 | 0 | 0 | 0 | 0 | 0 | 1147 | 0 | 0 | 0 | 0 | 0 |
| Venturiales | 0 | 0 | 1530 | 0 | 0 | 0 | 0 | 0 | 0 | 0 | 0 | 0 | 0 | 0 | 0 |
| Verrucariales | 0 | 0 | 353 | 0 | 0 | 0 | 0 | 0 | 0 | 0 | 0 | 0 | 0 | 0 | 0 |
| Xylariales | 0 | 0 | 0 | 0 | 0 | 9404 | 0 | 0 | 0 | 0 | 0 | 0 | 0 | 508 | 927 |
| Unknown | 309 | 13122 | 25887 | 1891 | 34929 | 24550 | 31883 | 40911 | 7855 | 21522 | 14321 | 47221 | 19988 | 32403 | 16481 |
| **Total** | **260000** | **260000** | **260000** | **260000** | **260000** | **260000** | **260000** | **260000** | **260000** | **260000** | **260000** | **260000** | **260000** | **260000** | **260000** |

| **Family** | **All_1** | **All_2** | **All_3** | **All_4** | **All_5** | **All_6** | **All_7** | **All_8** | **All_9** | **All_10** | **All_11** | **All_12** | **All_13** | **All_14** | **All_15** |
| --- | --- | --- | --- | --- | --- | --- | --- | --- | --- | --- | --- | --- | --- | --- | --- |
| Agaricaceae | 0 | 0 | 0 | 0 | 0 | 0 | 0 | 0 | 0 | 888 | 0 | 0 | 0 | 0 | 0 |
| Amanitaceae | 46942 | 0 | 0 | 0 | 0 | 0 | 0 | 0 | 15113 | 0 | 0 | 0 | 0 | 0 | 0 |
| Amniculicolaceae | 0 | 0 | 0 | 0 | 0 | 0 | 0 | 5008 | 0 | 0 | 0 | 0 | 0 | 0 | 0 |
| Antennulariellaceae | 0 | 0 | 0 | 0 | 0 | 0 | 3377 | 0 | 0 | 0 | 0 | 0 | 3207 | 0 | 791 |
| Archaeorhizomycetaceae | 0 | 0 | 0 | 0 | 0 | 0 | 0 | 0 | 0 | 0 | 17145 | 0 | 0 | 0 | 0 |
| Ascobolaceae | 0 | 0 | 0 | 0 | 0 | 1152 | 0 | 0 | 0 | 0 | 0 | 0 | 0 | 4695 | 534 |
| Ascocorticiaceae | 0 | 0 | 0 | 0 | 0 | 0 | 0 | 0 | 0 | 0 | 939 | 15556 | 0 | 0 | 0 |
| Ascodesmidaceae | 0 | 0 | 0 | 0 | 0 | 932 | 0 | 0 | 0 | 0 | 0 | 0 | 0 | 0 | 0 |
| Aspergillaceae | 0 | 0 | 0 | 0 | 0 | 0 | 448 | 0 | 0 | 0 | 0 | 474 | 0 | 0 | 1662 |
| Atheliaceae | 0 | 0 | 0 | 0 | 0 | 0 | 0 | 0 | 0 | 0 | 41966 | 0 | 0 | 0 | 0 |
| Aureobasidiaceae | 0 | 0 | 0 | 0 | 0 | 0 | 0 | 3075 | 0 | 0 | 0 | 0 | 0 | 0 | 0 |
| Auriculariaceae | 0 | 0 | 0 | 0 | 0 | 0 | 0 | 0 | 0 | 0 | 0 | 0 | 840 | 0 | 0 |
| Auriculariales_Incertae sedis | 0 | 0 | 0 | 0 | 0 | 0 | 0 | 0 | 0 | 0 | 0 | 0 | 0 | 488 | 0 |
| Biatriosporaceae | 0 | 1130 | 1471 | 0 | 0 | 4044 | 0 | 0 | 0 | 0 | 0 | 0 | 0 | 0 | 0 |
| Bionectriaceae | 0 | 0 | 0 | 0 | 0 | 0 | 0 | 0 | 0 | 0 | 0 | 0 | 0 | 0 | 2523 |
| Boletaceae | 1664 | 0 | 6303 | 1187 | 517 | 0 | 0 | 0 | 5950 | 448 | 0 | 0 | 0 | 0 | 8325 |
| Bulgariaceae | 0 | 0 | 0 | 0 | 0 | 412 | 0 | 0 | 0 | 0 | 0 | 0 | 0 | 0 | 0 |
| Cantharellales_Incertae sedis | 0 | 0 | 0 | 0 | 0 | 0 | 0 | 0 | 0 | 1881 | 0 | 0 | 0 | 0 | 0 |
| Ceratobasidiaceae | 719 | 0 | 0 | 0 | 3701 | 0 | 0 | 0 | 1016 | 0 | 0 | 0 | 1360 | 0 | 0 |
| Chaetomiaceae | 0 | 1885 | 5304 | 0 | 3948 | 0 | 0 | 0 | 1343 | 1307 | 0 | 0 | 691 | 0 | 2133 |
| Cladosporiaceae | 0 | 20202 | 3159 | 79749 | 676 | 16883 | 135659 | 18063 | 1227 | 2425 | 14085 | 4470 | 159305 | 57251 | 81542 |
| Clavariaceae | 0 | 581 | 0 | 0 | 0 | 0 | 533 | 0 | 0 | 0 | 1018 | 0 | 0 | 0 | 0 |
| Clavicipitaceae | 0 | 0 | 518 | 0 | 0 | 0 | 0 | 0 | 448 | 0 | 0 | 0 | 0 | 0 | 0 |
| Clavulinaceae | 0 | 0 | 0 | 0 | 0 | 0 | 0 | 0 | 0 | 0 | 7644 | 0 | 0 | 0 | 0 |
| Coniochaetaceae | 0 | 0 | 0 | 0 | 0 | 0 | 0 | 0 | 0 | 3076 | 0 | 0 | 1662 | 0 | 0 |
| Coprinaceae | 0 | 0 | 0 | 0 | 0 | 0 | 0 | 781 | 0 | 0 | 0 | 0 | 0 | 0 | 0 |
| Cordycipitaceae | 0 | 0 | 0 | 0 | 0 | 0 | 0 | 0 | 608 | 0 | 0 | 0 | 0 | 0 | 0 |
| Cortinariaceae | 1060 | 26354 | 0 | 49175 | 0 | 0 | 0 | 0 | 38958 | 0 | 0 | 0 | 0 | 0 | 0 |
| Cucurbitariaceae | 0 | 0 | 0 | 0 | 0 | 0 | 0 | 390 | 0 | 0 | 0 | 0 | 0 | 0 | 0 |
| Cystofilobasidiaceae | 0 | 0 | 498 | 0 | 0 | 0 | 0 | 0 | 0 | 0 | 0 | 0 | 0 | 0 | 0 |
| Debaryomycetaceae | 0 | 0 | 0 | 0 | 0 | 1829 | 0 | 0 | 0 | 0 | 0 | 0 | 0 | 0 | 0 |
| Dermateaceae | 0 | 1232 | 0 | 0 | 0 | 0 | 0 | 14595 | 0 | 0 | 2726 | 0 | 0 | 0 | 0 |
| Dictyosporiaceae | 0 | 0 | 0 | 0 | 0 | 424 | 0 | 0 | 0 | 0 | 0 | 0 | 0 | 3621 | 0 |
| Didymellaceae | 0 | 0 | 1629 | 742 | 0 | 1734 | 0 | 6307 | 0 | 0 | 986 | 0 | 0 | 63706 | 875 |
| Didymosphaeriaceae | 0 | 0 | 0 | 0 | 0 | 0 | 0 | 0 | 0 | 0 | 0 | 0 | 0 | 0 | 1143 |
| Discinaceae | 321 | 0 | 0 | 0 | 0 | 0 | 0 | 0 | 0 | 0 | 0 | 0 | 0 | 0 | 0 |
| Dothioraceae | 0 | 0 | 0 | 0 | 0 | 0 | 0 | 4377 | 0 | 0 | 0 | 0 | 0 | 0 | 0 |
| Elaphomycetaceae | 2320 | 0 | 0 | 0 | 0 | 0 | 0 | 0 | 0 | 0 | 0 | 0 | 0 | 0 | 0 |
| Entolomataceae | 0 | 647 | 0 | 0 | 0 | 0 | 0 | 0 | 0 | 0 | 0 | 0 | 0 | 0 | 0 |
| Epibryaceae | 0 | 0 | 697 | 0 | 0 | 0 | 0 | 0 | 0 | 0 | 0 | 0 | 0 | 0 | 0 |
| Erythrobasidiales_Incertae sedis | 0 | 0 | 0 | 0 | 0 | 0 | 0 | 0 | 0 | 0 | 0 | 0 | 746 | 0 | 0 |
| Exidiaceae | 502 | 0 | 0 | 0 | 605 | 0 | 0 | 0 | 681 | 0 | 0 | 0 | 0 | 0 | 0 |
| Filobasidiaceae | 0 | 0 | 0 | 0 | 0 | 0 | 0 | 0 | 0 | 0 | 0 | 0 | 0 | 1983 | 0 |
| Ganodermataceae | 0 | 0 | 0 | 0 | 0 | 11053 | 0 | 0 | 0 | 0 | 0 | 0 | 0 | 0 | 0 |
| Geastraceae | 0 | 2730 | 0 | 0 | 0 | 0 | 0 | 0 | 0 | 0 | 0 | 0 | 0 | 0 | 0 |
| Gloniaceae | 16227 | 3281 | 0 | 5242 | 28013 | 0 | 0 | 0 | 9559 | 9517 | 4759 | 0 | 0 | 0 | 0 |
| Helotiaceae | 737 | 1138 | 1067 | 0 | 0 | 5399 | 0 | 4969 | 1657 | 5229 | 499 | 5090 | 0 | 0 | 0 |
| Helotiales_Incertae sedis | 363 | 809 | 6309 | 0 | 0 | 23631 | 7167 | 2830 | 2526 | 0 | 12525 | 1721 | 3286 | 1286 | 3431 |
| Herpotrichiellaceae | 874 | 5272 | 15593 | 4968 | 17415 | 392 | 10116 | 11687 | 7479 | 1533 | 1728 | 357 | 11890 | 469 | 12740 |
| Hyaloriaceae | 0 | 0 | 0 | 0 | 0 | 0 | 0 | 0 | 0 | 0 | 0 | 9677 | 0 | 0 | 0 |
| Hyaloscyphaceae | 393 | 0 | 442 | 538 | 0 | 0 | 7027 | 0 | 0 | 0 | 7935 | 46214 | 1649 | 0 | 0 |
| Hydnangiaceae | 3068 | 11780 | 0 | 8405 | 0 | 0 | 0 | 0 | 0 | 0 | 0 | 0 | 0 | 0 | 0 |
| Hydnodontaceae | 4203 | 1008 | 0 | 2726 | 826 | 0 | 5598 | 0 | 5729 | 2142 | 1112 | 12091 | 0 | 0 | 1412 |
| Hygrophoraceae | 0 | 9381 | 0 | 0 | 0 | 0 | 0 | 0 | 0 | 0 | 0 | 0 | 0 | 0 | 0 |
| Hygrophoropsidaceae | 0 | 0 | 0 | 0 | 0 | 0 | 0 | 0 | 0 | 793 | 0 | 0 | 0 | 0 | 0 |
| Hymenogastraceae | 0 | 1867 | 0 | 24306 | 0 | 7403 | 0 | 0 | 0 | 0 | 0 | 0 | 0 | 0 | 0 |
| Hyponectriaceae | 0 | 0 | 0 | 0 | 0 | 0 | 0 | 0 | 0 | 0 | 0 | 0 | 0 | 508 | 0 |
| Incertae sedis | 0 | 603 | 0 | 0 | 0 | 5059 | 2826 | 2155 | 0 | 2809 | 1587 | 0 | 2580 | 2153 | 562 |
| Inocybaceae | 71657 | 0 | 0 | 4473 | 63166 | 21996 | 0 | 0 | 20572 | 0 | 0 | 0 | 0 | 0 | 622 |
| Lasiosphaeriaceae | 0 | 1640 | 0 | 0 | 0 | 3251 | 0 | 1685 | 0 | 10795 | 0 | 0 | 0 | 0 | 0 |
| Leotiaceae | 0 | 0 | 0 | 511 | 0 | 0 | 0 | 63699 | 0 | 0 | 2619 | 2374 | 0 | 0 | 0 |
| Leptosphaeriaceae | 0 | 0 | 0 | 0 | 0 | 0 | 0 | 0 | 0 | 0 | 0 | 0 | 0 | 2863 | 1473 |
| Lindgomycetaceae | 0 | 0 | 0 | 0 | 0 | 0 | 0 | 0 | 0 | 0 | 972 | 0 | 0 | 5544 | 0 |
| Lipomycetaceae | 0 | 0 | 0 | 0 | 0 | 0 | 0 | 0 | 0 | 1148 | 0 | 0 | 0 | 0 | 0 |
| Lophiostomataceae | 0 | 0 | 0 | 0 | 0 | 0 | 0 | 0 | 0 | 913 | 0 | 0 | 0 | 0 | 0 |
| Marasmiaceae | 0 | 429 | 0 | 0 | 0 | 0 | 0 | 0 | 0 | 0 | 0 | 0 | 0 | 0 | 0 |
| Massarinaceae | 0 | 0 | 0 | 0 | 0 | 965 | 0 | 0 | 0 | 0 | 0 | 0 | 0 | 0 | 0 |
| Melanconidaceae | 0 | 0 | 0 | 0 | 0 | 1924 | 0 | 0 | 0 | 0 | 0 | 0 | 0 | 0 | 0 |
| Melanogastraceae | 0 | 0 | 1294 | 0 | 0 | 0 | 0 | 0 | 0 | 0 | 0 | 0 | 0 | 0 | 0 |
| Melanommataceae | 0 | 0 | 0 | 0 | 0 | 0 | 0 | 0 | 0 | 0 | 0 | 0 | 378 | 0 | 0 |
| Microdochiaceae | 0 | 0 | 0 | 0 | 0 | 9404 | 0 | 0 | 0 | 0 | 0 | 0 | 0 | 0 | 0 |
| Minutisphaeraceae | 0 | 0 | 0 | 0 | 0 | 855 | 0 | 0 | 0 | 0 | 0 | 0 | 0 | 0 | 0 |
| Morosphaeriaceae | 0 | 0 | 0 | 0 | 0 | 1156 | 0 | 0 | 0 | 0 | 0 | 0 | 0 | 0 | 0 |
| Mortierellaceae | 4518 | 3894 | 7513 | 5044 | 2511 | 12922 | 10095 | 0 | 21129 | 3563 | 3859 | 2301 | 10091 | 2872 | 12062 |
| Mrakiaceae | 0 | 0 | 1643 | 0 | 3379 | 6239 | 0 | 0 | 1792 | 3825 | 7760 | 11569 | 0 | 0 | 6719 |
| Myxotrichaceae | 0 | 0 | 0 | 0 | 364 | 4673 | 0 | 4049 | 0 | 0 | 0 | 0 | 0 | 0 | 0 |
| Nectriaceae | 0 | 1171 | 2610 | 3472 | 0 | 1506 | 16623 | 0 | 1316 | 0 | 2861 | 0 | 7181 | 8005 | 4546 |
| Nigrogranaceae | 0 | 0 | 0 | 0 | 0 | 1713 | 0 | 0 | 0 | 0 | 0 | 0 | 0 | 0 | 0 |
| Onygenaceae | 0 | 0 | 0 | 0 | 592 | 708 | 451 | 0 | 0 | 0 | 0 | 0 | 0 | 0 | 637 |
| Onygenales_Incertae sedis | 0 | 0 | 0 | 0 | 0 | 0 | 0 | 0 | 0 | 0 | 0 | 0 | 0 | 0 | 860 |
| Ophiocordycipitaceae | 0 | 0 | 0 | 0 | 0 | 0 | 0 | 0 | 0 | 0 | 0 | 0 | 0 | 0 | 1171 |
| Paxillaceae | 0 | 0 | 0 | 0 | 0 | 0 | 0 | 0 | 3988 | 0 | 0 | 0 | 0 | 0 | 0 |
| Pezizaceae | 0 | 0 | 0 | 0 | 0 | 9872 | 0 | 0 | 0 | 0 | 782 | 0 | 0 | 0 | 0 |
| Phaeosphaeriaceae | 0 | 0 | 0 | 0 | 0 | 822 | 0 | 539 | 0 | 0 | 441 | 0 | 0 | 4584 | 0 |
| Physalacriaceae | 0 | 0 | 502 | 0 | 0 | 0 | 0 | 0 | 0 | 0 | 0 | 0 | 0 | 0 | 0 |
| Piskurozymaceae | 0 | 402 | 23313 | 0 | 684 | 0 | 523 | 0 | 1511 | 0 | 0 | 0 | 0 | 0 | 5356 |
| Pleosporaceae | 0 | 0 | 0 | 0 | 0 | 0 | 4508 | 0 | 0 | 0 | 0 | 0 | 9682 | 17632 | 0 |
| Pleosporales_Incertae sedis | 0 | 0 | 0 | 0 | 0 | 0 | 0 | 2521 | 0 | 0 | 0 | 0 | 868 | 0 | 0 |
| Psathyrellaceae | 0 | 0 | 0 | 0 | 0 | 1405 | 0 | 7183 | 0 | 19015 | 0 | 0 | 447 | 902 | 0 |
| Pseudeurotiaceae | 0 | 0 | 0 | 0 | 0 | 2878 | 0 | 0 | 0 | 0 | 2848 | 0 | 632 | 0 | 0 |
| Pyrenochaetopsidaceae | 0 | 3114 | 0 | 1484 | 764 | 3495 | 0 | 4109 | 0 | 2189 | 2337 | 0 | 2716 | 2686 | 4948 |
| Pyronemataceae | 2038 | 2164 | 0 | 1218 | 0 | 8803 | 0 | 662 | 384 | 10120 | 38196 | 0 | 0 | 777 | 0 |
| Russulaceae | 99905 | 104016 | 2477 | 9691 | 34836 | 0 | 0 | 0 | 61474 | 46210 | 0 | 3903 | 0 | 0 | 42813 |
| Russulales_Incertae sedis | 0 | 0 | 0 | 0 | 0 | 0 | 0 | 0 | 0 | 0 | 0 | 32567 | 0 | 0 | 0 |
| Saccharomycetales | 0 | 0 | 0 | 0 | 0 | 0 | 0 | 0 | 0 | 463 | 0 | 0 | 0 | 615 | 0 |
| Saccharomycodaceae | 0 | 0 | 413 | 0 | 0 | 0 | 0 | 0 | 0 | 0 | 0 | 0 | 0 | 0 | 0 |
| Schizoporaceae | 0 | 0 | 0 | 0 | 0 | 2246 | 0 | 0 | 0 | 0 | 0 | 0 | 0 | 0 | 0 |
| Sclerodermataceae | 0 | 2939 | 11169 | 2590 | 5104 | 0 | 0 | 0 | 4536 | 0 | 0 | 0 | 0 | 0 | 0 |
| Sclerotiniaceae | 0 | 0 | 0 | 0 | 0 | 0 | 675 | 0 | 0 | 0 | 0 | 2876 | 0 | 0 | 0 |
| Sebacinaceae | 0 | 0 | 0 | 0 | 0 | 0 | 0 | 0 | 0 | 0 | 2934 | 0 | 0 | 0 | 0 |
| Serendipitaceae | 0 | 0 | 0 | 0 | 0 | 1791 | 0 | 0 | 0 | 1369 | 0 | 0 | 0 | 606 | 0 |
| Sordariaceae | 0 | 0 | 0 | 0 | 0 | 4393 | 0 | 0 | 0 | 0 | 0 | 0 | 0 | 21354 | 0 |
| Sordariales_Incertae sedis | 0 | 0 | 0 | 0 | 0 | 0 | 0 | 0 | 0 | 5553 | 0 | 0 | 0 | 8041 | 0 |
| Sordariomycetes_Incertae sedis | 0 | 0 | 0 | 0 | 0 | 0 | 0 | 0 | 0 | 0 | 0 | 0 | 0 | 1441 | 0 |
| Sporidiobolaceae | 0 | 0 | 0 | 0 | 0 | 0 | 0 | 0 | 0 | 0 | 0 | 0 | 0 | 784 | 0 |
| Sporormiaceae | 0 | 2105 | 0 | 0 | 0 | 5583 | 0 | 4796 | 0 | 0 | 0 | 0 | 0 | 2395 | 0 |
| Strophariaceae | 0 | 0 | 0 | 0 | 0 | 0 | 0 | 0 | 0 | 399 | 0 | 0 | 0 | 0 | 0 |
| Tetragoniomycetaceae | 0 | 0 | 0 | 0 | 0 | 0 | 0 | 0 | 0 | 0 | 0 | 0 | 785 | 0 | 0 |
| Thelebolaceae | 0 | 0 | 0 | 0 | 0 | 3908 | 0 | 0 | 0 | 0 | 0 | 0 | 0 | 0 | 0 |
| Thelephoraceae | 0 | 9782 | 0 | 34333 | 4001 | 16773 | 0 | 0 | 10154 | 30808 | 43991 | 0 | 741 | 0 | 0 |
| Thyridariaceae | 0 | 0 | 0 | 0 | 0 | 4984 | 0 | 0 | 0 | 0 | 0 | 0 | 0 | 0 | 0 |
| Trematosphaeriaceae | 0 | 1253 | 0 | 2957 | 0 | 0 | 3209 | 0 | 0 | 0 | 2482 | 0 | 10545 | 558 | 1436 |
| Tricholomataceae | 0 | 951 | 1569 | 0 | 656 | 0 | 6957 | 0 | 8916 | 0 | 2303 | 5811 | 0 | 0 | 0 |
| Trichosporonaceae | 766 | 6089 | 12583 | 0 | 15746 | 7493 | 0 | 0 | 9687 | 49516 | 0 | 3603 | 0 | 0 | 15027 |
| Trimorphomycetaceae | 480 | 5384 | 98797 | 1198 | 9477 | 0 | 4692 | 0 | 5389 | 5647 | 474 | 0 | 1743 | 0 | 14903 |
| Tuberaceae | 0 | 0 | 0 | 2699 | 0 | 843 | 0 | 0 | 0 | 1294 | 0 | 0 | 0 | 0 | 0 |
| Tubeufiaceae | 0 | 0 | 0 | 0 | 0 | 0 | 0 | 0 | 0 | 1147 | 0 | 0 | 0 | 0 | 0 |
| Venturiaceae | 0 | 0 | 1530 | 0 | 0 | 0 | 0 | 0 | 0 | 0 | 0 | 0 | 0 | 0 | 0 |
| Verrucariaceae | 0 | 0 | 353 | 0 | 0 | 0 | 0 | 0 | 0 | 0 | 0 | 0 | 0 | 0 | 0 |
| Vibrisseaceae | 0 | 0 | 0 | 0 | 0 | 0 | 0 | 0 | 0 | 0 | 1978 | 0 | 0 | 0 | 0 |
| Xylariaceae | 0 | 0 | 0 | 0 | 0 | 0 | 0 | 0 | 0 | 0 | 0 | 0 | 0 | 0 | 927 |
| Unknown | 1243 | 24767 | 51244 | 13292 | 63019 | 37052 | 39516 | 96520 | 16858 | 33978 | 26509 | 99346 | 26975 | 42181 | 28827 |
| **Total** | **260000** | **260000** | **260000** | **260000** | **260000** | **260000** | **260000** | **260000** | **260000** | **260000** | **260000** | **260000** | **260000** | **260000** | **260000** |

| **Genus** | **All_1** | **All_2** | **All_3** | **All_4** | **All_5** | **All_6** | **All_7** | **All_8** | **All_9** | **All_10** | **All_11** | **All_12** | **All_13** | **All_14** | **All_15** |
| --- | --- | --- | --- | --- | --- | --- | --- | --- | --- | --- | --- | --- | --- | --- | --- |
| Acephala | 0 | 0 | 0 | 0 | 0 | 0 | 0 | 0 | 0 | 0 | 3261 | 0 | 0 | 0 | 0 |
| Acrocalymma | 0 | 0 | 0 | 0 | 0 | 1156 | 0 | 0 | 0 | 0 | 0 | 0 | 0 | 0 | 0 |
| Agaricus | 0 | 0 | 0 | 0 | 0 | 0 | 0 | 0 | 0 | 888 | 0 | 0 | 0 | 0 | 0 |
| Agrocybe | 0 | 0 | 0 | 0 | 0 | 0 | 0 | 0 | 0 | 399 | 0 | 0 | 0 | 0 | 0 |
| Alatospora | 0 | 0 | 0 | 0 | 0 | 0 | 0 | 62027 | 0 | 0 | 2619 | 1849 | 0 | 0 | 0 |
| Alternaria | 0 | 0 | 0 | 0 | 0 | 0 | 3500 | 0 | 0 | 0 | 0 | 0 | 6879 | 6540 | 0 |
| Amanita | 46942 | 0 | 0 | 0 | 0 | 0 | 0 | 0 | 15113 | 0 | 0 | 0 | 0 | 0 | 0 |
| Amphinema | 0 | 0 | 0 | 0 | 0 | 0 | 0 | 0 | 0 | 0 | 24083 | 0 | 0 | 0 | 0 |
| Anguillospora | 0 | 0 | 0 | 0 | 0 | 5399 | 0 | 2577 | 0 | 0 | 0 | 0 | 0 | 0 | 0 |
| Antennariella | 0 | 0 | 0 | 0 | 0 | 0 | 3377 | 0 | 0 | 0 | 0 | 0 | 3207 | 0 | 791 |
| Apiotrichum | 0 | 6089 | 11634 | 0 | 15018 | 7493 | 0 | 0 | 8759 | 48255 | 0 | 2978 | 0 | 0 | 11968 |
| Apodus | 0 | 1640 | 0 | 0 | 0 | 1240 | 0 | 0 | 0 | 2537 | 0 | 0 | 0 | 0 | 0 |
| Archaeorhizomyces | 0 | 0 | 0 | 0 | 0 | 0 | 0 | 0 | 0 | 0 | 17145 | 0 | 0 | 0 | 0 |
| Armillaria | 0 | 0 | 502 | 0 | 0 | 0 | 0 | 0 | 0 | 0 | 0 | 0 | 0 | 0 | 0 |
| Ascobolus | 0 | 0 | 0 | 0 | 0 | 1152 | 0 | 0 | 0 | 0 | 0 | 0 | 0 | 4695 | 534 |
| Ascocorticium | 0 | 0 | 0 | 0 | 0 | 0 | 0 | 0 | 0 | 0 | 939 | 15556 | 0 | 0 | 0 |
| Aspergillus | 0 | 0 | 0 | 0 | 0 | 0 | 0 | 0 | 0 | 0 | 0 | 0 | 0 | 0 | 838 |
| Aureobasidium | 0 | 0 | 0 | 0 | 0 | 0 | 0 | 3075 | 0 | 0 | 0 | 0 | 0 | 0 | 0 |
| Auricularia | 0 | 0 | 0 | 0 | 0 | 0 | 0 | 0 | 0 | 0 | 0 | 0 | 840 | 0 | 0 |
| Basidiodendron | 502 | 0 | 0 | 0 | 605 | 0 | 0 | 0 | 681 | 0 | 0 | 0 | 0 | 0 | 0 |
| Belonioscyphella | 0 | 0 | 0 | 0 | 0 | 0 | 0 | 746 | 0 | 0 | 0 | 0 | 0 | 0 | 0 |
| Biatriospora | 0 | 1130 | 1471 | 0 | 0 | 4044 | 0 | 0 | 0 | 0 | 0 | 0 | 0 | 0 | 0 |
| Bipolaris | 0 | 0 | 0 | 0 | 0 | 0 | 1008 | 0 | 0 | 0 | 0 | 0 | 2803 | 6569 | 0 |
| Bloxamia | 0 | 0 | 0 | 0 | 0 | 0 | 0 | 0 | 0 | 2512 | 0 | 0 | 0 | 0 | 0 |
| Boidinia | 0 | 0 | 0 | 0 | 0 | 0 | 0 | 0 | 0 | 0 | 0 | 32567 | 0 | 0 | 0 |
| Botrytis | 0 | 0 | 0 | 0 | 0 | 0 | 0 | 0 | 0 | 0 | 0 | 2876 | 0 | 0 | 0 |
| Brevicellicium | 0 | 0 | 0 | 0 | 0 | 0 | 0 | 0 | 0 | 0 | 1112 | 0 | 0 | 0 | 0 |
| Byssonectria | 519 | 0 | 0 | 0 | 0 | 0 | 0 | 0 | 0 | 0 | 0 | 0 | 0 | 0 | 0 |
| Cadophora | 0 | 0 | 0 | 0 | 0 | 0 | 4128 | 0 | 0 | 0 | 8822 | 0 | 2848 | 1286 | 0 |
| Calycina | 0 | 0 | 0 | 0 | 0 | 0 | 6624 | 0 | 0 | 0 | 553 | 713 | 0 | 0 | 0 |
| Candida | 0 | 0 | 0 | 0 | 0 | 0 | 0 | 0 | 0 | 463 | 0 | 0 | 0 | 615 | 0 |
| Cenococcum | 16227 | 3281 | 0 | 5242 | 28013 | 0 | 0 | 0 | 9559 | 9517 | 4759 | 0 | 0 | 0 | 0 |
| Cephaliophora | 0 | 0 | 0 | 0 | 0 | 932 | 0 | 0 | 0 | 0 | 0 | 0 | 0 | 0 | 0 |
| Ceratobasidium | 0 | 0 | 0 | 0 | 0 | 0 | 0 | 0 | 0 | 0 | 0 | 0 | 964 | 0 | 0 |
| Ceratosebacina | 0 | 0 | 0 | 0 | 0 | 0 | 0 | 0 | 0 | 0 | 0 | 0 | 0 | 488 | 0 |
| Chaetomium | 0 | 0 | 3087 | 0 | 2491 | 0 | 0 | 0 | 527 | 0 | 0 | 0 | 0 | 0 | 1276 |
| Chalara | 0 | 0 | 0 | 0 | 0 | 0 | 2661 | 0 | 0 | 0 | 0 | 0 | 0 | 0 | 0 |
| Cheilymenia | 0 | 0 | 0 | 0 | 0 | 0 | 0 | 0 | 0 | 0 | 649 | 0 | 0 | 0 | 0 |
| Chrysosporium | 0 | 0 | 0 | 0 | 592 | 708 | 451 | 0 | 0 | 0 | 0 | 0 | 0 | 0 | 1497 |
| Cladophialophora | 340 | 402 | 1717 | 0 | 702 | 0 | 0 | 0 | 369 | 0 | 0 | 357 | 0 | 0 | 0 |
| Cladosporium | 0 | 20202 | 3159 | 79749 | 676 | 16883 | 135659 | 18063 | 1227 | 2425 | 14085 | 4470 | 159305 | 57251 | 81542 |
| Claussenomyces | 0 | 0 | 0 | 0 | 0 | 0 | 0 | 0 | 0 | 0 | 499 | 0 | 0 | 0 | 0 |
| Clavaria | 0 | 0 | 0 | 0 | 0 | 0 | 0 | 0 | 0 | 0 | 1018 | 0 | 0 | 0 | 0 |
| Clavulina | 0 | 0 | 0 | 0 | 0 | 0 | 0 | 0 | 0 | 0 | 7644 | 0 | 0 | 0 | 0 |
| Clavulinopsis | 0 | 581 | 0 | 0 | 0 | 0 | 0 | 0 | 0 | 0 | 0 | 0 | 0 | 0 | 0 |
| Clitocybe | 0 | 0 | 1569 | 0 | 656 | 0 | 0 | 0 | 2908 | 0 | 0 | 0 | 0 | 0 | 0 |
| Clohesyomyces | 0 | 0 | 0 | 0 | 0 | 0 | 0 | 0 | 0 | 0 | 972 | 0 | 0 | 1865 | 0 |
| Clonostachys | 0 | 0 | 0 | 0 | 0 | 0 | 0 | 0 | 0 | 0 | 0 | 0 | 0 | 0 | 2523 |
| Cochliobolus | 0 | 0 | 0 | 0 | 0 | 0 | 0 | 0 | 0 | 0 | 0 | 0 | 0 | 1007 | 0 |
| Coniochaeta | 0 | 0 | 0 | 0 | 0 | 0 | 0 | 0 | 0 | 3076 | 0 | 0 | 0 | 0 | 0 |
| Coprinellus | 0 | 0 | 0 | 0 | 0 | 1405 | 0 | 7183 | 0 | 0 | 0 | 0 | 0 | 0 | 0 |
| Coprinopsis | 0 | 0 | 0 | 0 | 0 | 0 | 0 | 0 | 0 | 7551 | 0 | 0 | 0 | 365 | 0 |
| Coprinus | 0 | 0 | 0 | 0 | 0 | 0 | 0 | 781 | 0 | 0 | 0 | 0 | 0 | 0 | 0 |
| Cortinarius | 1060 | 26354 | 0 | 49175 | 0 | 0 | 0 | 0 | 38958 | 0 | 0 | 0 | 0 | 0 | 0 |
| Cryptosporiopsis | 0 | 0 | 0 | 0 | 0 | 0 | 0 | 5018 | 0 | 0 | 869 | 0 | 0 | 0 | 0 |
| Cuspidatispora | 0 | 0 | 0 | 0 | 0 | 2011 | 0 | 0 | 0 | 0 | 0 | 0 | 0 | 0 | 0 |
| Cystofilobasidium | 0 | 0 | 498 | 0 | 0 | 0 | 0 | 0 | 0 | 0 | 0 | 0 | 0 | 0 | 0 |
| Dactylaria | 0 | 0 | 0 | 0 | 0 | 6332 | 378 | 1106 | 0 | 0 | 0 | 0 | 438 | 0 | 0 |
| Debaryomyces | 0 | 0 | 0 | 0 | 0 | 1829 | 0 | 0 | 0 | 0 | 0 | 0 | 0 | 0 | 0 |
| Dictyosporium | 0 | 0 | 0 | 0 | 0 | 0 | 0 | 0 | 0 | 0 | 0 | 0 | 0 | 3621 | 0 |
| Didymella | 0 | 0 | 0 | 0 | 0 | 0 | 0 | 637 | 0 | 0 | 0 | 0 | 0 | 37912 | 0 |
| Dothiora | 0 | 0 | 0 | 0 | 0 | 0 | 0 | 878 | 0 | 0 | 0 | 0 | 0 | 0 | 0 |
| Elaphomyces | 2320 | 0 | 0 | 0 | 0 | 0 | 0 | 0 | 0 | 0 | 0 | 0 | 0 | 0 | 0 |
| Entoloma | 0 | 647 | 0 | 0 | 0 | 0 | 0 | 0 | 0 | 0 | 0 | 0 | 0 | 0 | 0 |
| Epibryon | 0 | 0 | 697 | 0 | 0 | 0 | 0 | 0 | 0 | 0 | 0 | 0 | 0 | 0 | 0 |
| Epicoccum | 0 | 0 | 1629 | 742 | 0 | 1734 | 0 | 3551 | 0 | 0 | 0 | 0 | 0 | 25794 | 875 |
| Exophiala | 534 | 4870 | 13876 | 4968 | 16713 | 392 | 10116 | 0 | 7110 | 1533 | 1728 | 0 | 11890 | 469 | 12740 |
| Fusarium | 0 | 1171 | 0 | 3472 | 0 | 0 | 16623 | 0 | 0 | 0 | 2861 | 0 | 7181 | 7091 | 4546 |
| Ganoderma | 0 | 0 | 0 | 0 | 0 | 11053 | 0 | 0 | 0 | 0 | 0 | 0 | 0 | 0 | 0 |
| Geastrum | 0 | 2730 | 0 | 0 | 0 | 0 | 0 | 0 | 0 | 0 | 0 | 0 | 0 | 0 | 0 |
| Genabea | 0 | 0 | 0 | 0 | 0 | 8224 | 0 | 0 | 0 | 0 | 0 | 0 | 0 | 0 | 0 |
| Genea | 1519 | 0 | 0 | 1218 | 0 | 0 | 0 | 0 | 0 | 0 | 0 | 0 | 0 | 0 | 0 |
| Gibberella | 0 | 0 | 0 | 0 | 0 | 0 | 0 | 0 | 0 | 0 | 0 | 0 | 0 | 914 | 0 |
| Hebeloma | 0 | 1206 | 0 | 8934 | 0 | 6602 | 0 | 0 | 0 | 0 | 0 | 0 | 0 | 0 | 0 |
| Holwaya | 0 | 0 | 0 | 0 | 0 | 412 | 0 | 0 | 0 | 0 | 0 | 0 | 0 | 0 | 0 |
| Hormiactis | 0 | 0 | 0 | 0 | 0 | 2654 | 0 | 0 | 0 | 0 | 0 | 0 | 0 | 0 | 0 |
| Hormonema | 0 | 0 | 0 | 0 | 0 | 0 | 0 | 3499 | 0 | 0 | 0 | 0 | 0 | 0 | 0 |
| Humaria | 0 | 0 | 0 | 0 | 0 | 0 | 0 | 0 | 384 | 0 | 0 | 0 | 0 | 0 | 0 |
| Hyalodendriella | 0 | 413 | 0 | 0 | 0 | 0 | 0 | 0 | 0 | 0 | 0 | 0 | 0 | 0 | 0 |
| Hyaloscypha | 393 | 0 | 442 | 0 | 0 | 0 | 0 | 0 | 0 | 0 | 4328 | 33070 | 0 | 0 | 0 |
| Hydnotrya | 321 | 0 | 0 | 0 | 0 | 0 | 0 | 0 | 0 | 0 | 0 | 0 | 0 | 0 | 0 |
| Hygrocybe | 0 | 9381 | 0 | 0 | 0 | 0 | 0 | 0 | 0 | 0 | 0 | 0 | 0 | 0 | 0 |
| Hymenogaster | 0 | 661 | 0 | 11362 | 0 | 801 | 0 | 0 | 0 | 0 | 0 | 0 | 0 | 0 | 0 |
| Hyphodontia | 0 | 0 | 0 | 0 | 0 | 2246 | 0 | 0 | 0 | 0 | 0 | 0 | 0 | 0 | 0 |
| Idriella | 0 | 0 | 0 | 0 | 0 | 9404 | 0 | 0 | 0 | 0 | 0 | 0 | 0 | 0 | 0 |
| Ilyonectria | 0 | 0 | 2610 | 0 | 0 | 0 | 0 | 0 | 1316 | 0 | 0 | 0 | 0 | 0 | 0 |
| Infundichalara | 0 | 1138 | 0 | 0 | 0 | 0 | 0 | 0 | 0 | 0 | 0 | 0 | 0 | 0 | 0 |
| Inocybe | 71657 | 0 | 0 | 4473 | 63166 | 21996 | 0 | 0 | 20572 | 0 | 0 | 0 | 0 | 0 | 622 |
| Itersonilia | 0 | 0 | 0 | 0 | 0 | 0 | 0 | 0 | 842 | 0 | 0 | 0 | 0 | 0 | 0 |
| Jalapriya | 0 | 0 | 0 | 0 | 0 | 424 | 0 | 0 | 0 | 0 | 0 | 0 | 0 | 0 | 0 |
| Juxtiphoma | 0 | 0 | 0 | 0 | 0 | 0 | 0 | 2119 | 0 | 0 | 0 | 0 | 0 | 0 | 0 |
| Laccaria | 3068 | 11780 | 0 | 8405 | 0 | 0 | 0 | 0 | 0 | 0 | 0 | 0 | 0 | 0 | 0 |
| Lachnum | 0 | 0 | 0 | 538 | 0 | 0 | 403 | 0 | 0 | 0 | 3054 | 0 | 1649 | 0 | 0 |
| Lactarius | 65934 | 0 | 0 | 0 | 23301 | 0 | 0 | 0 | 39866 | 0 | 0 | 0 | 0 | 0 | 0 |
| Lasiosphaeris | 0 | 0 | 0 | 0 | 0 | 0 | 0 | 0 | 0 | 1422 | 0 | 0 | 0 | 0 | 0 |
| Leccinum | 0 | 0 | 0 | 0 | 0 | 0 | 0 | 0 | 592 | 0 | 0 | 0 | 0 | 0 | 0 |
| Leotia | 0 | 0 | 0 | 511 | 0 | 0 | 0 | 0 | 0 | 0 | 0 | 0 | 0 | 0 | 0 |
| Leptobacillium | 0 | 0 | 0 | 0 | 0 | 0 | 0 | 0 | 608 | 0 | 0 | 0 | 0 | 0 | 0 |
| Leptodontidium | 363 | 396 | 6309 | 0 | 0 | 0 | 0 | 0 | 2526 | 0 | 442 | 1198 | 0 | 0 | 3431 |
| Leptosphaeria | 0 | 0 | 0 | 0 | 0 | 0 | 0 | 0 | 0 | 0 | 0 | 0 | 0 | 2863 | 1473 |
| Leucogyrophana | 0 | 0 | 0 | 0 | 0 | 0 | 0 | 0 | 0 | 793 | 0 | 0 | 0 | 0 | 0 |
| Lindgomyces | 0 | 0 | 0 | 0 | 0 | 0 | 0 | 0 | 0 | 0 | 0 | 0 | 0 | 3679 | 0 |
| Lipomyces | 0 | 0 | 0 | 0 | 0 | 0 | 0 | 0 | 0 | 1148 | 0 | 0 | 0 | 0 | 0 |
| Liua | 0 | 0 | 0 | 0 | 0 | 394 | 0 | 0 | 0 | 0 | 0 | 0 | 0 | 0 | 0 |
| Lophiostoma | 0 | 0 | 0 | 0 | 0 | 0 | 0 | 0 | 0 | 913 | 0 | 0 | 0 | 0 | 0 |
| Marasmius | 0 | 429 | 0 | 0 | 0 | 0 | 0 | 0 | 0 | 0 | 0 | 0 | 0 | 0 | 0 |
| Massarina | 0 | 0 | 0 | 0 | 0 | 965 | 0 | 0 | 0 | 0 | 0 | 0 | 0 | 0 | 0 |
| Melanconium | 0 | 0 | 0 | 0 | 0 | 1924 | 0 | 0 | 0 | 0 | 0 | 0 | 0 | 0 | 0 |
| Melanogaster | 0 | 0 | 1294 | 0 | 0 | 0 | 0 | 0 | 0 | 0 | 0 | 0 | 0 | 0 | 0 |
| Meliniomyces | 737 | 0 | 1067 | 0 | 0 | 0 | 0 | 0 | 1657 | 0 | 0 | 1086 | 0 | 0 | 0 |
| Metapochonia | 0 | 0 | 518 | 0 | 0 | 0 | 0 | 0 | 448 | 0 | 0 | 0 | 0 | 0 | 0 |
| Microdochium | 0 | 0 | 0 | 0 | 0 | 0 | 0 | 0 | 0 | 0 | 0 | 0 | 0 | 508 | 0 |
| Minutisphaera | 0 | 0 | 0 | 0 | 0 | 855 | 0 | 0 | 0 | 0 | 0 | 0 | 0 | 0 | 0 |
| Mollisia | 0 | 0 | 0 | 0 | 0 | 0 | 0 | 0 | 0 | 0 | 539 | 0 | 0 | 0 | 0 |
| Mortierella | 4518 | 3894 | 7513 | 5044 | 2511 | 12922 | 10095 | 0 | 21129 | 3563 | 3859 | 2301 | 10091 | 2872 | 12062 |
| Mucronella | 0 | 0 | 0 | 0 | 0 | 0 | 533 | 0 | 0 | 0 | 0 | 0 | 0 | 0 | 0 |
| Murispora | 0 | 0 | 0 | 0 | 0 | 0 | 0 | 5008 | 0 | 0 | 0 | 0 | 0 | 0 | 0 |
| Mycena | 0 | 951 | 0 | 0 | 0 | 0 | 6957 | 0 | 0 | 0 | 2303 | 5811 | 0 | 0 | 0 |
| Nadsonia | 0 | 0 | 413 | 0 | 0 | 0 | 0 | 0 | 0 | 0 | 0 | 0 | 0 | 0 | 0 |
| Naganishia | 0 | 0 | 0 | 0 | 0 | 0 | 0 | 0 | 0 | 0 | 0 | 0 | 0 | 1983 | 0 |
| Naucoria | 0 | 0 | 0 | 4010 | 0 | 0 | 0 | 0 | 0 | 0 | 0 | 0 | 0 | 0 | 0 |
| Neoascochyta | 0 | 0 | 0 | 0 | 0 | 0 | 0 | 0 | 0 | 0 | 986 | 0 | 0 | 0 | 0 |
| Neobulgaria | 0 | 0 | 0 | 0 | 0 | 0 | 0 | 1672 | 0 | 0 | 0 | 0 | 0 | 0 | 0 |
| Neoconiothyrium | 0 | 0 | 0 | 0 | 0 | 413 | 0 | 0 | 0 | 0 | 0 | 0 | 0 | 0 | 0 |
| Neocucurbitaria | 0 | 0 | 0 | 0 | 0 | 0 | 0 | 390 | 0 | 0 | 0 | 0 | 0 | 0 | 0 |
| Neopyrenochaeta | 0 | 0 | 0 | 0 | 764 | 3495 | 0 | 2441 | 0 | 2189 | 0 | 0 | 0 | 0 | 1902 |
| Neurospora | 0 | 0 | 0 | 0 | 0 | 0 | 0 | 0 | 0 | 0 | 0 | 0 | 0 | 410 | 0 |
| Nigrograna | 0 | 0 | 0 | 0 | 0 | 1713 | 0 | 0 | 0 | 0 | 0 | 0 | 0 | 0 | 0 |
| Ochrocladosporium | 0 | 0 | 0 | 0 | 0 | 0 | 0 | 2521 | 0 | 0 | 0 | 0 | 0 | 0 | 0 |
| Oidiodendron | 0 | 0 | 0 | 0 | 364 | 4673 | 0 | 4049 | 0 | 0 | 0 | 0 | 0 | 0 | 0 |
| Paraphaeosphaeria | 0 | 0 | 0 | 0 | 0 | 0 | 0 | 0 | 0 | 0 | 0 | 0 | 0 | 0 | 1143 |
| Parasola | 0 | 0 | 0 | 0 | 0 | 0 | 0 | 0 | 0 | 8656 | 0 | 0 | 447 | 537 | 0 |
| Paxillus | 0 | 0 | 0 | 0 | 0 | 0 | 0 | 0 | 3988 | 0 | 0 | 0 | 0 | 0 | 0 |
| Penicillium | 0 | 0 | 0 | 0 | 0 | 0 | 448 | 0 | 0 | 0 | 0 | 474 | 0 | 0 | 824 |
| Peziza | 0 | 0 | 0 | 0 | 0 | 9872 | 0 | 0 | 0 | 0 | 0 | 0 | 0 | 0 | 0 |
| Pezoloma | 0 | 0 | 0 | 0 | 0 | 0 | 0 | 0 | 0 | 0 | 0 | 525 | 0 | 0 | 0 |
| Phaeosphaeria | 0 | 0 | 0 | 0 | 0 | 822 | 0 | 539 | 0 | 0 | 441 | 0 | 0 | 0 | 0 |
| Phialocephala | 0 | 0 | 0 | 0 | 0 | 0 | 0 | 0 | 0 | 0 | 1978 | 0 | 0 | 0 | 0 |
| Phialophora | 0 | 0 | 0 | 0 | 0 | 0 | 0 | 11687 | 0 | 0 | 0 | 0 | 0 | 0 | 0 |
| Phylloporus | 0 | 0 | 6303 | 0 | 0 | 0 | 0 | 0 | 0 | 0 | 0 | 0 | 0 | 0 | 0 |
| Pithomyces | 0 | 0 | 0 | 0 | 0 | 0 | 0 | 0 | 0 | 0 | 0 | 0 | 0 | 4523 | 0 |
| Pleotrichocladium | 0 | 0 | 0 | 0 | 0 | 0 | 0 | 0 | 0 | 0 | 0 | 0 | 378 | 0 | 0 |
| Pleurophragmium | 0 | 0 | 0 | 0 | 0 | 0 | 0 | 0 | 0 | 0 | 0 | 0 | 0 | 1441 | 0 |
| Preussia | 0 | 2105 | 0 | 0 | 0 | 5583 | 0 | 4796 | 0 | 0 | 0 | 0 | 0 | 2395 | 0 |
| Psathyrella | 0 | 0 | 0 | 0 | 0 | 0 | 0 | 0 | 0 | 2808 | 0 | 0 | 0 | 0 | 0 |
| Pseudaleuria | 0 | 1518 | 0 | 0 | 0 | 0 | 0 | 0 | 0 | 0 | 0 | 0 | 0 | 0 | 0 |
| Pseudeurotium | 0 | 0 | 0 | 0 | 0 | 1618 | 0 | 0 | 0 | 0 | 2848 | 0 | 632 | 0 | 0 |
| Pseudogymnoascus | 0 | 0 | 0 | 0 | 0 | 1260 | 0 | 0 | 0 | 0 | 0 | 0 | 0 | 0 | 0 |
| Pseudohelicomyces | 0 | 0 | 0 | 0 | 0 | 0 | 0 | 0 | 0 | 1147 | 0 | 0 | 0 | 0 | 0 |
| Pseudohydnum | 0 | 0 | 0 | 0 | 0 | 0 | 0 | 0 | 0 | 0 | 0 | 843 | 0 | 0 | 0 |
| Pseudotomentella | 0 | 0 | 0 | 0 | 0 | 0 | 0 | 0 | 0 | 0 | 1265 | 0 | 0 | 0 | 0 |
| Pyrenochaeta | 0 | 0 | 0 | 0 | 0 | 0 | 0 | 0 | 0 | 0 | 0 | 0 | 868 | 0 | 0 |
| Pyrenochaetopsis | 0 | 3114 | 0 | 1484 | 0 | 0 | 0 | 1668 | 0 | 0 | 2337 | 0 | 2716 | 2686 | 3046 |
| Ramophialophora | 0 | 0 | 0 | 0 | 0 | 0 | 0 | 0 | 0 | 5553 | 0 | 0 | 0 | 8041 | 0 |
| Rhizoctonia | 0 | 0 | 0 | 0 | 0 | 0 | 0 | 0 | 0 | 0 | 0 | 0 | 396 | 0 | 0 |
| Rhodotorula | 0 | 0 | 0 | 0 | 0 | 0 | 0 | 0 | 0 | 0 | 0 | 0 | 0 | 784 | 0 |
| Rosellinia | 0 | 0 | 0 | 0 | 0 | 0 | 0 | 0 | 0 | 0 | 0 | 0 | 0 | 0 | 927 |
| Roussoella | 0 | 0 | 0 | 0 | 0 | 549 | 0 | 0 | 0 | 0 | 0 | 0 | 0 | 0 | 0 |
| Russula | 33971 | 104016 | 2477 | 9691 | 11535 | 0 | 0 | 0 | 21608 | 46210 | 0 | 3903 | 0 | 0 | 42813 |
| Saitozyma | 480 | 5384 | 98797 | 1198 | 9477 | 0 | 4692 | 0 | 5389 | 5647 | 474 | 0 | 1743 | 0 | 14903 |
| Sakaguchia | 0 | 0 | 0 | 0 | 0 | 0 | 0 | 0 | 0 | 0 | 0 | 0 | 746 | 0 | 0 |
| Schizothecium | 0 | 0 | 0 | 0 | 0 | 0 | 0 | 1685 | 0 | 0 | 0 | 0 | 0 | 0 | 0 |
| Scleroderma | 0 | 2939 | 11169 | 2590 | 5104 | 0 | 0 | 0 | 4536 | 0 | 0 | 0 | 0 | 0 | 0 |
| Sclerotinia | 0 | 0 | 0 | 0 | 0 | 0 | 675 | 0 | 0 | 0 | 0 | 0 | 0 | 0 | 0 |
| Scytalidium | 0 | 0 | 0 | 0 | 0 | 0 | 0 | 0 | 0 | 2717 | 0 | 0 | 0 | 0 | 0 |
| Sebacina | 0 | 0 | 0 | 0 | 0 | 0 | 0 | 0 | 0 | 0 | 2934 | 0 | 0 | 0 | 0 |
| Serendipita | 0 | 0 | 0 | 0 | 0 | 1791 | 0 | 0 | 0 | 1369 | 0 | 0 | 0 | 606 | 0 |
| Setophoma | 0 | 0 | 0 | 0 | 0 | 0 | 0 | 0 | 0 | 0 | 0 | 0 | 0 | 3577 | 0 |
| Sistotrema | 0 | 0 | 0 | 0 | 0 | 0 | 0 | 0 | 0 | 1881 | 0 | 0 | 0 | 0 | 0 |
| Solicoccozyma | 0 | 402 | 23313 | 0 | 684 | 0 | 523 | 0 | 1511 | 0 | 0 | 0 | 0 | 0 | 5356 |
| Sordaria | 0 | 0 | 0 | 0 | 0 | 4393 | 0 | 0 | 0 | 0 | 0 | 0 | 0 | 0 | 0 |
| Spermospora | 0 | 603 | 0 | 0 | 0 | 664 | 2826 | 0 | 0 | 0 | 1587 | 0 | 2580 | 2153 | 562 |
| Spondylocladiella | 0 | 0 | 0 | 0 | 0 | 0 | 0 | 0 | 0 | 2809 | 0 | 0 | 0 | 0 | 0 |
| Staurothele | 0 | 0 | 353 | 0 | 0 | 0 | 0 | 0 | 0 | 0 | 0 | 0 | 0 | 0 | 0 |
| Stypella | 0 | 0 | 0 | 0 | 0 | 0 | 0 | 0 | 0 | 0 | 0 | 8834 | 0 | 0 | 0 |
| Subulicystidium | 0 | 0 | 0 | 0 | 0 | 0 | 0 | 0 | 0 | 2142 | 0 | 0 | 0 | 0 | 0 |
| Tausonia | 0 | 0 | 1643 | 0 | 3379 | 6239 | 0 | 0 | 950 | 3825 | 7760 | 11569 | 0 | 0 | 6719 |
| Tetracladium | 0 | 0 | 0 | 0 | 0 | 17299 | 0 | 1724 | 0 | 0 | 0 | 0 | 0 | 0 | 0 |
| Tetragoniomyces | 0 | 0 | 0 | 0 | 0 | 0 | 0 | 0 | 0 | 0 | 0 | 0 | 785 | 0 | 0 |
| Thelebolus | 0 | 0 | 0 | 0 | 0 | 3908 | 0 | 0 | 0 | 0 | 0 | 0 | 0 | 0 | 0 |
| Tolypocladium | 0 | 0 | 0 | 0 | 0 | 0 | 0 | 0 | 0 | 0 | 0 | 0 | 0 | 0 | 1171 |
| Tomentella | 0 | 7792 | 0 | 26857 | 3129 | 15863 | 0 | 0 | 7970 | 29360 | 41422 | 0 | 0 | 0 | 0 |
| Trechispora | 4203 | 630 | 0 | 0 | 826 | 0 | 0 | 0 | 5226 | 0 | 0 | 0 | 0 | 0 | 0 |
| Trematosphaeria | 0 | 1253 | 0 | 2957 | 0 | 0 | 3209 | 0 | 0 | 0 | 2482 | 0 | 10545 | 558 | 1436 |
| Trichocladium | 0 | 0 | 0 | 0 | 0 | 0 | 0 | 0 | 0 | 0 | 0 | 0 | 691 | 0 | 0 |
| Tricholoma | 0 | 0 | 0 | 0 | 0 | 0 | 0 | 0 | 6008 | 0 | 0 | 0 | 0 | 0 | 0 |
| Trichophaea | 0 | 0 | 0 | 0 | 0 | 0 | 0 | 0 | 0 | 10120 | 0 | 0 | 0 | 0 | 0 |
| Trichosporon | 766 | 0 | 949 | 0 | 728 | 0 | 0 | 0 | 928 | 1261 | 0 | 625 | 0 | 0 | 3059 |
| Tricladium | 0 | 0 | 0 | 0 | 0 | 0 | 0 | 1646 | 0 | 0 | 0 | 0 | 0 | 0 | 0 |
| Tuber | 0 | 0 | 0 | 2699 | 0 | 843 | 0 | 0 | 0 | 1294 | 0 | 0 | 0 | 0 | 0 |
| Tylospora | 0 | 0 | 0 | 0 | 0 | 0 | 0 | 0 | 0 | 0 | 15120 | 0 | 0 | 0 | 0 |
| Vargamyces | 0 | 0 | 0 | 0 | 0 | 1741 | 0 | 2155 | 0 | 0 | 0 | 0 | 0 | 0 | 0 |
| Varicosporium | 0 | 0 | 0 | 0 | 0 | 0 | 0 | 0 | 0 | 0 | 0 | 2685 | 0 | 0 | 0 |
| Wilcoxina | 0 | 0 | 0 | 0 | 0 | 0 | 0 | 0 | 0 | 0 | 37547 | 0 | 0 | 0 | 0 |
| Xenopolyscytalum | 0 | 0 | 0 | 0 | 0 | 0 | 0 | 0 | 0 | 0 | 0 | 523 | 0 | 0 | 0 |
| Xerocomellus | 1664 | 0 | 0 | 1187 | 517 | 0 | 0 | 0 | 0 | 448 | 0 | 0 | 0 | 0 | 8325 |
| Xerocomus | 0 | 0 | 0 | 0 | 0 | 0 | 0 | 0 | 5358 | 0 | 0 | 0 | 0 | 0 | 0 |
| Unknown | 1962 | 30898 | 54991 | 23494 | 69049 | 43675 | 45114 | 106759 | 21377 | 43569 | 32676 | 125187 | 29378 | 63902 | 31096 |
| **Total** | **260000** | **260000** | **260000** | **260000** | **260000** | **260000** | **260000** | **260000** | **260000** | **260000** | **260000** | **260000** | **260000** | **260000** | **260000** |

**References**

1. Tonge, D.P., C.H. Pashley, and T.W. Gant, *Amplicon-based metagenomic analysis of mixed fungal samples using proton release amplicon sequencing.* PLoS One, 2014. **9**(4): p. e93849.

2. Bell, T.H., et al., *Early rhizosphere microbiome composition is related to the growth and Zn uptake of willows introduced to a former landfill.* Environ Microbiol, 2015. **17**(8): p. 3025-38.

3. Blaya, J., et al., *Microbiota Characterization of Compost Using Omics Approaches Opens New Perspectives for Phytophthora Root Rot Control.* PLoS One, 2016. **11**(8): p. e0158048.

4. Brown, S.P., et al., *Deep Ion Torrent sequencing identifies soil fungal community shifts after frequent prescribed fires in a southeastern US forest ecosystem.* FEMS Microbiol Ecol, 2013. **86**(3): p. 557-66.

5. Geml, J., et al., *Large-scale fungal diversity assessment in the Andean Yungas forests reveals strong community turnover among forest types along an altitudinal gradient.* Mol Ecol, 2014. **23**(10): p. 2452-72.

6. Kemler, M., et al., *Ion Torrent PGM as tool for fungal community analysis: a case study of endophytes in Eucalyptus grandis reveals high taxonomic diversity.* PLoS One, 2013. **8**(12): p. e81718.

7. Li, L., et al., *Microbial composition and diversity are associated with plant performance: a case study on long-term fertilization effect on wheat growth in an Ultisol.* Appl Microbiol Biotechnol, 2017. **101**(11): p. 4669-4681.

8. Miura, T., et al., *Diversity of Fungi on Decomposing Leaf Litter in a Sugarcane Plantation and Their Response to Tillage Practice and Bagasse Mulching: Implications for Management Effects on Litter Decomposition.* Microb Ecol, 2015. **70**(3): p. 646-58.

9. Morgado, L.N., et al., *Summer temperature increase has distinct effects on the ectomycorrhizal fungal communities of moist tussock and dry tundra in Arctic Alaska.* Glob Chang Biol, 2015. **21**(2): p. 959-72.

10. Semenova, T.A., et al., *Long-term experimental warming alters community composition of ascomycetes in Alaskan moist and dry arctic tundra.* Mol Ecol, 2015. **24**(2): p. 424-37.

11. Urbina, H., et al., *Specificity in Arabidopsis thaliana recruitment of root fungal communities from soil and rhizosphere.* Fungal Biol, 2018. **122**(4): p. 231-240.

12. Arbefeville, S., A. Harris, and P. Ferrieri, *Comparison of sequencing the D2 region of the large subunit ribosomal RNA gene (MicroSEQ®) versus the internal transcribed spacer (ITS) regions using two public databases for identification of common and uncommon clinically relevant fungal species.* Journal of Microbiological Methods, 2017. **140**: p. 40-46.

13. DEMİREL, R., *Comparison of rDNA regions (ITS, LSU, and SSU) of some Aspergillus, Penicillium, and Talaromyces spp.* Turkish Journal of Botany, 2016. **40**: p. 576-583.

14. Brown, S.P., A.R. Rigdon-Huss, and A. Jumpponen, *Analyses of ITS and LSU gene regions provide congruent results on fungal community responses.* Fungal Ecology, 2014. **9**: p. 65-68.

15. Porras-Alfaro, A., et al., *From genus to phylum: large-subunit and internal transcribed spacer rRNA operon regions show similar classification accuracies influenced by database composition.* Applied and environmental microbiology, 2014. **80**(3): p. 829-840.

16. Tedersoo, L., et al., *Shotgun metagenomes and multiple primer pair-barcode combinations of amplicons reveal biases in metabarcoding analyses of fungi.* MycoKeys, 2015. **10**: p. 1-43.

17. Bonito, G., et al., *Plant host and soil origin influence fungal and bacterial assemblages in the roots of woody plants.* Molecular ecology, 2014. **23**(13): p. 3356-3370.

18. Schlaeppi, K., et al., *High-resolution community profiling of arbuscular mycorrhizal fungi.* The New phytologist, 2016. **212**(3): p. 780-791.

19. George, P.B.L., et al., *Primer and Database Choice Affect Fungal Functional but Not Biological Diversity Findings in a National Soil Survey.* 2019. **7**(173).

20. Sulaiman, I.M., E. Jacobs, and S.B. Simpson, *Genetic Characterization of Fungi Isolated from Environmental Samples: A Molecular Surveillance Study of Public Health Importance.* J AOAC Int, 2019. **102**(3): p. 975-976.

21. Bissett, A., et al., *Introducing BASE: the Biomes of Australian Soil Environments soil microbial diversity database.* Gigascience, 2016. **5**: p. 21.

22. Yan, D., et al., *High-throughput eDNA monitoring of fungi to track functional recovery in ecological restoration.* Biological Conservation, 2018. **217**: p. 113-120.

23. White, T.J., et al., *38 - AMPLIFICATION AND DIRECT SEQUENCING OF FUNGAL RIBOSOMAL RNA GENES FOR PHYLOGENETICS*, in *PCR Protocols*, M.A. Innis, et al., Editors. 1990, Academic Press: San Diego. p. 315-322.

24. Op De Beeck, M., et al., *Comparison and validation of some ITS primer pairs useful for fungal metabarcoding studies.* PloS one, 2014. **9**(6): p. e97629-e97629.

25. Issakainen, J., et al., *Relationship of Scedosporium prolificans with Petriella confirmed by partial LSU rDNA sequences.* Mycological Research, 1999. **103**(9): p. 1179-1184.

26. Afgan, E., et al., *The Galaxy platform for accessible, reproducible and collaborative biomedical analyses: 2018 update.* Nucleic Acids Research, 2018. **46**(W1): p. W537-W544.

27. Edgar, R.C., *UPARSE: highly accurate OTU sequences from microbial amplicon reads.* Nat Methods, 2013. **10**(10): p. 996-8.

28. Edgar, R.C., *SINTAX: a simple non-Bayesian taxonomy classifier for 16S and ITS sequences.* 2016: p. 074161.

29. Edgar, R.C., *Accuracy of taxonomy prediction for 16S rRNA and fungal ITS sequences.* PeerJ, 2018. **6**: p. e4652.

30. Edgar, R.C. *Sample pooling*. USEARCH 2019 25/11/19]; Available from: <https://drive5.com/usearch/manual/pool_samples.html>.

31. Edgar, R.C., *Personal communication*. 2017.

32. Edgar, R.C. *UNOISE algorithm*. USEARCH 2019 24/02/20]; Available from: <https://drive5.com/usearch/manual/unoise_algo.html>.

33. Edgar, R.C. and H. Flyvbjerg, *Octave plots for visualizing diversity of microbial OTUs.* bioRxiv, 2018: p. 389833.

34. Oksanen, J., et al., *Community ecology package.* 2013: p. 2.0-2.

35. Jost, L., *Entropy and diversity.* 2006. **113**(2): p. 363-375.

36. Wheeler, B., M. Torchiano, and M.M.J.R.p.v. Torchiano, *Package ‘lmPerm’.* 2016: p. 1-1.

37. De Caceres, M., F. Jansen, and M.M.J.i. De Caceres, *Package ‘indicspecies’.* 2016. **8**: p. 1.

38. Nguyen, N.H., et al., *FUNGuild: An open annotation tool for parsing fungal community datasets by ecological guild.* Fungal Ecology, 2016. **20**: p. 241-248.

39. Huse, S.M., et al., *Ironing out the wrinkles in the rare biosphere through improved OTU clustering.* Environ Microbiol, 2010. **12**(7): p. 1889-98.

40. Edgar, R.C., *Accuracy of microbial community diversity estimated by closed- and open-reference OTUs.* PeerJ, 2017. **5**: p. e3889.

41. Edgar, R.C. *Read preparation: discard singletons*. USEARCH 2019; Available from: <https://drive5.com/usearch/manual/pipe_readprep_singles.html>.

42. Edgar, R.C. *Abundance and amplification bias in amplicon sequencing*. USEARCH 2019 25/11/19]; Available from: <https://drive5.com/usearch/manual/amplification_bias.html>.

43. GRIFFITH, G.W., O. CAVALLI, and A.P. DETHERIDGE, *An assessment of the fungal conservation value of Hardcastle Crags (Hebden Bridge, West Yorkshire) using NextGen DNA sequencing of soil samples*, in *Natural England Commissioned Reports*. 2019.

44. Edgar, R.C. *Comparing diversity between sample groups*. USEARCH 2019; Available from: <https://drive5.com/usearch/manual/diversity_metrics_compare_groups.html>.
